# ﻿Taxonomic reassessment of the *Lycodon rufozonatus* species complex (Serpentes, Colubridae), with re-evaluation of *Dinodon rufozonatum walli*, and description of a new species from north-central Vietnam

**DOI:** 10.3897/zookeys.1251.157817

**Published:** 2025-09-15

**Authors:** Tan Van Nguyen, Nikolay A. Poyarkov, Gernot Vogel

**Affiliations:** 1 The School of Medicine & Pharmacy, Duy Tan University, Da Nang, 550000, Vietnam; 2 Center for Entomology & Parasitology Research, Duy Tan University, Da Nang, 550000, Vietnam; 3 Department of Vertebrate Zoology, Lomonosov Moscow State University, Leninskiye Gory, GSP–1, Moscow 119991, Russia; 4 Joint Vietnam - Russia Tropical Science and Technology Research Centre, Nghia Do, Hanoi, Vietnam; 5 Society for South East Asian Herpetology, Im Sand-3, D-69115 Heidelberg, Germany

**Keywords:** Asia, cryptic species, cytochrome *b*, morphology, taxonomy, *Lycodon duytan* sp. nov.

## Abstract

The Red-banded Wolf Snake, *Lycodon rufozonatus* Cantor, 1842 has a complex taxonomic history. In this study, an integrative taxonomic approach is applied, incorporating morphological analyses, cytochrome *b* mitochondrial gene sequencing, and a re-examination of available type material to clarify the longstanding taxonomic uncertainties within *Lycodon rufozonatus* species complex. Our findings restrict the distribution of *Lycodon rufozonatus* to mainland China, Taiwan, the Korean Peninsula, Russia (southern Primorsky Krai), Japan (Tsushima Islands, Nagasaki), and northeastern Vietnam. Additionally, *Dinodon rufozonatum walli* Stejneger, 1907, previously considered a subspecies, is elevated to full species status as *Lycodon walli***stat. nov.**, endemic to the Miyako and Yaeyama Islands, Okinawa, Japan. Furthermore, a new cryptic species is described from north-central Vietnam, *Lycodon duytan***sp. nov.**, which is distinguished from *L. rufozonatus* and *L. walli***stat. nov.** by differences in body scalation, colouration, and the number of keeled dorsal scales. Our findings highlight the underestimated diversity within *Lycodon* and contribute to a more refined understanding of its taxonomy. This study increases the total number of recognised *Lycodon* species to 78, underscoring the importance of integrative approaches in resolving taxonomic complexities within the genus.

## ﻿Introduction

The genus *Lycodon* Fitzinger, 1826, commonly known as wolf snakes, represents one of the most diverse genera within the family Colubridae, Oppel, in Tropical Asia. Currently, it comprises 77 recognised species with a broad distribution extending from eastern Iran through China and Japan, southward to the Philippines and the Indo-Australian Archipelago. These snakes predominantly inhabit tropical to subtropical Asian forests at low (<500 m asl) to mid (<1,000 m asl) elevations (Fitzinger 1826; Nguyen et al. 2024a, b, 2025a, b; Vogel et al. 2024, 2025; Lee et al. 2025; Nguyen and Vogel 2025; Uetz et al. 2025). The application of integrative taxonomic approaches has significantly reshaped the systematics of *Lycodon* over the past decade, leading to the discovery of previously unrecognised taxa and the revision of several species complexes. Notable examples include the *Lycodon fasciatus* complex (e.g., Wang et al. 2021; Nguyen et al. 2024a, 2025a; Vogel et al. 2024, 2025), the *L. paucifasciatus* complex (e.g., Nguyen et al. 2022; Nguyen and Vogel 2025), the *L. subcinctus* complex (e.g., Nguyen et al. 2024b, 2025b), and the *L. striatus* complex (e.g., Amarasinghe et al. 2023).

**Figure 1.**
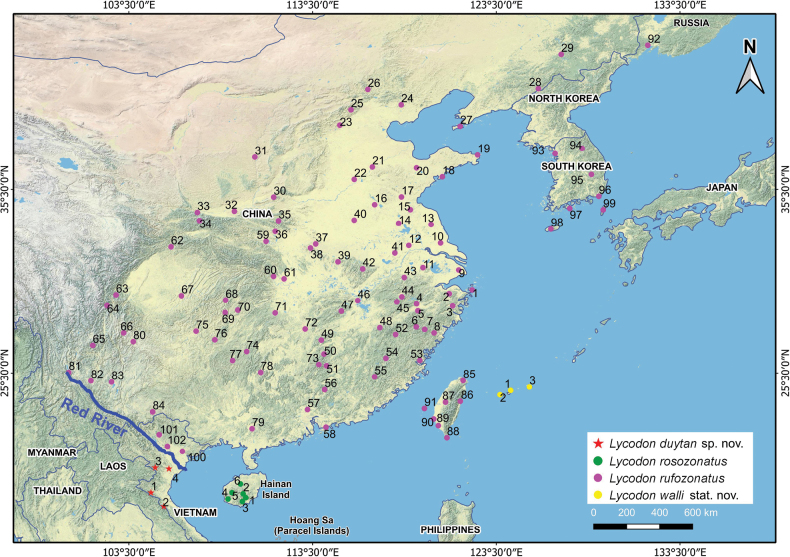
Distribution ranges of the *Lycodon rufozonatus* species complex in East Asia and Vietnam. Notes: numbers indicate different localities where the species have been recorded (see Suppl. material 1: table S7 for the details of localities).

Among the widely distributed species in East Asia, *Lycodon rufozonatus* Cantor, 1842 has received comparatively little taxonomic attention. This species is characterised by the following morphological features: a large body with a maximum total length of up to 1100 mm; smooth dorsal scales (rarely weakly keeled); 17 (19) midbody scale rows; a body pattern consisting of red or pinkish crossbands on a black or brown background; and a pale, inverted V-shaped marking on the nape (Pope 1935; Zhao et al. 1998; Zhao 2006; Nguyen and Vogel 2025; this study). *Lycodon rufozonatus* was originally described from Chusan (now Zhoushan City), Zhejiang Province, China (Cantor 1842). It has been reported from Russian Far East, the Korean Peninsula, southern and western Japan, China, Taiwan, and Vietnam (Fig. 1; Pope 1935; Zhao 2006; Wu et al. 2023; Shin et al. 2024; Uetz et al. 2025). This species occupies a wide range of habitats, including montane forests, residential areas adjacent to mountains, rice paddies, and riparian zones (Zhao et al. 1998; Shin et al. 2024). It is known to prey on fish, frogs, lizards, snakes, young birds, and small mammals (Zhao et al. 1998; this study). Due to the high degree of morphological similarity within this species complex, geographic variation in *Lycodon rufozonatus* has never been comprehensively examined across its range, leaving its taxonomic status unresolved. Currently, *Lycodon rufozonatus* has six subjective synonyms: *Dinodon cancellatum* Duméril & Bibron, *Coronella striata* Hallowell, Dinodon rufozonatus var. formosana Boettger, *Dinodon rufozonatum walli* Stejneger, *Dinodon rufozonatum williamsi* Schmidt, and *Dinodon rufozonatum yunnanense* Mell (see Pope 1935; Zhao et al. 1998; Wallach et al. 2014; Uetz et al. 2025). A summary of the taxonomic history of *Lycodon rufozonatus* is provided in Table 1. Under the current classification framework, *Lycodon rufozonatus* is currently recognised as comprising two subspecies: the nominate form, *L. r. rufozonatus*, distributed across continental East Asia, and *L. r. walli*, occurring on Miyako and Yaeyama islands in the southern Ryukyus (Stejneger 1907; Takara 1962; Zhao et al. 1998; Goris and Maeda 2004). In this study, we examine name-bearing type specimens and reassess the taxonomy of the *Lycodon rufozonatus* species complex by integrating historical collection materials, newly collected specimens, literature data, and molecular evidence.

**Table 1.** Species-level scientific names erected for the members of the *Lycodon rufozonatus* species complex.

No.	Authority	Original taxon name	Type locality	Present taxonomy	Proposed taxonomy
1	Cantor, 1842	*Lycodon rufo-zonatus*	Zhoushan, Zhejiang, China	*Lycodon rufozonatus*	*Lycodon rufozonatus*
2	Duméril & Bibron, 1854	*Dinodon cancellatum*	probably China	synonym of *Lycodon rufozonatus*	*Lycodon rufozonatus*
3	Hallowell, 1856	*Coronella striata*	Ningbo, Zhejiang, China	synonym of *Lycodon rufozonatus*	*Lycodon rufozonatus*
4	Boettger, 1885	Dinodon rufozonatus var. formosana	Taiwan	synonym of *Lycodon rufozonatus*	*Lycodon rufozonatus*
5	Stejneger, 1907	*Dinodon rufozonatum walli*	Ishigaki, Yaeyama, Ryukyu, Japan	synonym of *Lycodon rufozonatus* (consider as subspecies of *L. rufozonatus*)	*Lycodon walli* stat. nov.
6	Schmidt, 1925	*Dinodon rufozonatum williamsi*	Changsha, Hunan, China	synonym of *Lycodon rufozonatus*	*Lycodon rufozonatus*
7	Mell, 1931	*Dinodon rufozonatum yunnanense*	Dali, Yunnan, China	synonym of *Lycodon rufozonatus*	*Lycodon rufozonatus*
8	This paper	Lycodon cf. rufozonatus	Pu Mat NP, Nghe An, Vietnam	Lycodon cf. rufozonatus	*Lycodon duytan* sp. nov.

## ﻿Materials and methods

### ﻿Morphology

We examined a total of 78 specimens of *Lycodon rufozonatus* auctorum for external morphological characters, including the holotype of *Lycodon rufozonatus*; syntypes of *Coronella striata*; the holotype of Dinodon rufozonatus var. formosana; the paratype of *Dinodon rufozonatum williamsi*; the syntypes of *Dinodon rufozonatus yunnanense*; and the holotype of *Dinodon rufozonatum walli* (see Suppl. material 1: table S1). Most of the morphological data that we collected originate from preserved specimens in natural history collections, except for three individuals of *Lycodon* sp. from Nghe An and Thanh Hoa provinces, Vietnam, were examined in the field and which were collected temporarily to take relevant measurements and then released at their point of capture. We combined our material with morphological data on 51 additional *Lycodon rufozonatus* specimens published in the literature from Duméril et al. (1854); Stejneger (1907); Maki (1931); Takara (1962); and Yang and Rao (2008).

A total of 54 morphological characters were recorded for each specimen (following Nguyen et al. 2024a). Measurements, except body and tail lengths, were taken with slide callipers to the nearest 0.1 mm. Snout-vent length and tail length were taken with a tape measure or a metallic ruler. Relative tail length was calculated as the length of the tail compared to the total length of the specimen. The number of ventral scales was counted according to the method described by Dowling (1951). Half ventrals were not counted except when they were present on both sides (divided ventrals). The terminal scale is not included in the number of subcaudals. Total body scales include the sum of the number of ventral and subcaudal scales and the cloacal plate (considered a single scale regardless of whether the plate was single or paired). The subcaudal ratio (given as a percentage) was calculated by taking the number of subcaudal scales and dividing that value by the number of total body scales. Dorsal scale row counts were taken at one head length behind the head, at midbody (i.e., at half of the value of snout-vent length), and at one head length anterior to the cloaca. We considered infralabials as shields along the border of the mouth that were fully covered by an accompanying supralabial. Values for paired head characters are given in left/right order. We define the loreal scale as any single, elongated scale located between the posterior edge of the nasal scale and the orbit. In other words, if there is a fusion between the ‘true’ loreal and the preocular scale, that scale was considered to be the loreal. Pale-coloured dorsal bands on the body and tail were counted only on the left side of each specimen. Hardly visible or incomplete bands were counted as one band. Fused bands were counted as two bands. The collar on the neck was not counted as a dorsal band, and any dorsal bands that covered the cloacal plate were added to the bands of the body. Sometimes dorsal bands on *Lycodon* species form a “Y”-shaped marking, and in such cases, the number of bands on one side might be higher than those on the other side of the body. The band covering the tail tip was not included in the number of bands. Morphological measurements (all in mm) and counts included: snout-vent length (**SVL**) measured from the tip of the snout to the posterior edge of the cloaca , tail length (**TaL**) measured from the posterior edge of the cloaca to the tail tip , total length (**TL**) measured by adding **SVL** and **TaL** together , and relative tail length (**TaL/TL**). Meristic character acronyms mentioned in-text include the number of ventral scales (**VEN**) , number of subcaudal scales (**SC**) , loreal scale (**Lor**) , supralabials (**SL**) , supralabials in contact with the eye (**SL-E**) , infralabials (**IL**) , anterior temporal (**AT**) , posterior temporal (**PT**) , preocular (**PrO**) , postocular (**PoO**) , dorsal scale rows at head (**ASR**) , dorsal scale rows at midbody (**MSR**) , dorsal scale rows at vent (**DSR**) , keeled in midbody dorsal scale rows (**KSD**) , body bands (**BB**) , and tail bands (**TB**).

To compare quantitative variation across *Lycodon rufozonatus*, we ran a series of univariate and multivariate statistical comparisons to assess whether each of the three species-level lineages occupied distinct morphological clusters and possessed statistically significant differences from one another. We a priori assigned specimens of *Lycodon rufozonatus* sensu lato to one of three species-level lineages (namely, Lycodon cf. rufozonatus from north-central Vietnam, *Dinodon rufozonatum walli* from the southern Ryukyu Islands in Japan, and *Lycodon rufozonatus*) based on external morphological features and geographic range prior to running statistical comparisons. Specimens with broken tails or missing data for one or more morphological characters were pruned from the multivariate analysis but were kept for univariate comparisons. We subjected each morphological character in our dataset to a series of Shapiro-Wilks tests and Levene’s tests to ensure normality and homoscedasticity, respectively, and performed Student’s unpaired *t*-tests between sexes to assess whether any significant sexual dimorphism exists across the dataset. We found that most morphological characters satisfied these statistical assumptions and did not display any significant dimorphism. Therefore, in order to maximise our sample size and permit the inclusion of juvenile specimens, we pooled both male and female specimens of *Lycodon rufozonatus* together in all subsequent statistical tests. We performed a one-way analysis of variance (ANOVA) to determine whether significant differences existed across the morphological data, then used a Tukey’s HSD post-hoc test to compare the mean differences of each character between species pairs. We combined ten morphological characters for use in multivariate comparisons and subjected 126 total specimens (11 “cf. rufozonatus from north-central Vietnam”, 40 *walli*, and 75 *rufozonatus*) to a Principal Components Analysis (PCA) to examine whether the three lineages clustered in separate regions of morphospace (see Suppl. material 1: table S2 for a list of characters used in this analysis). All statistics were conducted using base functions in R v. 4.4.0 (R-Core Team 2024). The first two principal components were plotted using the R package ggplot2 (Wickham 2016). We consider all morphological differences to be statistically significant if their *P*-values were ≤ 0.05.

### ﻿DNA isolation and sequencing

Total genomic DNA was extracted from ethanol-preserved muscle or liver tissues using standard phenol-chloroform extraction procedures (Sambrook et al. 1989), followed by isopropanol precipitation. The total DNA concentration was estimated in 1 μL using a NanoDrop 2000 (Thermo Scientific, USA) and consequently adjusted to 100 ng DNA/μL. We amplified the partial sequence of the cytochrome b (cyt b) mtDNA gene using the primers H14910 5'-GACCTGTGATMTGAAAAACCAYCGTT -3' and THRSN2 5'-CTTTGGTTTACAAGAACAATGCTTTA-3' (Burbrink et al. 2000).

PCR was performed in 20-μL reactions using 50 ng of genomic DNA, 10 nmol of each primer, 15 nmol of each dNTP, 50 nmol of additional MgCl2, Taq PCR buffer (10 mmol/L Tris-HCl, pH 8.3, 50 mmol/L KCl, 1.1 mmol/L MgCl2, and 0.01% gelatin), and 1 U of Taq DNA polymerase. We used the same pair of primers both for PCR and sequencing. The PCR conditions were: denaturation at 94 °C for 3 min, followed by 35 cycles at 94 °C for 30 s, 52 °C for 40 s and 72 °C for 90 s, with a final extension step at 72 °C for 10 min. PCR products were visualised by agarose electrophoresis in the presence of ethidium bromide and consequently purified using 2 μL from a 1:4 dilution of ExoSapIt (Amersham, UK) per 5 μL of PCR product prior to cycle sequencing. Sequence data collection and visualisation were performed on an ABI 3730xl automated sequencer (Applied Biosystems, USA) in Evrogen Inc, Moscow. The obtained sequences were aligned and deposited in GenBank under the accession numbers PQ863685–PQ863686.

### ﻿Molecular phylogeny

To estimate the phylogenetic relationships of the genus *Lycodon*, we used the newly obtained cyt *b* sequences together with previously published sequences of *Lycodon rufozonatus* (19 sequences in total), as well as representative sequences of 51 species of *Lycodon*. *Oligodon maculatus* (Taylor) was used to root the tree (Suppl. material 1: table S3).

We initially aligned the nucleotide sequences in MAFFT online (Katoh et al. 2019) with default parameters and subsequently checked them by eye in BioEdit v. 7.0.5.2 (Hall 1999) and adjusted them when required. The mean uncorrected genetic *p*-distances between sequences were calculated with MEGA 6.0 with the pairwise deletion option (Tamura et al. 2013) based on cyt *b* sequences of genus *Lycodon*. The best-fit substitution models for the data set were selected for genes and codon positions using PartitionFinder v. 2.1.1 (Lanfear et al. 2012) with the Akaike information criterion (AIC), which selected GTR+I for the first and second codon positions of cyt *b*, and GTR+G for the third codon positions of cyt *b*.

Phylogenetic trees were estimated for the combined mitochondrial DNA fragments (cyt *b*) data set. We inferred the matrilineal genealogy of *Lycodon* using Bayesian inference (BI) and maximum likelihood (ML) approaches. We used the IQ-TREE online server (Nguyen et al. 2015) to generate the ML tree and assessed the confidence in tree topology by 1000 bootstrap replications (BS). We conducted BI in the terminal version of MrBayes 3.1.2 (Huelsenbeck and Ronquist 2001). Metropolis-coupled Markov chain Monte Carlo (MCMCMC) analyses were run with one cold chain and three heated chains for 40 million generations and sampled every 40,000 generations. The run was checked to ensure the effective sample sizes (ESS) were all above 200 by exploring the likelihood plots using TRACER v. 1.7 (Rambaut et al. 2018). We discarded the initial 1000 trees as burn-in. We assessed the confidence in tree topology by the posterior probability (PP) of the nodes (Huelsenbeck and Ronquist 2001). The ML and BI trees were visualised and edited using FigTree v. 1.4.4 (Rambaut 2018) and tvBOT v. 2.6.1 (Xie et al. 2023; https://www.chiplot.online/tvbot.html).

### ﻿Species delimitation

To identify the number of Molecular Operational Taxonomic Units (MOTUs) represented by *Lycodon* sp. from north-central Vietnam and other *Lycodon* spp. of the *L. rufozonatus* species group, delimitation analyses were performed: Assemble Species by Automatic Partitioning (ASAP) (Puillandre et al. 2021) and Bayesian implementation of Poisson Tree Processes model (bPTP) (Zhang et al. 2013). These species delimitations were regarded as preliminary hypotheses. Then the distinctions based on morphological characters were used to treat them as either distinct species or conspecific lineages.

To apply ASAP, the sequence alignment of the cyt *b* was uploaded at the ASAP web (https://bioinfo.mnhn.fr/abi/public/asap/asapweb.html). The analysis with Jukes-Cantor distance (JC69), Kimura (K80) ts/tv 2.0 and Simple Distance (p-distances) was employed with the same settings (see Suppl. material 1: table S4). The second species delimitation approach was the Bayesian PTP (bPTP), for which analyses were performed on the online server (https://species.h-its.org/ptp/) using the ML trees (see Suppl. material 1: table S4). Data sets were run for 1,000,000 generations with a thinning of 1,000 and a burn-in of 0.1, then assessed convergence visually using the MCMC iteration v log-likelihood plots generated automatically. This incorporates the potential divergence in intraspecific diversity to the PTP and implements a fast method to compute the maximum likelihood delimitation from an inferred phylogenetic tree of the samples.

### ﻿Other abbreviations

Mt = Mountains; NP = National Park; NR = Nature Reserve; RF = Reserved Forest; WS = Wildlife Sanctuary; asl = above sea level. Refer to Suppl. material 1: table S5 for a list of museum and natural history collection acronyms used in this study.

## ﻿Results

### ﻿Species delimitation and molecular phylogeny

The ASAP and PTP analyses indicated the number of MOTUs (excluding the outgroups) was not equal to those identified by morphospecies. The results of the distance-based approach using ASAP with the JC69 and K80 evolution models recovered 53 MOTUs, respectively, as well as tree-based species delimitations indicating 52 MOTUs (Suppl. material 1: table S4). By comparing them with morphology-based delimitation, we concluded that there were 51 confirmed candidate species being treated as distinct MOTUs and the uncertain MOTUs (ABGD: *Lycodon rufozonatus* species group, *L. alcalai* Ota & Ross from Bataan Batanes, Philippines, and *L. chrysoprateros* Ota & Ross from Dalupiri Cagayan, Philippines; PTP: *L. alcalai* from Bataan Batanes, Philippines, and *L. chrysoprateros* from Dalupiri Cagayan, Philippines). Thus, species delimitation of partial cyt *b* supports the hypothesis of morphospecies between *Lycodon* sp. from north-central Vietnam and other species.

**Figure 2.**
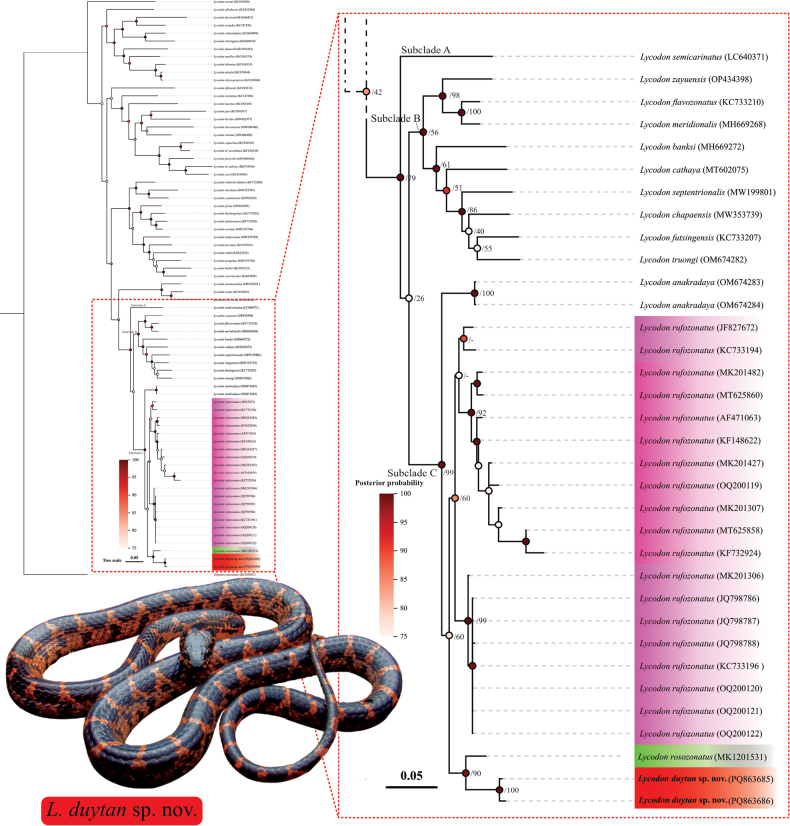
Representative BI tree of the genus *Lycodon*, based on the partial sequences of cyt b gene. Each clade colour indicated results of species delimitation analyses using ABGD and bPTP. Numbers on the branches indicate bootstrap values.

The phylogenetic trees of partial *cyt b* sequences of *Lycodon* species were constructed using Bayesian inference (BI) and maximum likelihood (ML) methods (Suppl. material 1: figs S1, S2), with *Oligodon maculatus* as the outgroup. The ML and BI analyses produced similar topologies. According to our phylogenetic tree, the upper clade showed lower support; however, in this study, we focus only on the relationships among *Lycodon* sp. from north-central Vietnam and other species within the *L. rufozonatus* species group (Fig. 2). Subclade C includes *Lycodon anakradaya* Nguyen, Duong, Wood & Grismer, *L. rufozonatus*, *L. rosozonatus* (Hu & Zhao), and *Lycodon* sp. from north-central Vietnam and is strongly supported as monophyletic (1.00/99; Bayesian posterior probability / ML bootstrap values). This clade forms a polytomy with two other subclades: subclade A (*Lycodon semicarinatus* (Cope)) and subclade B (*L. zayuensis* Jiang, Wang, Jin & Che, *L. flavozonatus* (Pope), *L. meridionalis* (Bourret), *L. banksi* Luu, Bonkowski, Nguyen, Le, Calame & Ziegler, *L. cathaya* Wang, Qi, Lyu, Zeng & Wang, *L. chapaensis* (Angel & Bourret), *L. septentrionalis* (Günther), *L. futsingensis* (Pope), and *L. truongi* Nguyen, Duong, Wood & Grismer), which is also well supported (1.00/79).

Within subclade C, *Lycodon anakradaya* was recovered as a sister to all remaining species of the clade, with strong support (1.00/99). *L. rufozonatus* clustered as a sister to *Lycodon* sp. from north-central Vietnam and *L. rosozonatus*. The *L. rufozonatus* clade is divided into two subgroups, consistent with ASAP analysis (support 0.85/60). Two sequences of *Lycodon* sp. from north-central Vietnam collected from Nghe An Province (PQ863685) and Ninh Binh Province (PQ863686) cluster together with strong support in both BI and ML analyses (0.99/90).

### ﻿Molecular divergence

The uncorrected *p*-distances for the partial cyt *b* gene among the *Lycodon* species group examined here are presented in Suppl. material 1: table S6. The two sequences of *Lycodon* sp. from Nghe An Province (PQ863685) and Ninh Binh Province (PQ863686) show interspecific distances of *p* = 0.2%. Interspecific distances varied from *p* = 3.17–13.25% (3.17–3.39% between *Lycodon* sp. from north-central Vietnam and *L. rosozonatus*; 3.17–4.74% between *Lycodon* sp. from north-central Vietnam and *L. rufozonatus*).

### ﻿Morphological analyses

The principal component analysis (PCA) of three closely related lineages of *Lycodon* sp. from north-central Vietnam, *L. rufozonatus* and *L. walli*, is shown in Suppl. material 1: fig. S3. Based on four candidate morphological characters, the clusters of three species of *Lycodon* in the plot of PC1 and PC2 can be clearly separated, which together explain the largest part of the variance. PC1–PC2 accounted for 75.52% of the morphological variation (Suppl. material 1: table S2). The factor loadings of PC1 accounted for 44.26% and were moderately loaded on two characters (BB and TB). The PC2 accounted for 31.27% of the variation and loaded heavily and moderately for SC and BB, respectively.

### ﻿Taxonomic conclusions

Based on morphological analyses, including the direct examination of type series for *Coronella striata*, *Dinodon rufozonatus formosana*, *D. r. williamsi*, and *D. r. yunnanense*, we found these taxa to be closely related and best regarded as conspecific, thereby supporting their recognition as junior subjective synonyms of *Lycodon rufozonatus*. However, two distinct populations – one from the Miyako and Yaeyama islands, Japan, corresponding to *Lycodon rufozonatus walli*, and the other from north-central Vietnam – exhibit morphological differences from *L. rufozonatus* and are geographically allopatric. Concurrently, molecular phylogenetic analyses reveal that the *Lycodon* sp. population from north-central Vietnam is sister to *L. rosozonatus* and clusters within the clade containing *L. rufozonatus*. Therefore, based on an integrative approach combining morphological and molecular evidence, we consider *Lycodon walli* to warrant recognition as a full species. Additionally, the *Lycodon* sp. population in north-central Vietnam, previously referred to as Lycodon cf. rosozonatus, should be described as a new species, detailed below.

### ﻿Species descriptions

#### ﻿ Lycodon rufozonatus

Taxon classificationAnimalia

Cantor, 1842

B0F727D8-A135-52E9-ACB6-875E50E43E07

Tables 2, Fig. 3; Suppl. material 1: table S1, figs S4–S11

Lycodon rufo-zonatus Cantor (1842: 483) — Holotype: NHMUK [The Natural History Museum, London, UK] 1843.7.21.36 donated by T. Cantor. Type locality: Chusan (now Zhoushan) Islands, Zhejiang Province, China.
Dinodon cancellatum Duméril & Bibron in Duméril et al. 1854: 447. Holotype: not traced. Type locality: unknown, probably from China (see Stejneger 1907).
Coronella striata Hallowell, 1856: 152. Syntypes: ANSP 3477–78. Type locality: Ningpo (now Ningbo), Zhejiang Province, China.
Dinodon rufozonatus var. formosana Boettger, 1885: 125. Holotype: SMF 18045. Type locality: Formosa, now Taiwan.
Dinodon rufozonatum williamsi Schmidt, 1925: 2. Holotype: AMNH 17453. Type locality: Changsha City, Hunan Province, China.
Dinodon rufozonatum yunnanense Mell, 1931: 2007. Syntypes: ZMB 52629–31, ZMB 27711. Type locality: Talifu (now Dali City), Yunnan Province, China.

##### Material examined.

A total of 61 specimens (32 males and 29 females) were examined; see in Suppl. material 1: table S1.

##### Referred materials.

A total of 14 specimens were used for reference (seven males, six females and one sex unknown) and were reported by Duméril et al. (1854), Maki (1931), and Yang and Rao (2008); see in Suppl. material 1: table S1.

##### Diagnosis.

Larger-sized species have a maximum snout-vent length of up to 1122 mm; a loreal slight entering the eye (rarely not); dorsal scales in 17 (19 or 21)–17 (19)–15 (16 or 17) rows, smooth throughout (rarely very faintly keeled posteriorly); 186–216 ventrals; 60–88 subcaudals, paired; cloacal plate undivided; eight supralabials with 3–5 touching the eye; one preocular, two postoculars; temporals 2+3; ground colour back with 60–106 red narrow crossbands on body and tail; ventral surface of body uniform cream, ventral surface of tail heavily dark speckled, not banded (based on Cantor 1842; Duméril et al. 1854; Hallowell 1856; Boettger 1885; Schmidt 1925; Mell 1931; Maki 1931; Yang and Rao 2008; this study).

##### Description of the holotype

**Figure 3.**
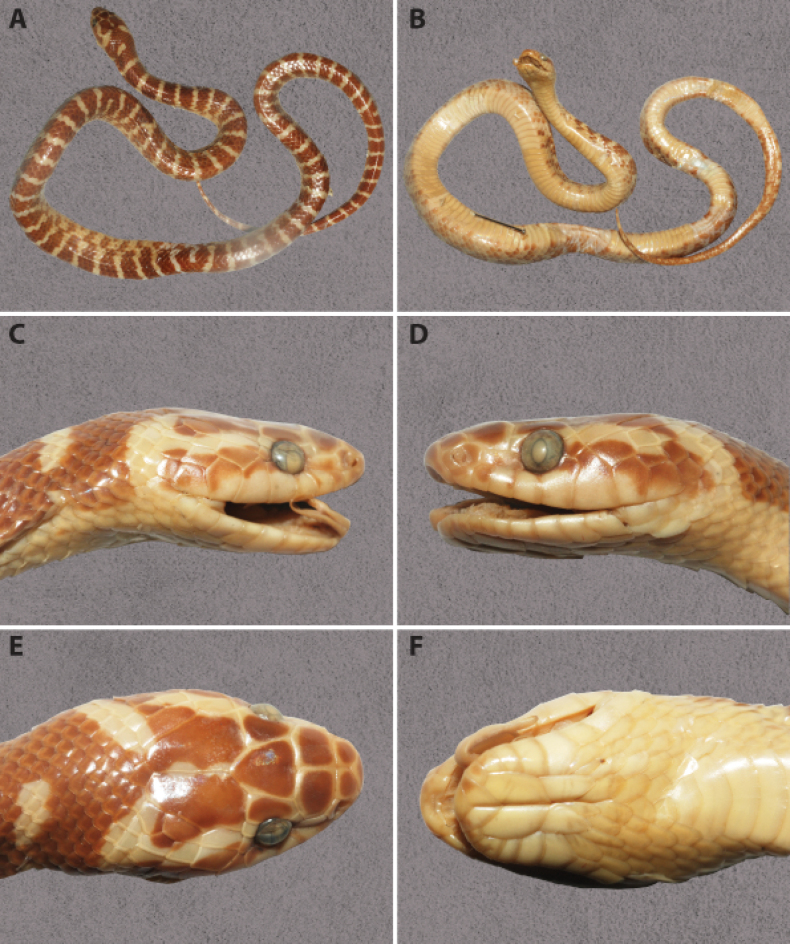
*Lycodon rufozonatus* in preservative – Specimen NHMUK 1843.7.21.36 (holotype, adult male): general dorsal view (A); general ventral view (B); lateral view of the head, right side (C); lateral view of the head, left side (D); dorsal view of the head (E); ventral view of the head (F). Photographs by GV.

(**Fig. 3)**: The body is robust and slightly laterally compressed. The tail is relatively long, thin, and tapering. The head is elongated, longer than wide, and moderately flattened, with a distinct separation from the neck. The snout is elongated, flattened, and slightly projects beyond the lower jaw. The nostrils are relatively large, positioned dorsolaterally, and round in shape. The eyes are relatively large, with vertical pupils.

***Body size*.**SVL 370 mm, TaL 92 mm; ratio TaL/TL 0.199.

***Body scalation*.** Dorsal scale rows 17–17–15, all smooth; scales of the vertebral row not enlarged; no apical pits; 198 ventrals; 74 subcaudals, all paired; cloacal plate undivided.

***Head scalation*.** Rostral heptagonal, wider than high, slightly visible from above; nasal single, elongated; nasal surrounded by the first two supralabials, rostral, internasal, and prefrontal; internasals two, curved, slightly wider than longer, in contact with rostral anteriorly, nasal, and prefrontal; two prefrontals, large, subrectangular, prefrontal slightly shorter than frontal; prefrontals in contact with internasals, nasals, preoculars, and frontal; frontal rather small, pentagonal, tapering posteriorly, shorter than the distance from tip of snout to frontal; parietals longer than wide, in contact approximately the length of the frontal; 1/1 supraocular, distinctly wider than high, in contact with prefrontal; 1/1 loreal, contacting eye; 1/1 preocular, large, higher than wide, in broad contact with prefrontal; subocular absent; 1/2 postoculars; 2+3 temporals; 8/8 supralabials, first and second in contact with nasal, second and third contact with loreal, third and fifth in contact with eye, six and seven largest; infralabials 10/10, first pair in broad contact with each other, first to fifth in contact with anterior pair of chin shields; posterior chin shields smaller than anterior ones, separated from each other by a pair of small scales.

***Colouration in preservative*.** The dorsal surface is blackish-brown, with 60 pale transverse crossbands on the body and 20 on the tail. The head is black, featuring a distinct inverted V-shaped marking on the nape. The ventral surface is cream-coloured, gradually becoming darker toward the cloaca, while the ventral surface of the tail is entirely dark.

##### General description and variation

(see Table 2; Suppl. material 1: table S1, figs S4–S11). Morphology variation based on 61 examined specimens as well as data morphology of 14 specimens were reported by Duméril et al. (1854), Maki (1931), and Yang and Rao (2008). The longest known specimen is 1,349 mm long (adult female; SVL 1145 mm, TaL 204 mm, ZMB 24830A). The longest known male is 1,323 mm long (SVL 1122 mm, TaL 201 mm; ZMB 19329). Body elongated; head distinct from neck, markedly flattened; eye medium; pupil vertically oval; rostral triangular, broader than high, clear visible from above; internasals as broad as long, approximately half the length of the prefrontal; prefrontal shorter than frontal; frontal hexagonal; parietals large, longer than wide; nasal divided; one loreal, nearly rectangular, narrowing posteriorly, protruding somewhat beneath preocular, usually entering the eye or not, not in entering with internasals; one (rarely absent or two) preocular; two (single) postoculars; two (single) anterior temporals; three posterior temporals (two); eight (seven or nice) supralabials, 1^st^ and 2^nd^SL in contact with the nasal, 2^nd^ and 3^rd^SL in contact with the loreal, 3^rd^–5^th^SL entering orbit, 6^th^ and 7^th^SL largest; ten (nice or 11) infralabials; first pair in contact each with other, 1^st^–5^th^IL in contact with anterior chin shields, 5^th^ and 6^th^IL largest; 17 (18, 19 or 21) dorsal scale rows at head, 17 (19) dorsal scale rows at midbody, 15 (16 or 17) dorsal scale rows at vent, the upper dorsal and vertebral scale rows entirely smooth or very faintly keeled posteriorly; ventrals 186–216 (199.60 ± 7.84, *n* = 75), without sexual dimorphism, vertebral scale slightly enlarged, distinctly angulate laterally; cloacal plate undivided; subcaudals 60–88 (74.30 ± 7.83, *n* = 63), without sexual dimorphism; relative tail length 0.151–0.237 (0.196 ± 0.021, *n* = 63), without sexual dimorphism.

**Table 2.** Comparison of morphological characters of *Lycodon duytan* sp. nov. with *L. walli* stat. nov., *L. rufozonatus*, and *L. rosozonatus*. Diagnostic characters distinguishing the new species from the other three species are indicated in bold.

Species	*L. duytan* sp. nov.	*L. walli* stat. nov.	*L. rufozonatus*	*L. rosozonatus*
**Max SVL** (**males**, **mm**)	890	**922**	**1122**	**1060**
**Max SVL** (**females**, **mm**)	980	**840**	**1145**	**866**
**TaL/TL**	0.183–0.227 (0.198 ± 0.013, *n* = 9)	**0.196–0.288** (**0.217 ± 0.016**, ***n* = 33**)	0.151–0.237 (0.196 ± 0.021, *n* = 63)	0.185–0.200 (0.191 ± 0.006, *n* = 5)
**VEN**	217–230 (225.09 ± 4.01, *n* = 11)	**164–198** (**187.95 ± 6.21**, ***n* = 40**)	**186–216** (**199.60 ± 7.84**, ***n* = 75**)	219–224 (221.89 ± 1.96, *n* = 9)
**SC**	80–95 (88.11 ± 5.64, *n* = 9)	71–90 (82.11 ± 4.37, *n* = 37)	**60–88** (**74.30 ± 7.83**, ***n* = 63**)	80–85 (82.50 ± 2.89, *n* = 4)
**VEN+SC**	299–325 (312.78 ± 8.73, *n* = 9)	**235–285** (**270.03 ± 9.45**, ***n* = 37**)	**247–297** (**273.21 ± 12.58**, ***n* = 63**)	301–309 (305.00 ± 4.62, *n* = 4)
**BB+TB**	54–72 (64.22 ± 6.63, *n* = 9)	**39–51** (**45.40 ± 3.36**, ***n* = 15**)	**60–106** (**79.06 ± 11.29**, ***n* = 66**)	**39**–**42 (40.80 ± 1.30**, ***n* = 5)**
**KSR**	4 or 5	**0**	**0**	2 or 3
**Body colour**	black	**black-grey or chocolate**	black	black
**Colour of bands**	pinkish orange	**grey-brown or dirty cream**	pinkish or reddish-brown	pinkish orange
**Distributions**	Vietnam (north-central)	Japan (endemic to Miyako & Yaeyama islands)	China including Taiwan Island, Korean Peninsula, Russia (Chernigovka), Japan (Nagasaki Islands), northeast Vietnam	China (endemic in Hainan Island)
**References**	This study	Stejneger (1907); Maki (1931); Takara (1962); This study	Duméril and Bibron (1854); Boettger (1885); Hallowell (1856); Stejneger (1907); Schmidt (1925); Mell (1931); Maki (1931); Takara (1962); Yang and Rao (2008); This study	Nguyen and Vogel (2025)

***Colouration*.** The dorsal surface of the body and tail is blackish, with 42–78 narrow red or orange-pink crossbands on the body and 15–30 on the tail. Each pale crossband is ~1–3 dorsal scales wide, interconnecting to divide the ground colour into elliptical patches. The ventral surface of the body is uniformly cream, while the ventral surface of the tail is heavily speckled with dark markings. The head is black, with conspicuously red-margined plates and a distinct inverted V-shaped marking on the nape. Pale stripes extend downward from the top of the temporal scales to the last supralabial scale.

##### Etymology.

The species name consists of two Latin adjectives, *rufus* (meaning red) and *zonatus* (meaning banded), literally meaning “red-banded”. We recommend the following common names for this species: Red-banded Wolf Snake (in English); Grosszahnnatter (in German); Северный краснопоясный волкозуб “Severyni krasnopoyasnyi volkozub” (in Russian); 赤链蛇 “Chì liàn shé” (in Chinese); 능구렁이 “Neung-guleong-I” (in South Korean); アカマダラ “Akamadara” (in Japanese); Rắn khuyết dải thân đỏ (in Vietnamese).

##### Distribution

(Fig. 1). **China**: This species is widely distributed across the country, occurring in the provinces of Anhui, Beijing, Chongqing, Fujian, Gansu, Guangdong, Guangxi, Guizhou, Hainan, Henan, Hebei, Hubei, Hunan, Jilin, Jiangsu, Jiangxi, Liaoning, Sichuan, Shandong, Shanghai, Shanxi, Shaanxi, Tianjin, Yunnan, and Zhejiang (Zhao and Adler 1993; Wang et al. 2021; this study). **Taiwan**: This species is very common and widely distributed throughout the island (C.W. You, pers. obs.). **South Korea**: It is likely to be common in the country and has been recorded in Incheon City, Gangwon Province, Seoul City, and Busan City (Shin et al. 2024; this study). **Japan**: Reported from Uotsuri and Tsushima Islands, Nagasaki Prefecture (Li et al. 2017; Morikawa and Inoue 2025). **Russia**: Recorded in Nezhino, Chernigovka, and the Posyet districts, all within the southern part of Primorsky Krai (Maslov and Kotlobay 1998; Li et al. 2017; Sundukov 2025). **Vietnam**: We confirm the occurrence of this species in northeastern Vietnam, including Tuyen Quang Province (Na Hang NR, based on specimen ROM 30814; see Suppl. material 1: fig. S9A), Vinh Phuc Province (Tam Dao NP, based on specimen ROM 34615; see Suppl. material 1: fig. S9B), and an individual observed in Tay Yen Tu NR, Bac Giang Province (see Suppl. material 1: fig. S11D). Additional records from previously reported locations are discussed below. **Laos**: The record from Xiengkhouang Province (Deuve 1970) was later revised as *Lycodon meridionalis* Bourret (Orlov and Ryabov 2004; this study).

##### Natural history notes.

This species is common in China, Taiwan, and South Korea but is rare in Russia, Japan, and Vietnam (Ananjeva et al. 2006; this study). It is an oviparous species occurring in a wide range of habitats, including plains, hills, and montane areas, from boreal to tropical forests. It is also found in villages and other rural areas, typically near water bodies, at elevations of ca 850–1,170 m asl. This species is primarily nocturnal and terrestrial but has been occasionally observed swimming. Its diet includes a wide range of vertebrates. Prey items include fish and toads, such as Duttaphrynus cf. gymnauchen (Bleeker) and *Bufo gargarizans* Cantor; frogs, including *Pelophylax nigromaculatus* (Hallowell), *Fejervarya limnocharis* (Gravenhorst), *F. kawamurai* Tjong, Matsui, Kuramoto, Nishioka & Sumida, Kaloula cf. pulchra Gray, Limnonectes cf. fujianensis Ye & Fei, and *Polypedates braueri* (Vogt); and lizards such as *Diploderma polygonatum* Hallowell, *Plestiodon elegans* (Boulenger), tail fragments of *Scincella vandenburghi* (Schmidt), and *Gekko japonicus* (Duméril & Bibron). It also preys on other snakes, including *Oocatochus rufodorsatus* (Cantor), eggs of *Elaphe climacophora* (Boie), and *Gloydius tsushimaensis* (Isogawa, Moriya & Mitsui), as well as young birds, rats, and even domestic animals such as a guinea pig (Pope 1935; Morikawa and Inoue 2025; TVN, pers. obs.). Females lay clutches of more than ten eggs (Pope 1935; Dieckmann et al. 2010).

#### ﻿ Lycodon walli

Taxon classificationAnimalia

(Stejneger, 1907) stat. nov.

EA377AFE-3463-51E3-8BC6-707390ED4F28

**Figure 4.**
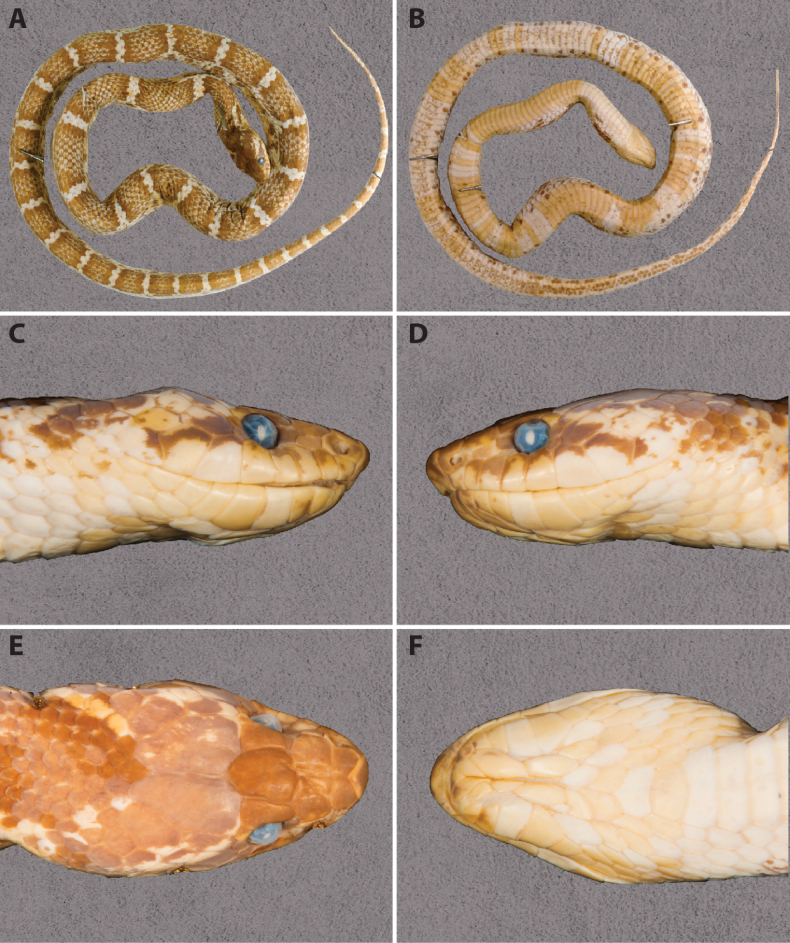
*Lycodon walli* stat. nov. in preservative – Specimen USNM 34007 (holotype, adult male): general dorsal view (A); general ventral view (B); lateral view of the head, right side (C); lateral view of the head, left side (D); dorsal view of the head (E); ventral view of the head (F). Photographs by T. Hsu (USNM).

Table 2, Fig. 4, Suppl. material 1: table S1, figs S12, S13

Dinodon rufozonatus walli Stejneger 1907: 358. Holotype: USNM [National Museum of Natural History, Smithsonian Institution, Washington, USA] 34007 was collected in June 1899 by A. Owston. Type locality: Ishigaki Island, Yaeyama Group, Ryukyu Islands, Japan.

##### Material examined.

Three adult males were examined; see in Suppl. material 1: table S1.

##### Referred material.

A total of 37 specimens were used for reference (20 males and 17 females) and were reported by Stejneger (1907), Takara (1962), and Maki (1931); see Suppl. material 1: table S1.

##### Diagnosis.

Large-sized species, maximum snout-vent length up to 922 mm; loreal not contacting the eye; dorsal scale rows 17 (19)–17–15; all smooth at midbody; 164–198 ventrals; 71–90 subcaudals, paired; cloacal plate undivided; 8 supralabials with 3–5 touching the eye; 1 preocular, 2 postoculars; temporals 2+3; dorsum chocolate colour with dorsal crossbands grey-brown or dirty cream, wide, separate ground colour into ellipse-shaped patches, 39–51 crossbands on body and tail on tail; head grey-brown the plates conspicuously margined with pale brown; venter cream or pale yellow, no banded (based on Stejneger 1907; Maki 1931; Takara 1962; this study).

##### Description of the holotype

(**Fig. 4)**: The body is robust and slightly laterally compressed. The tail is relatively long, thin, and tapering. The head is elongated, longer than wide, and moderately flattened, with a distinct separation from the neck. The snout is elongated, flattened, and slightly projects beyond the lower jaw. The nostrils are relatively large, positioned dorsolaterally, and rounded in shape. The eyes are relatively large, with vertical pupils.

***Body size*.**SVL 600 mm; TaL 190 mm; ratio TaL/TL 0.241.

***Body scalation*.** Dorsal scale rows 17–17–15, all smooth; scales of the vertebral row not enlarged; no apical pit detected; 190 ventrals; 87 subcaudals, all paired; cloacal plate undivided.

***Head scalation*.** Rostral heptagonal, wider than high, slightly visible from above; nasal single, elongated; nasal surrounded by the first two supralabials, rostral, internasal, and prefrontal; internasals two, curved, slightly wider than longer, in contact with rostral anteriorly, nasal, and prefrontal; prefrontals two, large, subrectangular, prefrontal length slightly shorter than frontal length; prefrontals in contact with internasals, nasals, preoculars, and frontal; frontal rather small, pentagonal, tapering posteriorly, shorter than the distance from tip of snout to frontal; parietals longer than wide, in contact approximately the length of the frontal; 1/1 supraocular, distinctly wider than high, in contact with prefrontal; 1/1 loreal, not contacting with the eye; 1/1 preocular, slightly large, higher than wide, in broad contact with prefrontal; subocular absent; 2/2 postoculars; 2+3 temporals; 8/8 supralabials, first and second in contact with nasal, second and third in contact with loreal, third and fourth in contact with eye, sixth largest; infralabials 10/10, first pair in broad contact with each other, first to fifth in contact with anterior pair of chin shields; posterior chin shields equal anterior ones, separated from each other by a small pair of scales.

***Colouration in preservative***: The dorsal surface is chocolate-coloured, with 25 narrow grey-brown or dirty cream crossbands on the body and 18 on the tail. Each pale crossband is approximately one dorsal scale wide, interconnecting to divide the ground colour into elliptical patches. The ventral surface of the body is predominantly cream, but fine stippling and mottling are present, increasing in density and contrast posteriorly, especially on the tail. The head is black, with a distinct inverted V-shaped marking on the nape. Pale stripes extend downward from the top of the temporal scales to the last supralabial scale.

##### General description and variation

(see Table 2; Suppl. material 1: table S1, figs S12, S13). Morphology variation based on three examined specimens as well as data morphology of 14 specimens was reported by Stejneger (1907), Maki (1931), and Takara (1962).

The longest known specimen is 1,165 mm long (adult male; SVL 922 mm, TaL 243 mm, KUZ 62999). The longest known female is 960+ mm long (SVL 840 mm, TaL 120+ mm (tail incomplete); Sci. Coll. Kyoto e). Body elongated; head distinct from neck, markedly flattened; eye medium; pupil vertically oval; rostral triangular, broader than high, clearly visible from above; internasals as broad as long, approximately half the length of the prefrontal; prefrontal shorter than frontal; frontal hexagonal; parietals large, longer than wide; nasal divided; one loreal, nearly rectangular, narrowing posteriorly, protruding somewhat beneath the preocular, not entering the eye and internasals; one preocular; two postoculars; two anterior temporals; three posterior temporals; eight (7) supralabials, 1^st^ and 2^nd^SL in contact with the nasal, 2^nd^ and 3^rd^SL in contact with the loreal, 3^rd^–5^th^SL entering orbit, 6^th^ and 7^th^SL largest; ten infralabials; first pair in contact with each other, 1^st^–5^th^IL in contact with anterior chin shields, 5^th^ and 6^th^IL largest; 17 or 19 dorsal scale rows at the head, 17 dorsal scale rows at midbody, 15 dorsal scale rows at the vent, the upper dorsal and vertebral scale rows entirely smooth; ventrals 164–198 (187.95 ± 6.21, *n* = 40), without sexual dimorphism, vertebral scale slightly enlarged, distinctly angulate laterally; cloacal plate undivided; subcaudals 71–90 (82.11 ± 4.37, *n* = 37), without sexual dimorphism; relative tail length 0.196–0.288 (0.217 ± 0.016, *n* = 33), without sexual dimorphism.

##### Colouration.

The dorsal surface of the body and tail is chocolate or black-grey, with 25–34 narrow grey-brown or dirty cream crossbands on the body and 15–22 on the tail. Each pale crossband is ~1.5–2.5 dorsal scales wide, interconnecting to divide the ground colour into elliptical patches. The ventral surface of the body is predominantly cream, but fine stippling and mottling are present, increasing in density and contrast posteriorly, especially on the tail. The head is brownish-grey with a distinct, inverted V-shaped marking on the nape. Pale pinkish or cream-coloured oblique stripes extend from the upper temporal region downward to the posterior margin of the last supralabial.

##### Etymology.

According to Stejneger (1907), the subspecies is named for Captain Frank Wall (1868–1950), of the Indian Medical Service, author of “A prodromus of the snakes hitherto recorded from China, Japan, and the Loo Choo Islands”, as well as many papers on Indian snakes. We recommend the following common names for this species: Sakishima Wolf Snake (in English); Sakishima Grosszahnnatter (in German); サキシママダラ “Sakishimamadara” (in Japan); Окинавский краснопоясный волкозуб “Okinavskiy krasnpoyasnyi volkozub” (in Russian).

##### Comparison.

*Lycodon walli* stat. nov. differs from *L. rufozonatus* sensu stricto by the following characteristics: smaller body size in both sexes (maximum SVL 922 mm in males, 840 mm in females vs 1,122 mm in males, 1145 mm in females); fewer crossbands on the body and tail (BB+TB 39–51, mean 45.40 vs 60–106, mean 79.06); slightly lower number of ventral scales in both sexes (VEN 164–198, mean 187.95 vs 186–216, mean 199.60); differences in colouration: dorsum blackish-grey or chocolate-brown with grey-brown or dirty cream crossbands vs black dorsum with pinkish or reddish-brown crossbands in *L. rufozonatus*.

##### Distribution

(Fig. 1). This species is endemic to the southern Ryukyu Islands, Japan. It has been recorded on Miyako Island and in the Yaeyama Islands, including Ishigaki Island and Iriomote Island (Maki 1931; Takara 1962; Goris and Maeda 2004; this study).

##### Natural history notes.

In the southern Ryukyus, where the species *Lycodon walli* stat. nov. occurs, it is uncommon in the Miyakojima Islands with a decreasing population, whereas it is common and stable in the Yaeyama Islands (Ota 2014). *Lycodon walli* stat. nov. is an oviparous species, with mating occurring in April and clutch sizes of 6–7 eggs laid in June or July. It is nocturnal and inhabits a wide range of habitats. Its diet includes other snakes, lizards (e.g., *Plestiodon barbouri* (Van Denburgh)), frogs (e.g., *Bufo miyakonis* Okada), and turtles (e.g., *Mauremys mutica kami* Yasukawa, Ota & Iverson). Based on observations in captivity, it is also likely to prey on rodents and small birds. On Nakanogan Island, it has been recorded consuming the eggs and chicks of seabirds. This species is terrestrial, occurring at elevations of ca 10–300 m asl, and is sympatric with *Lycodon multifasciatus* Maki and *Protobothrops elegans* (Gray) (Li et al. 2017; Goris and Maeda 2004; Takeuchi 2019; TVN, pers. obs.).

#### ﻿ Lycodon duytan sp. nov.

Taxon classificationAnimalia

8E274C87-04A1-55A2-9C9B-28F1A2D8B096

https://zoobank.org/EAC10F3B-2641-4CC8-9EEC-5AE9E81E8A18

**Figure 5.**
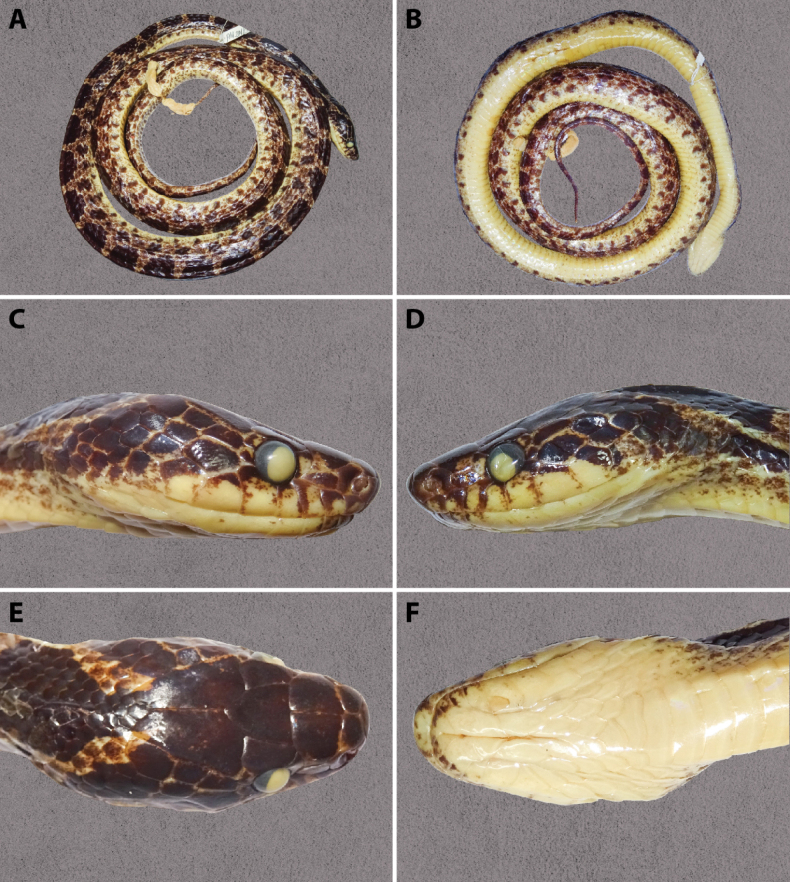
*Lycodon duytan* sp. nov. in preservative – Specimen DTU 540 (holotype, adult male): general dorsal view (A); general ventral view (B); lateral view of the head, right side (C); lateral view of the head, left side (D); dorsal view of the head (E); ventral view of the head (F). Photos by TVN.

Table 2, Fig. 5; Suppl. material 1: table S1, fig. S14

##### Type material.

***Holotype***: • DTU [Duy Tan University, Da Nang, Vietnam] 540 (adult male) collected on 19 April 2018 by TVN in Khe Choang Areas within Pu Mat National Park, Chau Khe Commune, Con Cuong District, Nghe An Province, Vietnam (ca 18.964811°N, 104.651873°E; altitude 550 m asl). ***Paratypes*** (*n* = 4): • DTU 541 (adult female), same information with holotype; DTU 542 (adult female) in Vu Quang NP, Huong Quang Commune, Vu Quang District, Ha Tinh Province, Vietnam (ca 18.264117°N, 105.435481°E; altitude 540 m asl), collected in March 2019 by TVN and T.C. Thai ; • DTU 543–544 (adult females), collected from Mac Area within Cuc Phuong NP, Cuc Phuong Commune, Nho Quan District, Ninh Binh Province, Vietnam (ca 20.268796°N, 105.689175°E; altitude 215 m asl), collected in June 2018 by T.N. La and TVN.

##### Referred materials

(*n* = 6). CPNP NHQ.225 (adult female) and CPNP NHQ.240 (adult female), collected from Cuc Phuong NP, Cuc Phuong Commune, Nho Quan District, Ninh Binh Province, Vietnam. CPNP NHQ.2017.18 (adult female), collected from Cuc Phuong NP, Thanh Yen Commune, Thach Thanh District, Thanh Hoa Province, Vietnam (ca 20.288652°N, 105.584416°E; altitude 150 m asl) collected on 26 August 2017 by Q.H. Nguyen. SIFASV 104 and 105 (two adult females, released), collected from Pu Mat NP, Con Cuong District, Nghe An Province, Vietnam, in May 2017 by Q.S. Nguyen. SIFASV 106 (adult female, released), collected from Nam Dong NR, Quan Hoa District, Thanh Hoa Province, Vietnam, in May 2023 by N.V. Ha and TVN.

##### Diagnosis.

A larger-sized species, with a maximum snout-vent length of up to 980 mm; loreal usually contacting the eye; dorsal scale rows 17–17–15; upper four or five and vertebral dorsal scale rows keeled; 217–230 ventrals; 80–95 subcaudals, paired; cloacal plate undivided; eight supralabials with 3–5 touching the eye; 1 preocular, 2 postoculars; temporals 2+2; dorsal crossbands narrow, separating ground colour into ellipse patches, pinkish-orange colour, 54–72 crossbands on body and tail; head black, the plates conspicuously margined with pinkish-orange; venter reddish-orange.

##### Description of the holotype

(**see Fig. 5)**: The body is robust and slightly laterally compressed. The tail is relatively long, thin, and tapering. The head is elongate, longer than wide, and moderately flattened, with a distinct separation from the neck. The snout is elongated, flattened, and projects slightly over the lower jaw. The nostrils are relatively large, positioned dorsolaterally, and round in shape. The eyes are relatively large, with vertical pupils.

***Body size*.**SVL 890 mm, TaL 223 mm; ratio TaL/TL 0.200.

***Body scalation*.** Dorsal scale rows 17–17–15, the five upper rows feebly keeled; scales of the vertebral row not enlarged; no apical pit detected; 229 ventrals; 94 subcaudals, all paired; cloacal plate undivided.

***Head scalation*.** Rostral heptagonal, wider than high, slightly visible from above; nasal single, elongated; nasal surrounded by the first two supralabials, rostral, internasal, and prefrontal; internasals two, curved, slightly wider than longer, in contact with rostral anteriorly, nasal, and prefrontal; prefrontals two, large, subrectangular, prefrontal length slightly shorter than frontal length; prefrontals in contact with internasals, nasals, preoculars, and frontal; frontal rather small, pentagonal, tapering posteriorly, shorter than the distance from tip of snout to the frontal; parietals longer than wide, in contact approximately the length of the frontal; 1/1 supraocular, distinctly wider than high, in contact with prefrontal; 1/1 loreal, not contacting with the eye; 1/1 preocular, slightly large, higher than wide, in broad contact with prefrontal; subocular absent; 2/2 postoculars; 2+2 temporals; 8/8 supralabials, first and second in contact with nasal, second and third in contact with loreal, third and fourth in contact with eye, sixth largest; infralabials 9/9, first pair in broad contact with each other, first to fifth in contact with anterior pair of chin shields; posterior chin shields equal anterior ones, separated from each other by a small pair of scales.

***Colouration in preservative***: The dorsum is blackish-brown, with 49 narrow pale-coloured crossbands on the body and 23 on the tail. Each pale-coloured body crossband is approximately one dorsal scale wide, interconnecting to divide the ground colour into elliptical patches. The ventral surface of the body is uniformly cream, while the ventral surface of the tail is heavily speckled with dark markings. The head is black, featuring a distinct inverted V-shaped marking on the nape. Pale stripes extend downward from the top of the temporal scales to the last supralabial scale.

##### Variation

**(Table 2, Suppl. material 1: table S1, fig. S14).** The type series is generally similar to the holotype in terms of body proportions and colouration, with only slight variation observed. All examined specimens of *Lycodon duytan* sp. nov. from geographically distinct localities in north-central Vietnam (Nghe An, Ha Tinh, Thanh Hoa, and Ninh Binh provinces) exhibit consistent diagnostic features, including the presence of four or five keeled midbody dorsal scale rows, a high number of ventral and subcaudal scales (VEN 217–230; SC 80–95), and narrow, pinkish-orange crossbands on the body and tail. Intraspecific variation was minimal. The number of total crossbands (BB+TB) ranged from 54 to 72. The loreal scale was in slight contact with the eye in seven of ten specimens, likely reflecting minor individual variation or asymmetry. No notable variation was observed in body proportions or overall colour pattern across the examined specimens. The dorsal surface is blackish, with 38–49 narrow pinkish-orange crossbands on the body and 14–24 on the tail; these bands interconnect, dividing the ground colour into elliptical patches. The ventral surface of the body is uniformly pale pink, while the tail’s underside exhibits variable dark speckling. The head is black, with a distinct inverted V-shaped marking on the nape and pale stripes extending from the top of the temporal region to the last supralabial. The overall morphological uniformity across multiple localities and habitats supports the recognition of *Lycodon duytan* sp. nov. as a distinct and geographically cohesive lineage.

##### Etymology.

The species name is derived from the Duy Tan Modernisation Movement (Phong trào Duy Tân) of 1906–1908 in Vietnam, a significant historical reform movement for the modernisation of the country and the society. The specific epithet also acknowledges Duy Tan University, the institution affiliated with the first author, which provided support for this research. We recommend the following common names for this species: Duy Tan Wolf Snake (in English); Duy Tan Grosszahnnatter (in German); Rắn khuyết Duy Tân (in Vietnamese); Южный краснопоясный волкозуб “Yuzhnyi krasnopoyasnyi volkozub” (in Russian).

##### Comparison.

*Lycodon duytan* sp. nov. differs from all known congeners by the unique combination of the following characters: a large body size (maximum SVL up to 980 mm); 4–5 keeled dorsal scale rows at midbody (vs smooth or only weakly keeled in most species); a high number of ventral and subcaudal scales (VEN 217–230; SC 80–95); 54–72 narrow pinkish-orange crossbands on the body and tail, which interconnect to divide the ground colour into elliptical patches; and a uniformly reddish-pink ventral surface. These features clearly distinguish *Lycodon duytan* sp. nov. from *L. rufozonatus*, *L. walli* stat. nov., and other members of the *L. rufozonatus–paucifasciatus* species complex, including *L. anakradaya* Nguyen, Duong, Wood & Grismer, *L. cardamomensis* Daltry & Wüster, *L. paucifasciatus* Rendahl in Smith, *L. poyarkovi* Nguyen & Vogel, *L. rosozonatus* (Hu & Zhao), and *L. gibsonae* Vogel & David. These species are all medium to large-sized snakes (total length ≥ 600 mm) sharing superficially similar coloration patterns, namely red or pinkish crossbands on a dark background and an inverted V-shaped marking on the nape, but differ from *Lycodon duytan* sp. nov. in key morphological traits. Given these similarities, detailed comparisons with these eight species are provided below.

*Lycodon duytan* sp. nov. is morphologically very similar to *L. rufozonatus* but can be distinguished by the following characteristics: a higher number of ventral scales (VEN 217–230, mean 225.1 vs 186–216, mean 199.60), a greater number of subcaudal scales (SC 80–95, mean 88.11 vs 60–88, mean 74.30), and the presence of 4 or 5 keeled scale rows at midbody (vs all dorsal scales smooth).

*Lycodon duytan* sp. nov. differs from *L. anakradaya* (data from Nguyen et al. 2022), *L. cardamomensis* (data from Do et al. 2017; Nguyen and Vogel 2025), and *L. paucifasciatus* (data from Smith 1943; Nguyen and Vogel 2025) by having: pale crossbands on the dorsum and tail that link with each other and separate the ground colour of the body into ellipse-shaped patches (vs separate with each other); a higher number of BB+TB in both sexes (54–72 vs 15–26); and crossbands on the dorsum and tail that are narrow ~ 1.0–1.5 dorsal scales wide along the vertebral scale row (vs wide, ~3.0–5.0 dorsal scales).

*Lycodon duytan* sp. nov. differs from *L. poyarkovi* (data from Nguyen and Vogel 2025) by having: much bigger size in both sexes (max SVL 890 mm in males, 980 mm in females vs 536 mm in males, 675 mm in females); pale crossbands on the dorsum and tail linked with each other and separate the ground colour of the body into ellipse-shaped patches (vs separate with each other); a higher number of BB+TB in both sexes (54–72 vs 31–47); and crossbands on the dorsum and tail narrow, ~1.0–1.5 dorsal scales wide along the vertebral scale row (vs slightly wide, ~2.0–2.5 dorsal scales).

*Lycodon duytan* sp. nov. differs from *L. rosozonatus* (data from Nguyen and Vogel 2025) by having: smaller size in males (max SVL 890 mm vs 1060 mm) but a larger size in females (max SVL 980 mm vs 866 mm); a lower number of MSR in both sexes (17 vs 19); a higher number of BB+TB in both sexes (54–72 vs 39–42); and crossbands on the dorsum and tail, ~1.0–1.5 dorsal scales wide along the vertebral scale row (vs slightly wide, ~2.0–2.5 dorsal scales).

*Lycodon duytan* sp. nov. differs from *L. gibsonae* (data from Vogel and David 2019) by having: pale crossbands on the dorsum and tail that link with each other and a separate ground colour of the body into ellipse-shaped patches (vs separate with each other); a higher number of BB+TB in males (72 vs 25–27); and crossbands on the dorsum and tail ~1–1.5 dorsal scales wide along the vertebral scale row (vs slightly wide, ~4.5–6.0 dorsal scales).

Lastly, *Lycodon duytan* sp. nov. differs from *L. walli* stat. nov. in several morphological characteristics, including: dorsum colouration (blackish with pinkish-orange crossbands vs black-grey or chocolate with grey-brown or dirty cream crossbands); a higher number of ventral scales (VEN 217–230, mean 225.09 vs 164–198, mean 187.95); a higher number of BB+TB in both sexes (54–72 vs 39–51); and the presence of 4–5 keeled dorsal scale rows at midbody (vs all dorsal scales smooth). Geographically, *Lycodon duytan* sp. nov. is widely separated from *L. walli* stat. nov., which is considered endemic to the southern Ryukyu Islands, Japan. Furthermore, the distribution range of *L. rufozonatus* lies between these two species, further reinforcing their geographic isolation.

##### Distribution

(Fig. 1). Currently, *Lycodon duytan* sp. nov. is known from Cuc Phuong NP (Nho Quan District, Ninh Binh Province, and Thach Thanh District, Thanh Hoa Province); Nam Dong NR (Thanh Hoa Province); Pu Mat NP (Nghe An Province); and Vu Quang NP (Ha Tinh Province) in north-central Vietnam. Additionally, its presence is anticipated in Pu Luong and Pu Hu NRs (Thanh Hoa Province) and Pu Hoat NR (Nghe An Province) in north-central Vietnam.

##### Natural history notes.

This species is nocturnal and terrestrial, as observed during our field surveys. All observed specimens were found crawling on the ground or on limestone rocks near small to medium-sized streams. Despite its relatively wide distribution, *Lycodon duytan* sp. nov. appears to be rare within its habitat. In Cuc Phuong NP, Ninh Binh Province, *Lycodon duytan* sp. nov. was recorded in sympatry with *L. futsingensis* (Pope), *L. meridionalis*, and *L. ruhstrati abditus* Vogel, David, Pauwels, Sumontha, Norval, Hendrix, Vu & Ziegler within secondary karst forests. In Pu Mat NP, Nghe An Province, this species was found in sympatry with *L. futsingensis* and *L. neomaculatus* Nguyen, Lee, Pauwels, Kennedy-Gold, Poyarkov, David & Vogel, in an evergreen forest habitat. In Nam Dong NR, Thanh Hoa Province, and Vu Quang NP, Ha Tinh Province, *Lycodon duytan* sp. nov. was recorded in sympatry with *L. futsingensis* and *L. ruhstrati abditus* in secondary forest.

##### Conservation status.

*Lycodon duytan* sp. nov. has a relatively large distribution range, occurring across at least four protected areas, including three national parks and one nature reserve in northern and central Vietnam, which afford it a certain degree of conservation protection. The estimated extent of occurrence (EOO) is ca 17,307 km^2^. The primary threats to this species include habitat loss and degradation, as well as potential illegal collection due to its distinctive colouration (TVN, pers. obs.). Based on these factors, *Lycodon duytan* sp. nov. is classified as a species of Least Concern (LC) according to the IUCN Red List categories (IUCN Standards and Petitions Committee 2024).

## ﻿Discussion

We evaluated the species diversity within the *Lycodon rufozonatus* complex, clarified its actual geographic distribution, assessed the validity of nominal taxa so far hidden within the complex, and provided an identification key based on extensive geographical sampling across its range. Additionally, we re-examined all available names and their respective type specimens. Based on our findings, we consider *Lycodon rufozonatus* sensu stricto to be distributed in East Asia, including China (including Taiwan), southern Russia, the Korean Peninsula, southern Japan, and northeastern Vietnam. *Dinodon rufozonatus walli* is here elevated to full species status as *Lycodon walli* stat. nov., based on distinct morphological differences and its isolated distribution in the Ryukyu Islands. Furthermore, we revise the populations from north-central Vietnam, previously referred to as Lycodon cf. rufozonatus, and describe them as a new species, *Lycodon duytan* sp. nov. The elevation of *Lycodon walli* stat. nov. to full species status is unsurprising, as it exhibits easily recognisable morphological differences from *L. rufozonatus*. Several recent studies have demonstrated that certain endemic species from the Miyako and Yaeyama islands in the southern Ryukyus, Japan, such as amphibians (*Bufo miyakonis*) and reptiles (*Takydromus toyamai* Takeda & Ota, *Calamaria pfefferi* (Stejneger), have their closest relatives or subspecies distributed in mainland China and Taiwan and are recognised at the species level (see Watanabe et al. 2023). Since we were unable to obtain molecular data for *Lycodon walli* stat. nov., further molecular sampling is required to assess its genetic relationships with other species of *Lycodon*, particularly within the *Lycodon rufozonatus* species complex. Notably, *Lycodon rufozonatus* exhibits colour polymorphism. The Taiwanese population is distinguished by slightly wider crossbands along the vertebral scale row compared to other populations (1.5–2.5 dorsal scales wide vs 1.0–1.5 dorsal scales wide). Colour polymorphism has also been reported in other *Lycodon* species, such as *Lycodon bicolour* (Nikolsky), *L. gongshan* Vogel & Luo, *L. ruhstrati* (Fischer), and *L. liuchengchaoi* Zhang, Jiang, Vogel & Rao (see Amarasinghe et al. 2023; Nguyen et al. 2025a, b).

The taxonomy and distribution of *Lycodon rufozonatus* in Vietnam have been historically ambiguous. This species was previously recorded in northern and central Vietnam, including Tuyen Quang Province (Na Hang NR), Vinh Phuc Province (Tam Dao NP), Ha Tinh Province (Vu Quang NP, Ky Anh District), Quang Binh Province (Phong Nha-Ke Bang NP), and Quang Tri Province (Bac Huong Hoa NR) (Ziegler 2002; Orlov and Ryabov 2004; Nguyen et al. 2009). In the present study, *L. rufozonatus* is confirmed to occur in northeastern Vietnam, specifically in Bac Giang, Tuyen Quang, and Vinh Phuc provinces. The previous record from Vu Quang NP, Ha Tinh Province, is now reassigned to *Lycodon duytan* sp. nov. The specimen from Ky Anh District, Ha Tinh Province, originally obtained through the local animal trade between Ky Thuong and Chin Xai communes, was an adult female (collection number TZ. 98/49) with the following morphological characteristics: TL = 920 mm, SL = 8/8, IL = 9/10, PrO = 1/1, PoO = 2/2, AT = 2/2, PT = 3/3, VEN = 222, SC = 93, MSR = 17, BB = 32, TB = 31 (Ziegler, 2002). This specimen is most likely attributable to *Lycodon poyarkovi*. Additionally, populations previously reported from Quang Binh Province (Phong Nha-Ke Bang NP) and Quang Tri Province (Bac Huong Hoa NR) have been re-examined and identified as misidentified *Lycodon poyarkovi* (Nguyen and Vogel 2025). *Lycodon duytan* sp. nov. appears to be restricted to north-central Vietnam, whereas its sister species, *L. rufozonatus*, is distributed in northeastern Vietnam and southern China. These two species are geographically separated by the Red River (*Sông Hồng*), which serves as a significant biogeographic barrier for amphibians and reptiles (Bain and Hurley 2011; Poyarkov et al. 2021, 2023). Given that the Red River is the largest river in northern Vietnam, its valley likely functions as a physical/ biogeographic barrier that restricts gene flow between *Lycodon duytan* sp. nov. and *L. rufozonatus* populations. The discovery of *Lycodon duytan* sp. nov. provides new insights into the ecological plasticity of the *Lycodon rufozonatus* species complex in north-central Vietnam. Unlike many congeners that are typically restricted to either lowland or montane forests, *Lycodon duytan* sp. nov. has been documented in both karst (limestone) and non-karst (soil-based) forest habitats, ranging in elevation from approximately 150 to 550 m asl. This ecological breadth suggests a relatively high degree of adaptability and may explain its wide but previously overlooked distribution. Sympatric assemblages at several localities include *L. futsingensis*, *L. meridionalis*, *L. ruhstrati abditus*, and *L. neomaculatus*, highlighting the ecological complexity and species interactions within these forest ecosystems.

Due to the morphological similarities between species of the *Lycodon rufozonatus* group and the *L. paucifasciatus* group, we provide an identification key herein. Notably, *Lycodon paucifasciatus* is not distinguishable at the species level from its two sympatric species, *L. anakradaya* and *L. cardamomensis* (see discussion in Nguyen and Vogel 2025).

### ﻿Key to the species of the *Lycodon rufozonatus*-*paucifasciatus* species complex

**Table:** 

1	Pale crossbands on the dorsal body and tail linked with each other, forming elliptical patches of ground colour	**2**
–	Pale crossbands on the dorsal body and tail separated from each other	**5**
2	Usually < 50 crossbands on the body and tail	**3**
–	Usually > 50 crossbands on the body and tail	**4**
3	17 dorsal scale rows at midbody; dorsum black-grey or chocolate coloured with grey-brown or dirty cream crossbands; endemic to the southern Ryukyu Islands, Japan	***Lycodon walli* stat. nov.**
–	19 dorsal scale rows at midbody; dorsum blackish with pink or reddish-brown crossbands; endemic to Hainan Island, China	***Lycodon rosozonatus***
4	Total ventral + subcaudal scales ≥ 299; midbody scales slightly keeled in 4 or 5 rows	***Lycodon duytan* sp. nov.**
–	Total ventral + subcaudal scales ≤ 297; all midbody scales smooth	***Lycodon rufozonatus***
5	Usually < 30 crossbands on the body and tail	**6**
–	Usually > 30 crossbands on the body and tail	***Lycodon poyarkovi***
6	Loreal scale not touching the eye; usually < 16 crossbands on the body	***Lycodon paucifasciatus* complex species (*L. paucifasciatus*, *L. anakradaya*, and *L. cardamomensis*)**
–	Loreal scale touching the eye; usually more than 16 crossbands on the body	***Lycodon gibsonae***

## Supplementary Material

XML Treatment for Lycodon rufozonatus

XML Treatment for Lycodon walli

XML Treatment for Lycodon duytan

## ﻿Additional information

### Use of AI

No use of AI was reported.

### Author ORCIDs

Tan Van Nguyen https://orcid.org/0000-0001-5413-968X

Nikolay A. Poyarkov https://orcid.org/0000-0002-7576-2283

Gernot Vogel https://orcid.org/0000-0002-4542-518X

### Data availability

All of the data that support the findings of this study are available in the main text or Supplementary Information.

## Supplementary materials

Supplementary material 1 Additional information https://doi.org/10.3897/zookeys.1251.157817.suppl1 Data type pdf Explanation note **fig. S1.** Bayesian tree of the *Lycodon* spp. based on the partial sequences of Cyt *b* gene. **fig. S2.** Maximum likelihood tree of the *Lycodon* spp. based on the partial sequences of Cyt *b* gene. **fig. S3.**PCA scatter plots of *Lycodon duytan* sp. nov, *L. rufozonatus*, and *L. walli* stat. nov. **fig. S4.***Lycodon rufozonatus* in preservative – Specimen ANSP 3477 (syntype of *Coronella striata*). **fig. S5.***Lycodon rufozonatus* in preservative – Specimen NMBE 1016377 (topotype of *Coronella striata*). **fig. S6.***Lycodon rufozonatus* in preservative – Specimen SMF 18045 (holotype of Dinodon rufozonatus var. formosana). **fig. S7.***Lycodon rufozonatus* in preservative – specimen FMNH 7529 (paratype of *Dinodon rufozonatum williamsi*). **fig. S8.***Lycodon rufozonatus* (all syntypes of *Dinodon rufozonatum yunnanense*) in preservative. **fig. S9.***Lycodon rufozonatus* in preservative. **fig. S11.***Lycodon rufozonatus* in life in South Korea and Taiwan, China. **fig. S12.***Lycodon walli* stat. nov. in preservative – Specimen KUZ R62233. **fig. S13.***Lycodon walli* stat. nov. in life in Japan. **fig. S14.***Lycodon duytan* sp. nov. in life – specimen DTU 541. **table S1.** Measurements and scale counts of *Lycodon rufozonatus* species complex. **table S2.** Summary statistics of the principal components analysis (PCA). **table S3.** DNA sequences, voucher specimens, GenBank accession numbers of *Lycodon* and outgroup taxa used in this study. **table S4.** The species delimitation results of Bayesian PTP (bPTP) and ASAP with Jukes-Cantor distance (JC69) and Kimura (K80) ts/tv 2.0. **table S5.** Acronyms of museums and other natural history collections mentioned in this study. **table S6.** Uncorrected (“p”) distance matrix showing percentage pairwise genetic divergence (cytochrome b) between *Lycodon duytan* sp. nov. and closely related species. **table S7.** List of localities of the *Lycodon duytan* sp. nov., *L. walli* stat. nov., *L. rufozonatus*, and *L. rosozonatus* appearing on Fig. 1. https://binary.pensoft.net/file/1416276This dataset is made available under the Open Database License (http://opendatacommons.org/licenses/odbl/1.0/). The Open Database License (ODbL) is a license agreement intended to allow users to freely share, modify, and use this Dataset while maintaining this same freedom for others, provided that the original source and author(s) are credited.Tan Van Nguyen, Nikolay A. Poyarkov, Gernot Vogel
