# Supplementary material for: ﻿Taxonomic reassessment of the Lycodon rufozonatus species complex (Serpentes, Colubridae), with re-evaluation of Dinodon rufozonatum walli, and description of a new species from north-central Vietnam
Source: Zookeys. 2025 Sep 15;1251:293–322. doi: 10.3897/zookeys.1251.157817 (PMC12455217; doi:10.3897/zookeys.1251.157817)
Supplement: Supplementary material 1 — Additional information [file zookeys-1251-293_article-157817__-s001.pdf]

Taxonomic reassessment of the *Lycodon rufozonatus* species complex (Serpentes, Colubridae), with re-evaluation of *Dinodon rufozonatum walli*, and description of a new species from North-central Vietnam

Tan Van Nguyen, Nikolay A. Poyarkov, Gernot Vogel

Authors' last names: Nguyen, Poyarkov, Vogel

Corresponding authors: Tan Van Nguyen (tan.sifasv@gmail.com).

Higher taxa names: Reptilia, Squamata, *Lycodon*

Number of new taxa described in this paper: 1

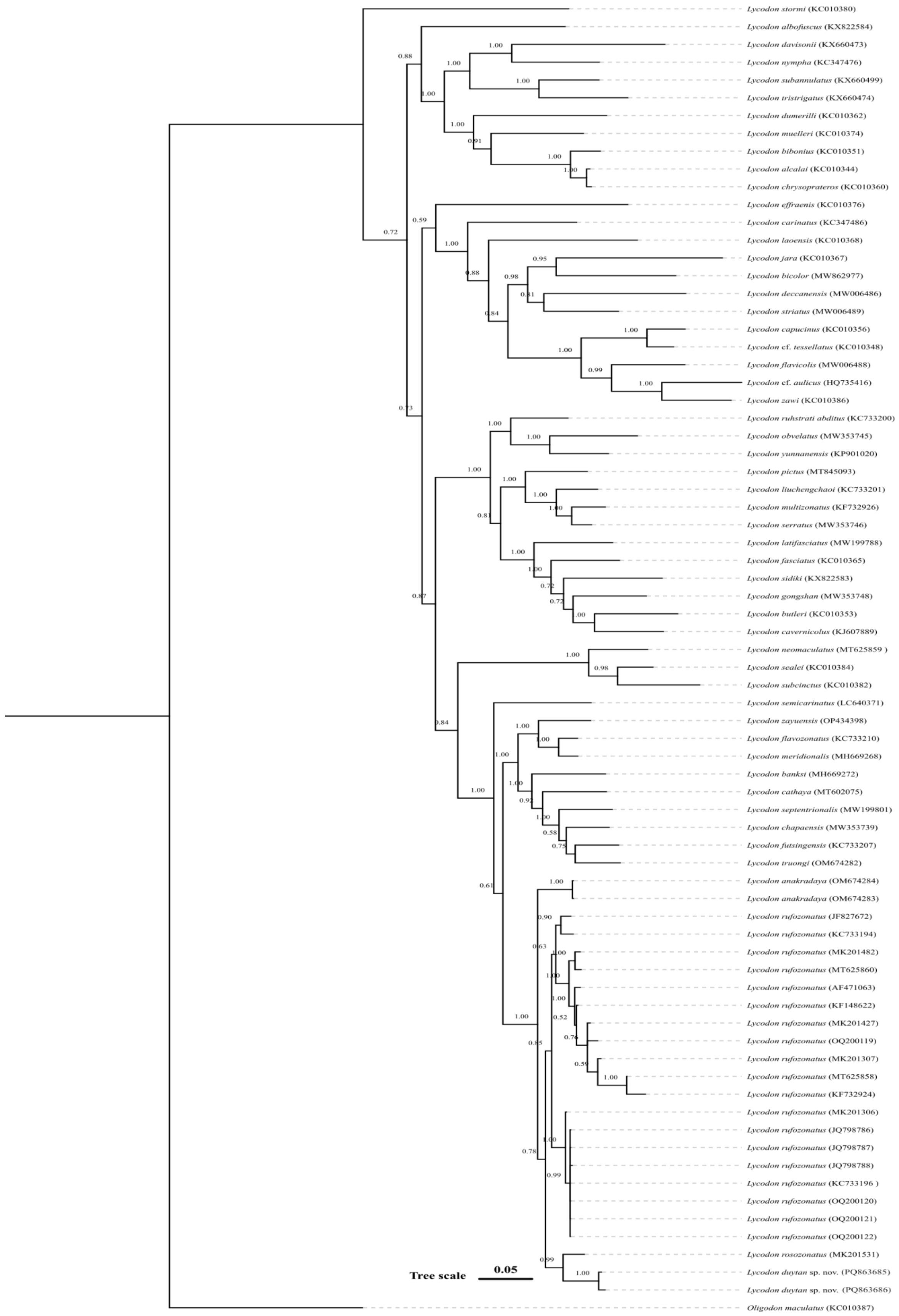

Suppl. Figure S1. Bayesian tree of the *Lycodon* spp. based on the partial sequences of Cyt *b* gene. Values of Bayesian posterior probabilities are indicated at nodes.

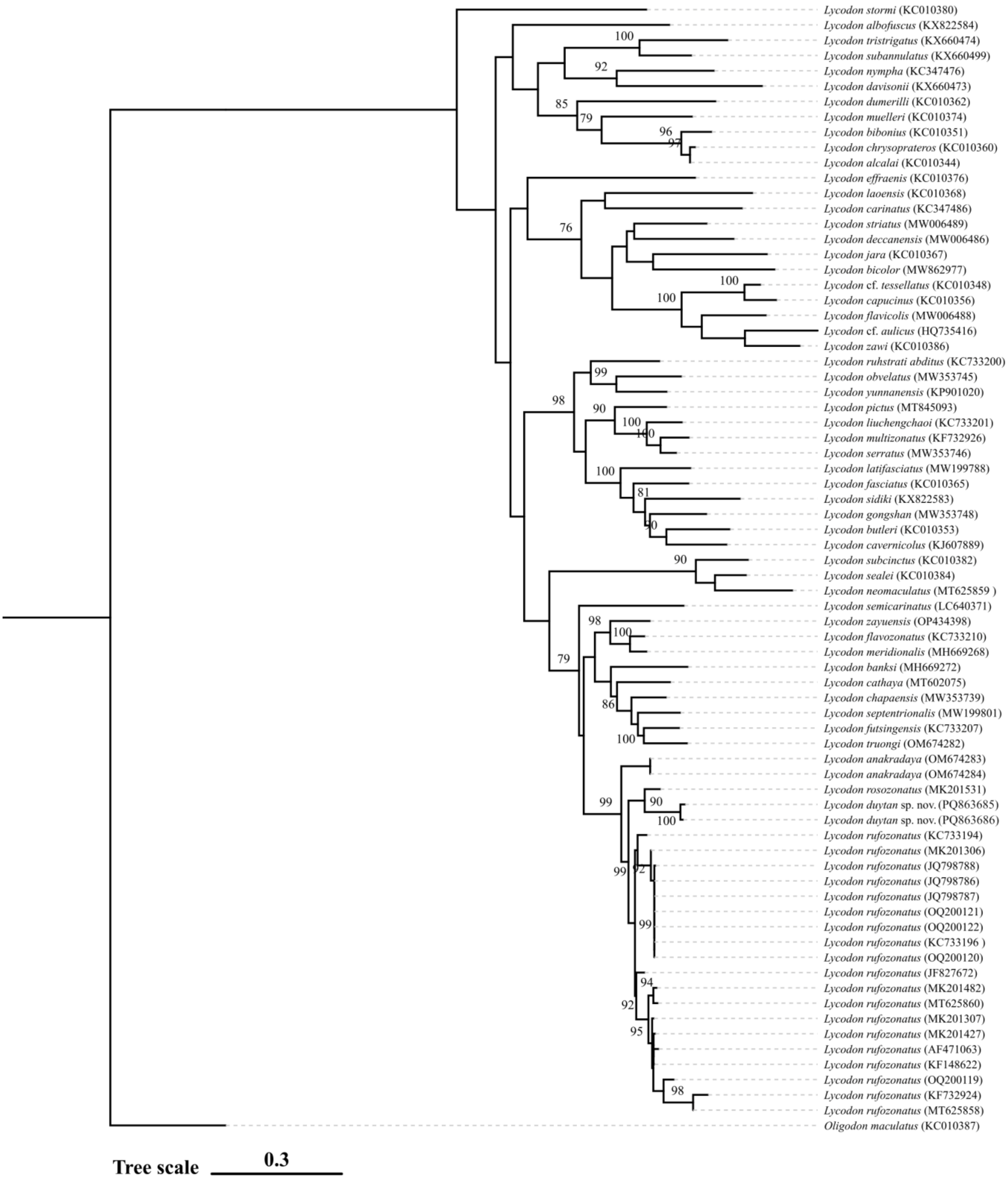

Suppl. Figure S2. Maximum likelihood tree of the *Lycodon* spp. based on the partial sequences of Cyt *b* gene. Values of bootstraps are indicated at nodes.

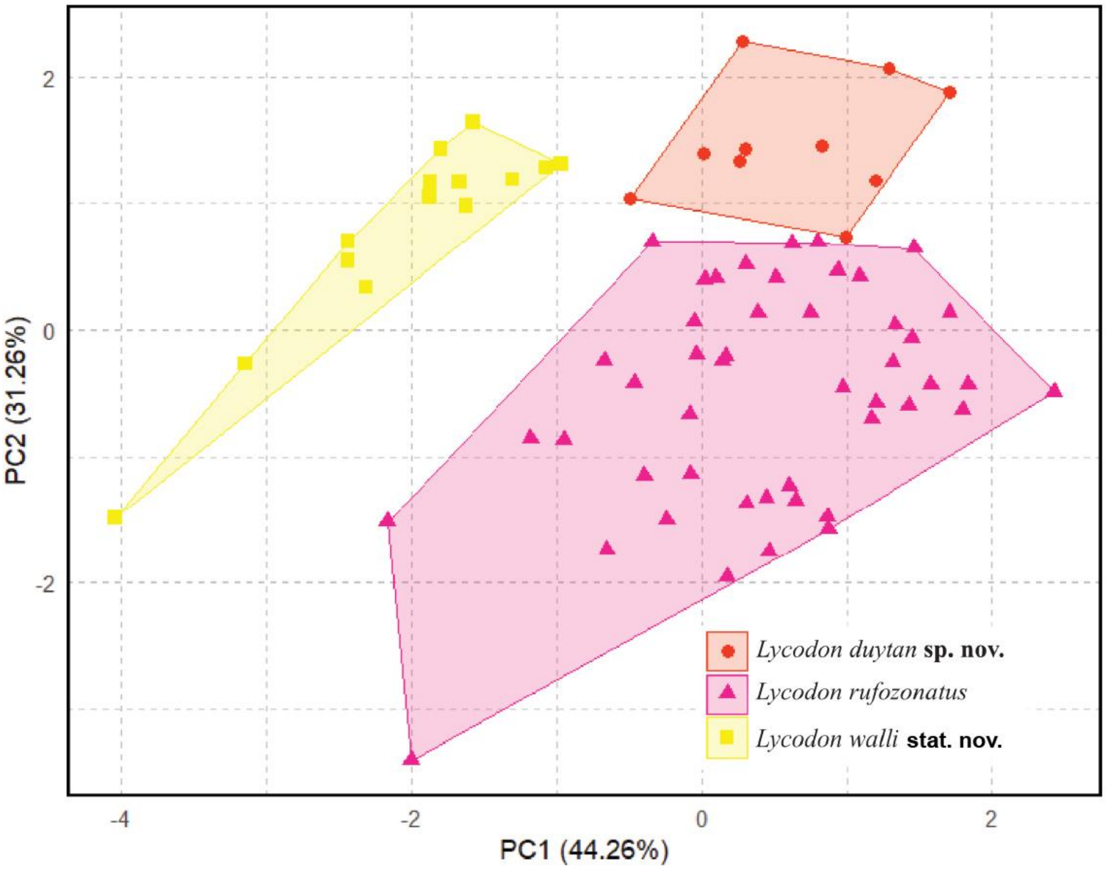

Suppl. Figure S3. PCA scatter plots of *Lycodon duytan* **sp**. **nov**, *L*. *rufozonatus*, and *L*. *walli* **stat. nov.** The shape and colour indicated signify different clusters of each *Lycodon* spp.

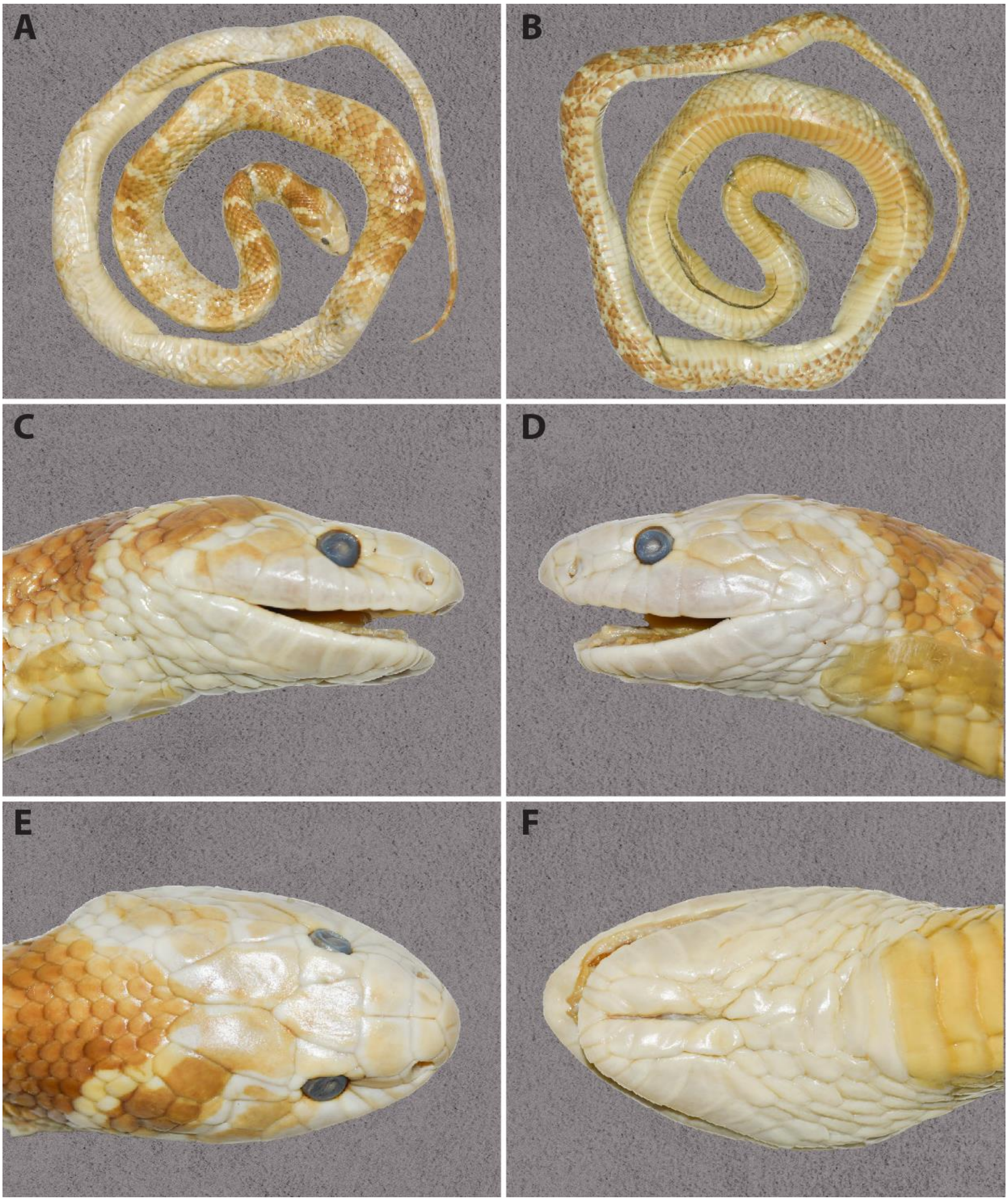

Suppl. Figure S4. *Lycodon rufozonatus* in preservative – Specimen ANSP 3477 (syntype of *Coronella striata*, adult female): general dorsal view (**A**); general ventral view (**B**); lateral view of the head, right side (**C**); lateral view of the head, left side (**D**); dorsal view of the head (**E**); ventral view of the head (**F**). Photos by N.S. Gilmore.

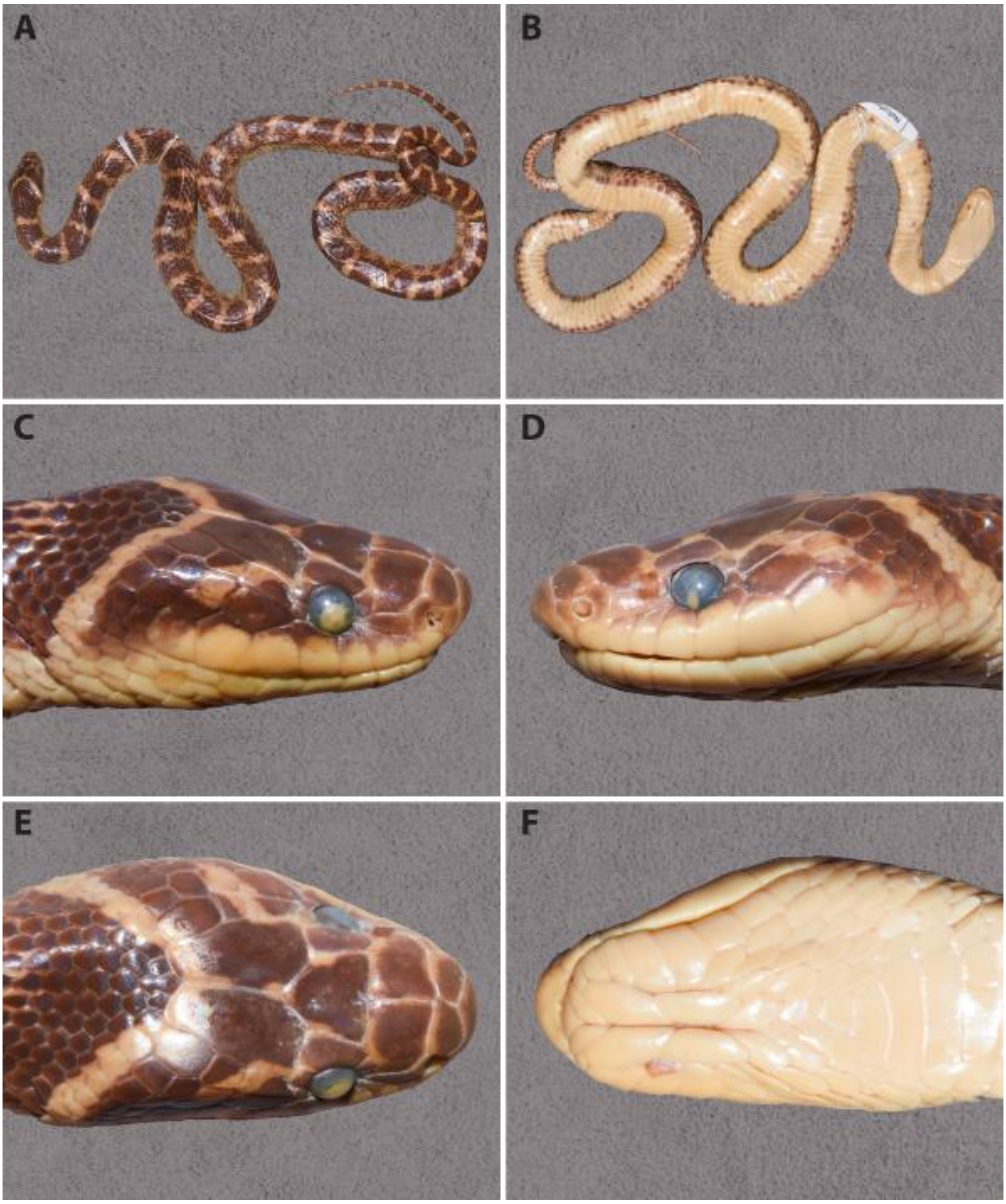

Suppl. Figure S5. *Lycodon rufozonatus* in preservative – Specimen NMBE 1016377 (topotype of *Coronella striata*, adult male): general dorsal view (**A**); general ventral view (**B**); lateral view of the head, right side (**C**); lateral view of the head, left side (**D**); dorsal view of the head (**E**); ventral view of the head (**F**). Photos by G. Vogel.

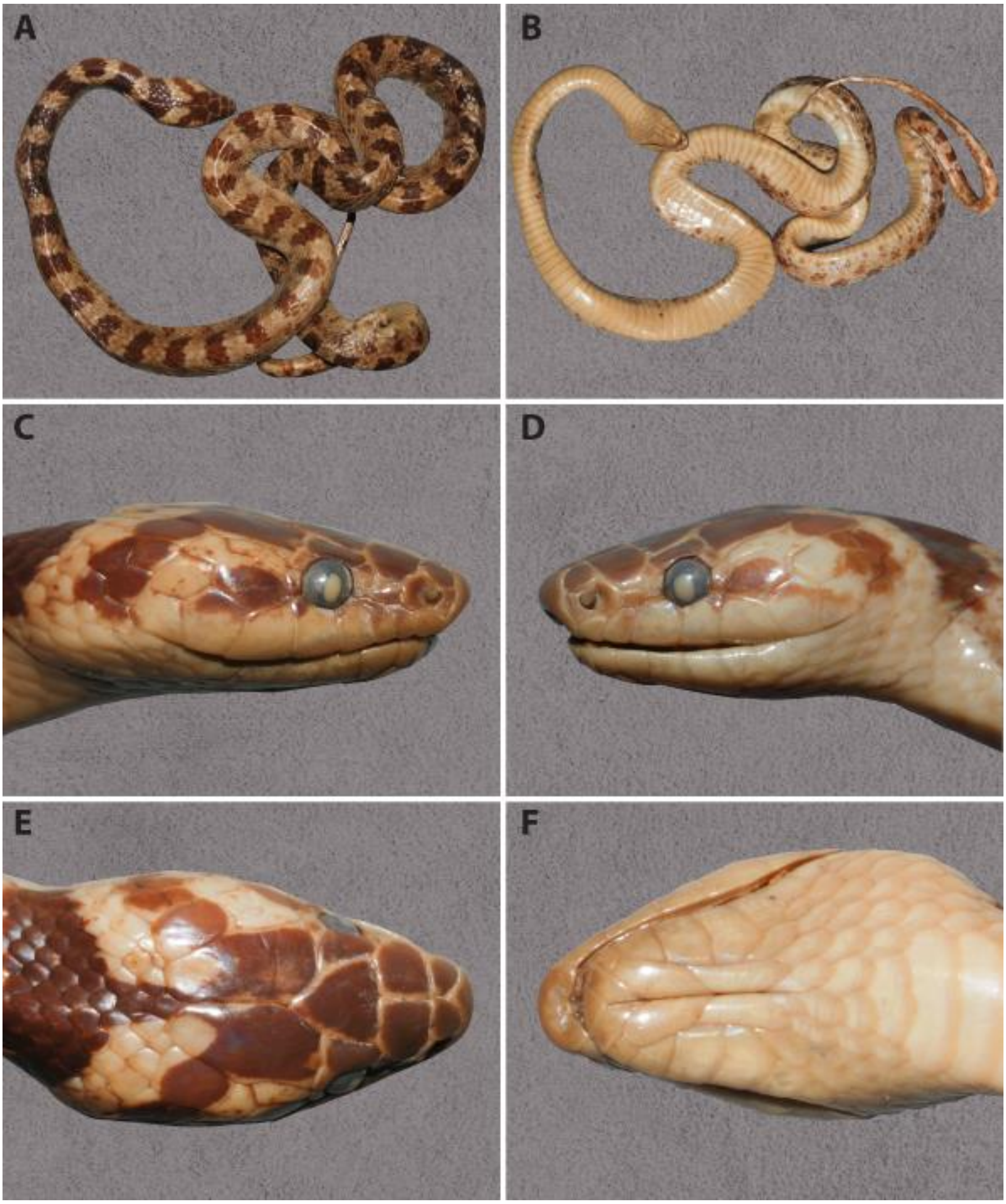

Suppl. Figure S6. *Lycodon rufozonatus* in preservative – Specimen SMF 18045 (holotype of *Dinodon rufozonatus var*. *formosana*, adult male): general dorsal view (**A**); general ventral view (**B**); lateral view of the head, right side (**C**); lateral view of the head, left side (**D**); dorsal view of the head (**E**); ventral view of the head (**F**). Photos by G. Vogel.

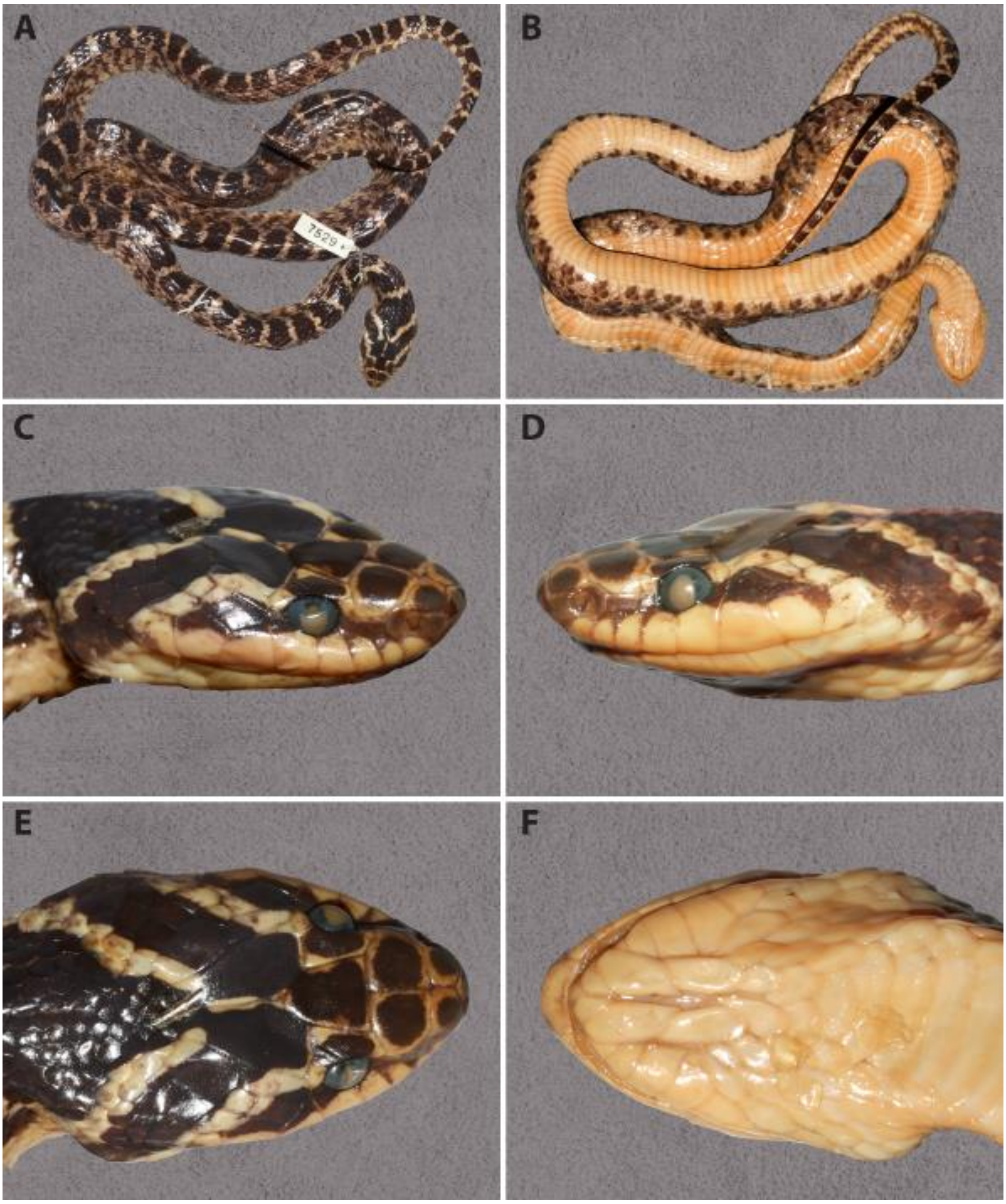

Suppl. Figure S7. *Lycodon rufozonatus* in preservative – specimen FMNH 7529 (paratype of *Dinodon rufozonatum williamsi*, adult male): general dorsal view (**A**); general ventral view (**B**); lateral view of the head, right side (**C**); lateral view of the head, left side (**D**); dorsal view of the head (**E**); ventral view of the head (**F**). Photos by G. Vogel.

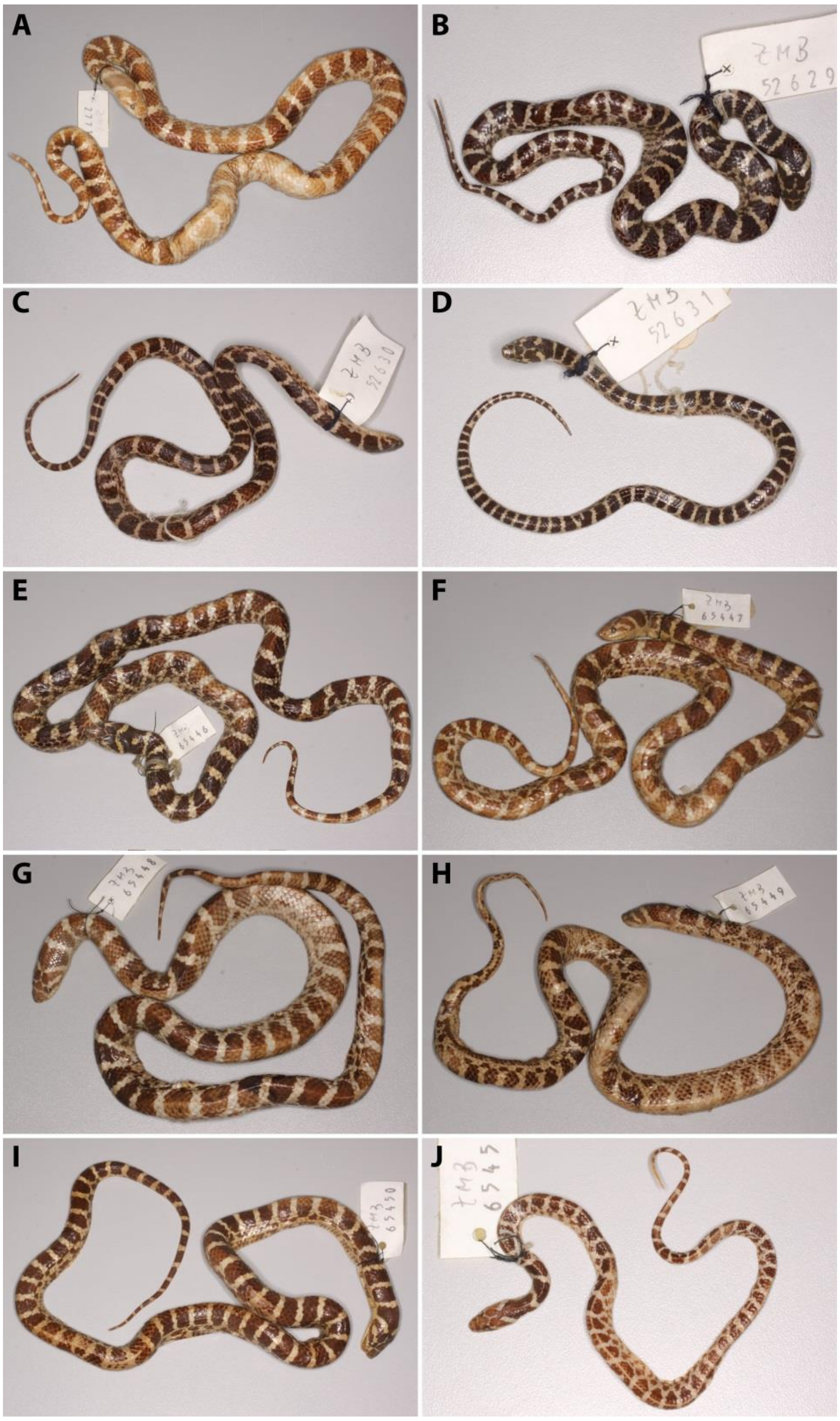

Suppl. Figure S8. *Lycodon rufozonatus* (all syntypes of *Dinodon rufozonatum yunnanense*) in preservative in general dorsal view. **A** – specimen ZMB 27711 (adult, male); **B** – specimen ZMB 52629 (subadult, male); **C** – specimen ZMB 52630 (subadult, female); **D** – ZMB 52631 (subadult, female); **E** – specimen ZMB 65446 (adult, male); **F** – specimen ZMB 65447 (adult, female); **G** – specimen ZMB 65448 (adult, male); **H** – specimen ZMB 65449 (subadult, female); **I** – specimen ZMB 65450 (subadult, female); **J** – specimen ZMB 65451 (subadult, female). Photos by F. Tillack.

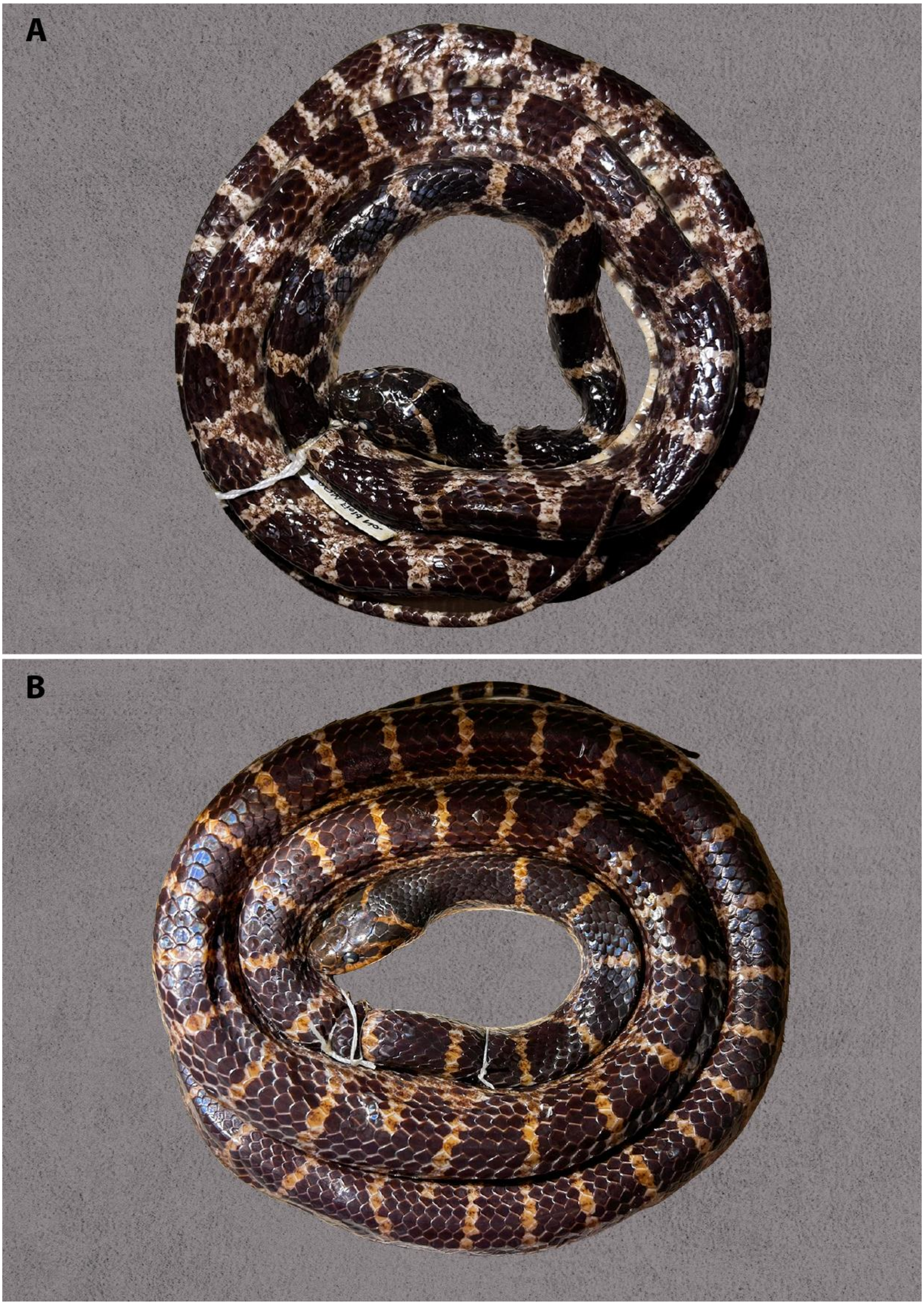

Suppl. Figure S9. *Lycodon rufozonatus* in preservative in general dorsal view. **A** – Specimen ROM 30814 (Adult, male); **B** – Specimen ROM 34615 (adult, female). Photos by A. Lathrop.

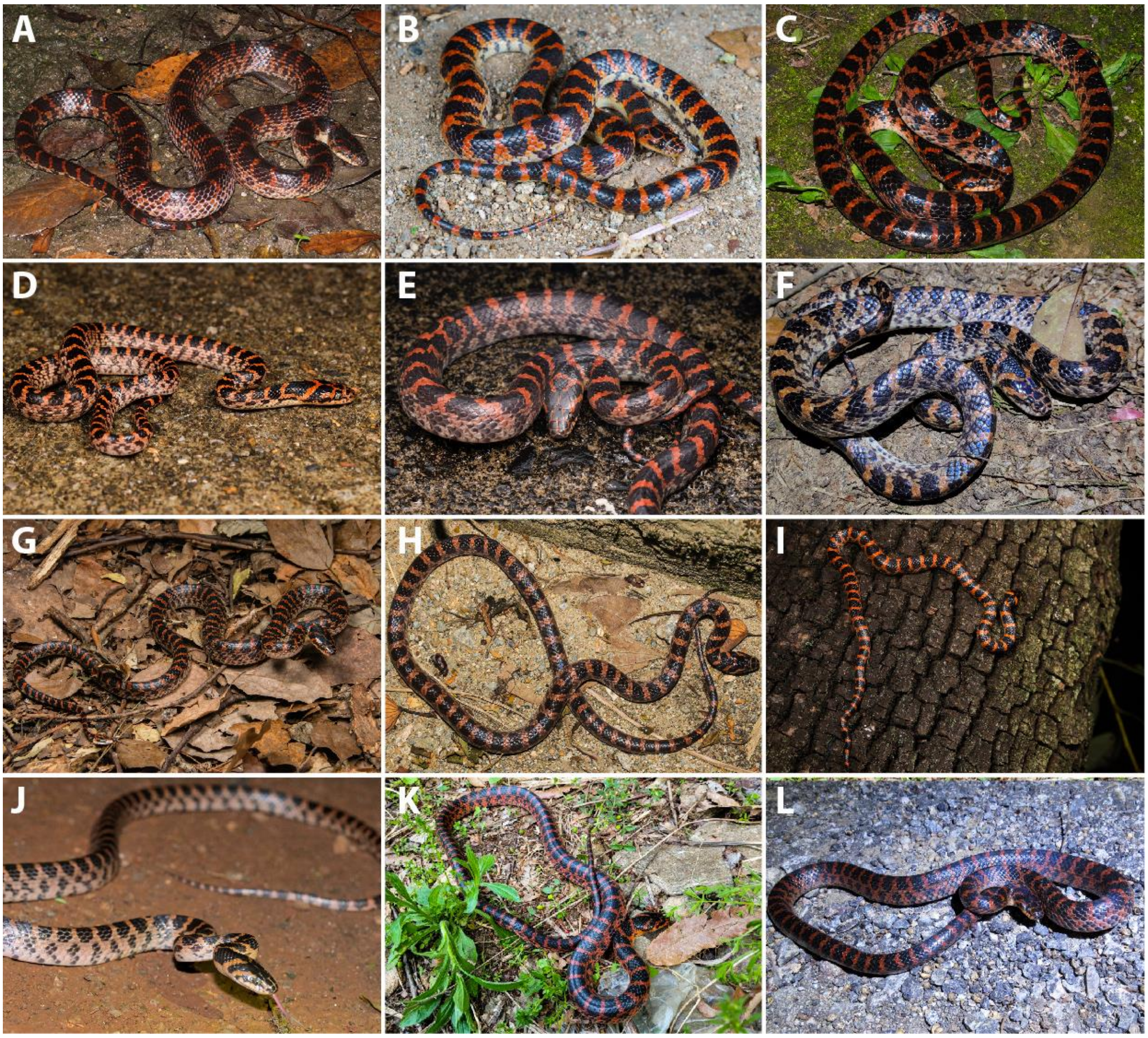

Suppl. Figure S10. *Lycodon rufozonatus* in life (all not collected) – China: from Hangzhou, Zhejiang (**A**); from Beijing, Beijing (**B**); from Chengdu, Sichuan (**C**); from Jian, Jiangxi (**D**); from Lu'an, Anhui (**E**); from Nanjing, Jiangsu (**F**); from Wuhan, Hubei (**G**); from Shenzhen, Guangdong (**H**); from Chuxiong, Yunnan (**I**); from Kunming, Yunnan (**J**); – South Korea: from Anyang, Gyeonggi-do (**K**); from Chuncheon, Gangwon (**L**). Photos by J. Hong (**A**); M.L. Li (**B, G**); G. Vogel (**C**); A. Tianji (**D**); Y.Y. Zhang (**E**); A. Borzée (**F**); G.L. Wu (**H**); Y.X. Wu (**I**); C. Jin (**J**); K.D. Wook (**K**); Y. Shin (**L**).

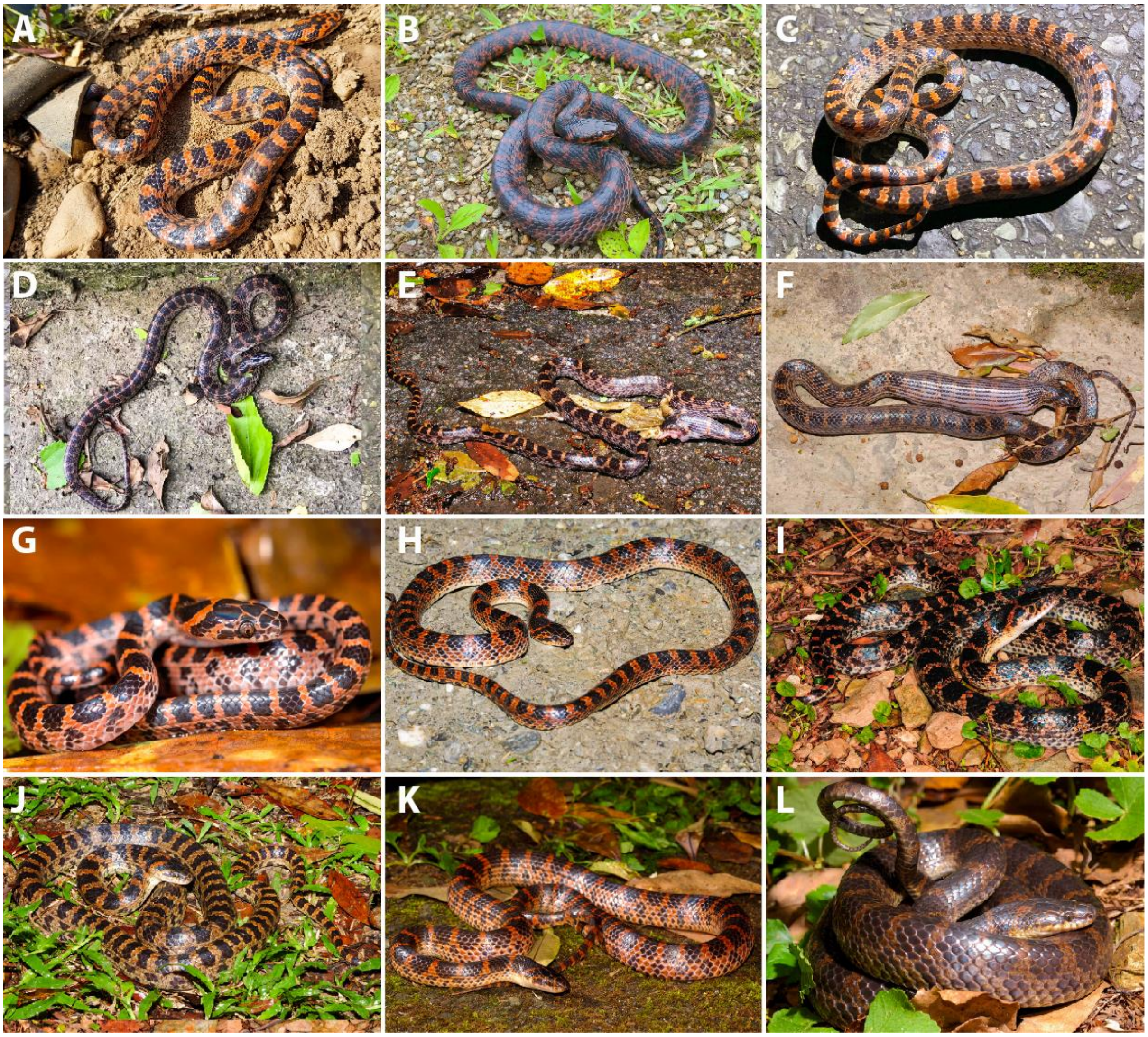

Suppl. Figure S11. *Lycodon rufozonatus* in life (all not collected) – South Korea: from Ongjin, Incheon (**A**); from Sancheong, Gyeongsangnam (**B**) – Japan: from Tsushima, Nagasaki (C); – Vietnam: from Tay Yen Tu NR, Bac Giang (**D**); – Taiwan, China: from Taoyuan (**E-G**); from Hualien (**H**); from Taichung (**I**); from Nantou (**J-L**). Photos by A. Borzée (**A**); Y. Shin (**B**); Chouenyu (**C**); B.T. Nguyen (**D**); C.W. You (**E-L**).

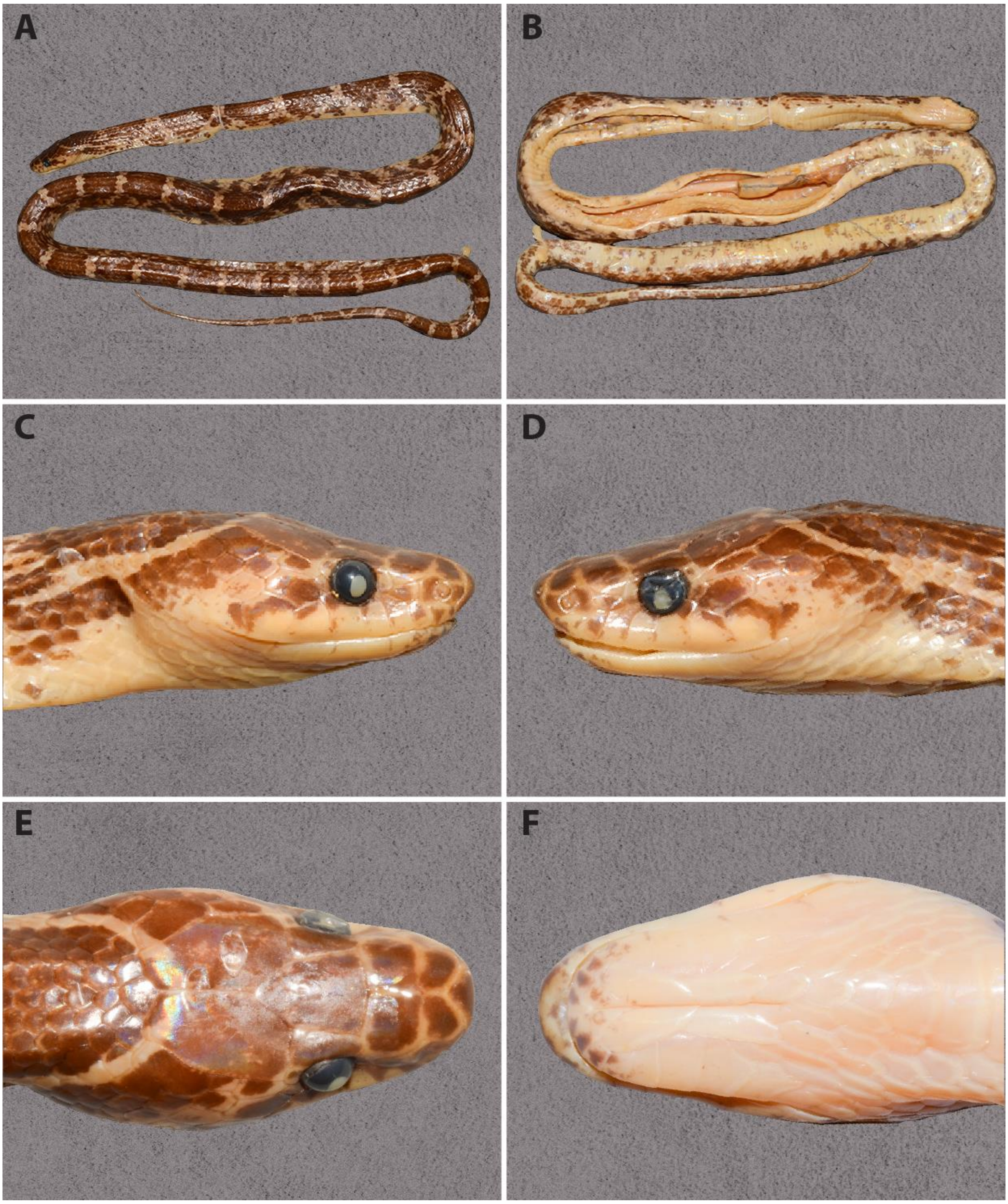

Suppl. Figure S12. *Lycodon walli* **stat. nov.** in preservative – Specimen KUZ R62233 (topotype, adult male): general dorsal view (**A**); general ventral view (**B**); lateral view of the head, right side (**C**); lateral view of the head, left side (**D**); dorsal view of the head (**E**); ventral view of the head (**F**). Photos by G. Vogel.

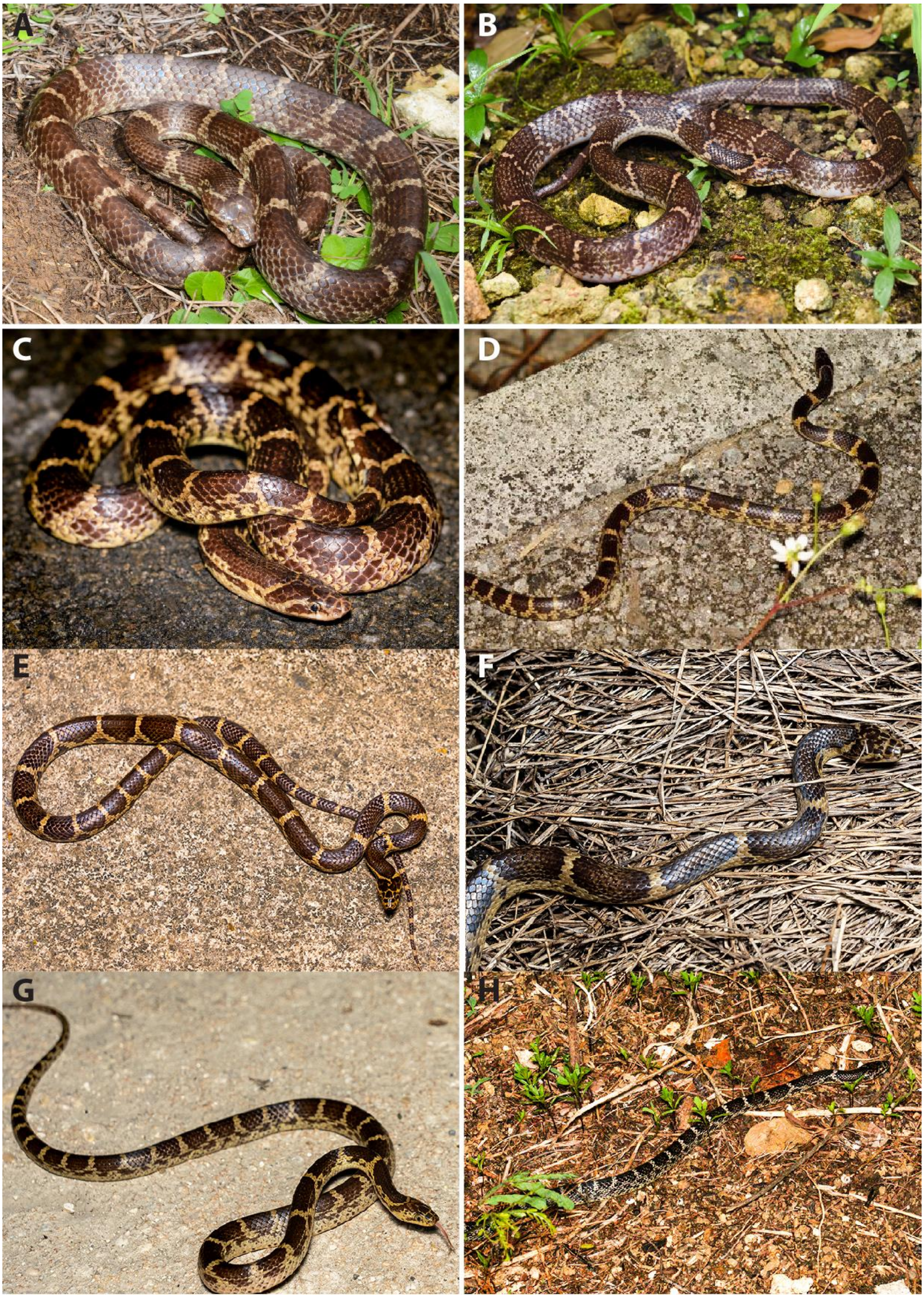

Suppl. Figure S13. *Lycodon walli* **stat. nov.** in life (all not collected): – Ryukyus, Japan: from Miyako Is (**A, B**); from Ishigaki Is (**C**); from Kohama Is (**D**); from Irimote Is (**E**); from Huroshima (**F**); from Taketomi Is (**G**); and Yonaguni Is (**H**). Photos by G. Vogel (**A**); C.W. You (**B**); K. Ito (**C-H**); S. Kanao (**D**).

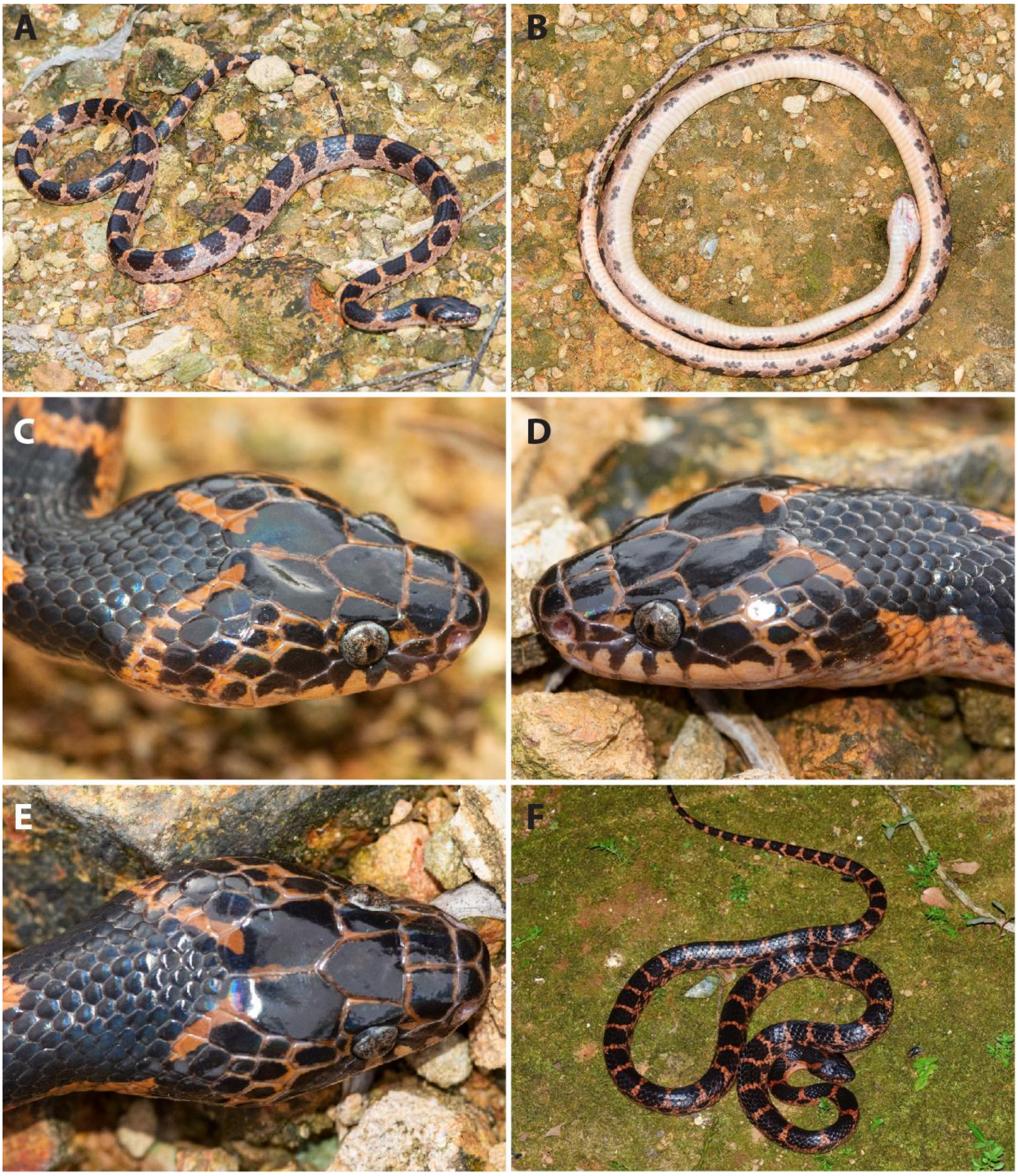

Suppl. Figure S14. *Lycodon duytan* **sp**. **nov**. in life – specimen DTU 541 (paratype, adult female): general dorsal view (**A**); general ventral view (**B**); lateral view of the head, right side (**C**); lateral view of the head, left side (**D**); dorsal view of the head (**E**) – specimen DTU 544 (paratype, adult female): general dorsal view (**F**). Photos by T.V. Nguyen (**A-E**); T. Warfel (**F**).

Suppl. Table S1. Measurements and scale counts of *Lycodon rufozonatus* species complex (encompass: *L. duytan* **sp. nov.**, *L. rufozonatus*, and *L. walli* **stat. nov.**). **Remark**: M = Male; F = Female; SM = Subadult male; SF = Subadult female; J = Juvenile; + = tail incomplete; N/a = not available.

Species	Voucher number	Locality	Sex	Status	SVL (mm)	TaL (mm)	VEN	SC	MSR	KSD
L. duytan	DTU 540	Pu Mat NP, Nghe An, Vietnam	M	Holotype	890	223	229	94	17	5
L. duytan	CPNP NHQ.2017.18	Cuc Phuong NP, Thanh Hoa, Vietnam	F		980	220	225	84	17	5
L. duytan	CPNP NHQ.225	Cuc Phuong NP, Ninh Binh, Vietnam	F		500	125	217	82	17	5
L. duytan	CPNP NHQ.240	Cuc Phuong NP, Ninh Binh, Vietnam	F		810	192	220	89	17	5
L. duytan	DTU 541	Pu Mat NP, Nghe An, Vietnam	F	Paratype	616	146	224	80	17	5
L. duytan	DTU 542	Vu Quang NP, Ha Tinh, Vietnam	F		764	N/a	230	N/a	17	5
L. duytan	DTU 543	Cuc Phuong NP, Ninh Binh, Vietnam	F	Paratype	646	190	230	95	17	5
L. duytan	DTU 544	Cuc Phuong NP, Ninh Binh, Vietnam	F	Paratype	773	183	225	86	17	4
L. duytan	SIFASV 104 (release)	Pu Mat NP, Nghe An, Vietnam	F		861	187+	224	67+	17	4
L. duytan	SIFASV 105 (release)	Pu Mat NP, Nghe An, Vietnam	F		682	158	227	88	17	4
L. duytan	SIFASV 106 (release)	Nam Dong NR, Thanh Hoa, Vietnam	F		713	185	225	95	17	5
L. walli	KUZ 05	Yonaguni, Yaeyama, Ryukyus, Japan	M		547	148	194	86	17	0
L. walli	KUZ 06	Yonaguni, Yaeyama, Ryukyus, Japan	M		703	182	196	89	17	0
L. walli	KUZ 11	Yonaguni, Yaeyama, Ryukyus, Japan	M		575	145	194	79	17	0
L. walli	KUZ 12	Yonaguni, Yaeyama, Ryukyus, Japan	M		520	150	193	86	17	0
L. walli	KUZ 14	Hatoma, Yaeyama, Ryukyus, Japan	M		780	190	189	81	17	0
L. walli	KUZ 15	Hatoma, Yaeyama, Ryukyus, Japan	M		610	150	190	78	17	0
L. walli	KUZ 21	Ishigaki, Yaeyama, Ryukyus, Japan	M		725	215	187	87	17	0
L. walli	KUZ 26	Taketomi, Yaeyama, Ryukyus, Japan	M		568	161	184	86	17	0
L. walli	KUZ 62233	Mt. Omoto, Ishigaki, Yaeyama, Ryukyus, Japan	M		611	183	187	84	17	0
L. walli	KUZ 65597	Iriomote, Yaeyama, Ryukyus, Japan	M		743	205	198	84	17	0
L. walli	KUZ 62999	Iriomote, Yaeyama, Ryukyus, Japan	M		922	243	191	85	17	0
L. walli	TIU 12	Miyako, Rykyu, Japan	M		N/a	N/a	189	73	17	0
L. walli	USNM 34006	Ishigaki, Yaeyama, Ryukyus, Japan	M	Paratype	650	180	195	82	17	0
L. walli	USNM 34007	Ishigaki, Yaeyama, Ryukyus, Japan	M	Holotype	600	190	190	87	17	0
L. walli	Sci. Coll. Kyoto g	Ishigaki, Yaeyama, Ryukyus, Japan	M		620	170	164	71	17	0
L. walli	KUZ 22	Ishigaki, Yaeyama, Ryukyus, Japan	SM		395	115	185	78	17	0
L. walli	KUZ 04	Iriomote, Yaeyama, Ryukyus, Japan	SM		301	81	187	87	17	0
L. walli	KUZ 10	Yaeyama, Ryukyus, Japan	SM		498	152	189	78	17	0
L. walli	KUZ 20	Kuro, Yaeyama, Ryukyus, Japan	SM		260	70	189	87	17	0
L. walli	KUZ 23	Iriomote, Yaeyama, Ryukyus, Japan	SM		260	105	187	80	17	0
L. walli	Sci. Coll. Kyoto c	Ishigaki, Yaeyama, Ryukyus, Japan	SM		455	130	177	77	17	0

L. walli	Sci. Coll. Kyoto h	Ishigaki, Yaeyama, Ryukyus, Japan	SM		332	94	197	85	17	0
L. walli	Sci. Coll. Kyoto f	Ishigaki, Yaeyama, Ryukyus, Japan	SM		360	100	189	75	17	0
L. walli	KUZ 01	Miyako, Yaeyama, Ryukyus, Japan	F		655	175	182	82	17	0
L. walli	KUZ 13	Yaeyama, Ryukyus, Japan	F		580	165	190	81	17	0
L. walli	KUZ 17	Miyako, Yaeyama, Ryukyus, Japan	F		660	170	183	79	17	0
L. walli	KUZ 24	Yonaguni, Yaeyama, Ryukyus, Japan	F		715	176	197	85	17	0
L. walli	KUZ 25	Yonaguni, Yaeyama, Ryukyus, Japan	F		638	178	192	90	17	0
L. walli	KUZ 28	Ikema, Yaeyama, Ryukyus, Japan	F		562	156	183	83	17	0
L. walli	KUZ 30	Irabu, Yaeyama, Ryukyus, Japan	F		521	147	187	81	17	0
L. walli	USNM 34008	Ishigaki, Yaeyama, Ryukyus, Japan	F	Paratype	560	160	186	81	17	0
L. walli	Sci. Coll. Kyoto a	Miyako, Yaeyama, Ryukyus, Japan	F		620	160	187	81	17	0
L. walli	Sci. Coll. Kyoto b	Ishigaki, Yaeyama, Ryukyus, Japan	F		560	130+	186	50+	17	0
L. walli	Sci. Coll. Kyoto e	Ishigaki, Yaeyama, Ryukyus, Japan	F		840	120+	194	N/a	17	0
L. walli	KUZ 02	Miyako, Yaeyama, Ryukyus, Japan	SF		475	125+	180	81+	17	0
L. walli	KUZ 07	Iriomote, Yaeyama, Ryukyus, Japan	SF		310	86	184	84	17	0
L. walli	KUZ 08	Iriomote, Yaeyama, Ryukyus, Japan	SF		334	91	189	83	17	0
L. walli	KUZ 09	Ishigaki, Yaeyama, Ryukyus, Japan	SF		338	90	190	85	17	0
L. walli	Sci. Coll. Kyoto d	Ishigaki, Yaeyama, Ryukyus, Japan	SF		460	80+	185	N/a	17	0
L. walli	KUZ 29	Kohama, Yaeyama, Ryukyus, Japan	SF		486	134	182	77	17	0
L. rufozonatus	USNM 14614	South Korea	M		852	194	195	69	17	0
L. rufozonatus	USNM 29701	Beijing, China	M		640	143	201	67	17	0
L. rufozonatus	FMNH 7529	Changsha, Hunan, China	M	Paratype of *Dinodon rufozonatum williamsi*	672	N/a	207	76+	19	0
L. rufozonatus	FMNH 7530	Changsha, Hunan, China	M	Paratype of *Dinodon rufozonatum williamsi*	1070	238	210	83	19	0
L. rufozonatus	NMBE 1016377	Ningbo, Zhejiang, China	M		615	154	199	72	17	0
L. rufozonatus	USNM 63415	Suifu, Sichuan, China	M		730	193	210	83	17	0
L. rufozonatus	USNM 66458	Hangzhou, Zhejiang, China	M		740	182	189	70	17	0
L. rufozonatus	USNM 67018	Hangzhou, Zhejiang, China	M		765	172	192	67	17	0
L. rufozonatus	USNM 67019	Hangzhou, Zhejiang, China	M		965	N/a	199	69+	17	0
L. rufozonatus	KIZ 821007	Zhaotong, Yunnan, China	M		800	201	216	81	17	0
L. rufozonatus	KIZ 821037	Zhaotong, Yunnan, China	M		829	244	213	82	17	0
L. rufozonatus	KIZ 821038	Kunming, Yunnan, China	M		860	177	216	62	17	0
L. rufozonatus	ZMB 27949	Chengdu, Sichuan, China	M		559	N/a	205	N/a	17	0
L. rufozonatus	NHMW 22771.4	Kaohsiung, Taiwan	M		802	218	203	85	17	0
L. rufozonatus	NHMW 22771.5	Kaohsiung, Taiwan	M		670	201	200	85	17	0
L. rufozonatus	NHMW 22771.7	Kaohsiung, Taiwan	M		765	N/a	199	75+	17	0
L. rufozonatus	NHMW 22771.9	Kaohsiung, Taiwan	M		673	N/a	201	N/a	17	0
L. rufozonatus	NHMW 22772.1	Taiwan	M		807	N/a	195	N/a	17	0
L. rufozonatus	NHMW 22772.2	Taiwan	M		632	183	196	85	17	0
L. rufozonatus	NHMW 22774.2	Anping, Tainan, Taiwan	M		N/a	N/a	194	85	17	0

L. rufozonatus	NHMW 22774.3	Tainan, Taiwan	M		764	186	202	N/a	17	0
L. rufozonatus	NHMW 22774.4	Tainan, Taiwan	M		617	167	198	85	17	0
L. rufozonatus	NHMW 22774.5	Dalin, Taiwan	M		605	181	197	81	17	0
L. rufozonatus	NHMW 22775.1	Taiwan	M		780	202	200	83	17	0
L. rufozonatus	ZMB 27711	Dali, Yunnan, China	M	Syntype of *Dinodon rufozonatum yunnanense*	674	142	187	60	17	0
L. rufozonatus	ZMB 65448	Yunnan, China	M	Syntype of *Dinodon rufozonatum yunnanense*	589	161	190	77	17	0
L. rufozonatus	ZMB 65446	Yunnan, China	M	Syntype of *Dinodon rufozonatum yunnanense*	685	162	191	74	17	0
L. rufozonatus	ZMB 18473	Qingdao, Shandong, China	M		1001	204	196	72	17	0
L. rufozonatus	ZMB 19329	Shandong, China	M		1122	201	203	71	17	0
L. rufozonatus	ZMB 24830B	Qingdao, Shandong, China	M		504	109	204	77	17	0
L. rufozonatus	Sci. Coll. Kyoto f	Gyeongseong, Soujt Korea	M		851	213	209	74	17	0
L. rufozonatus	Sci. Coll. Kyoto k	Tsushima, Nagasaki, Japan	M		796	188	203	80	17	0
L. rufozonatus	MHMUK 1843.7.21.36	Zhoushan, Zhejiang, China	SM	Holotype	370	92	198	74	17	0
L. rufozonatus	NHMW 22771.1	Kaohsiung, Taiwan	SM		277	74	206	84	17	0
L. rufozonatus	NHMW 22771.8	Kaohsiung, Taiwan	SM		467	137	197	88	17	0
L. rufozonatus	NHMW 22773.2	Keelung, Taiwan	SM		418	128	197	87	17	0
L. rufozonatus	ZMB 52629	Dali, Yunnan, China	SM	Syntype of *Dinodon rufozonatum yunnanense*	301	67	190	75	17	0
L. rufozonatus	Sci. Coll. Kyoto l	Tsushima, Nagasaki, Japan	SM		200	47	209	79	17	0
L. rufozonatus	Sci. Coll. Kyoto m	Tsushima, Nagasaki, Japan	SM		241	51	207	68	17	0
L. rufozonatus	ZMB 18474	Qingdao, Shandong, China	F		621	138	201	68	17	0
L. rufozonatus	KIZ 821021	Zhaotong, Yunnan, China	F		693	151	190	60	17	0
L. rufozonatus	NHBE 1016379	Fujian, China	F		707	170	214	81	17	0
L. rufozonatus	RMNH 4591	Jiangxi, China	F		1015	N/a	206	41+	19	0
L. rufozonatus	USNM 67016	Hangzhou, Zhejiang, China	F		860	181	189	65	17	0
L. rufozonatus	NHMW 22765.1	Pingshan, Nantou, Taiwan	F		955	N/a	215	N/a	19	0
L. rufozonatus	NHMW 22765.2	Pingshan, Nantou, Taiwan	F		645	N/a	209	N/a	19	0
L. rufozonatus	SMF 18045	Taiwan	F	Holotype of *Dinodon rufozonatus formosana*	512	132	201	76	17	0
L. rufozonatus	USNM 146714	Taiwan	F		690	167	210	77	17	0
L. rufozonatus	USNM 146714	Taiwan	F		690	167	210	77	17	0
L. rufozonatus	USNM 291654	Hualien, Taiwan	F		750	176	195	73	17	0
L. rufozonatus	USNM 291654	Hualien, Taiwan	F		750	176	195	73	17	0

L. rufozonatus	ZMB 65447	Yunnan, China	F	Syntype of *Dinodon rufozonatum yunnanense*	559	143	190	61	17	0
L. rufozonatus	ZMB 9786 A	Ningbo, Zhejiang, China	F		902	171	196	68	17	0
L. rufozonatus	ZMB 19328	Shandong, China	F		889	66+	202	31+	17	0
L. rufozonatus	ZMB 24830A	Qingdao, Shandong, China	F		1145	204	196	65	17	0
L. rufozonatus	ZMB 26606	Qingdao, Shandong, China	F		504	109	201	67	17	0
L. rufozonatus	Sci. Coll. Kyoto g	Gyeongseong, Soujt Korea	F		947	201	201	68	17	0
L. rufozonatus	Sci. Coll. Kyoto i	Gyeongseong, Soujt Korea	F		857	163	205	72	17	0
L. rufozonatus	Sci. Coll. Keijo	Gyeongseong, Soujt Korea	F		630	170	210	63	17	0
L. rufozonatus	SMF 55833	Busan, Korea	SF		402	88	200	68	17	0
L. rufozonatus	NHMW 22771.2	Kaohsiung, Taiwan	SF		416	118	204	85	17	0
L. rufozonatus	NHMW 22771.3	Kaohsiung, Taiwan	SF		475	N/a	199	79+	17	0
L. rufozonatus	NHMW 22772.3	Kankau, Pingtung, Taiwan	SF		456	124	190	80	17	0
L. rufozonatus	NHMW 22772.4	Kankau, Pingtung, Taiwan	SF		289	90	188	86	17	0
L. rufozonatus	NHMW 22774,1	Chiayi, Taiwan	SF		494	130	196	78	17	0
L. rufozonatus	ZMB 52630	Dali, Yunnan, China	SF	Syntype of *Dinodon rufozonatum yunnanense*	350	74	189	64	17	0
L. rufozonatus	ZMB 52631	Dali, Yunnan, China	SF	Syntype of *Dinodon rufozonatum yunnanense*	206	43	187	65	17	0
L. rufozonatus	ZMB 65449	Yunnan, China	SF	Syntype of *Dinodon rufozonatum yunnanense*	488	131	190	66	17	0
L. rufozonatus	ZMB 65450	Yunnan, China	SF	Syntype of *Dinodon rufozonatum yunnanense*	453	118	187	73	17	0
L. rufozonatus	ZMB 65451	Yunnan, China	SF	Syntype of *Dinodon rufozonatum yunnanense*	193	49	191	69	17	0
L. rufozonatus	ZMB 9786 B	Ningbo, Zhejiang, China	SF		216	49	196	69	17	0
L. rufozonatus	ZMB 24200	Taiwan	SF		424	126	186	81	17	0
L. rufozonatus	Sci. Coll. Kyoto h	Gyeongseong, Soujt Korea	SF		258	61	206	82	17	0
L. rufozonatus	Sci. Coll. Kyoto j	Gyeongseong, Soujt Korea	SF		269	57	207	68	17	0
L. rufozonatus	N/a	N/a	N/a	Holotype of *Dinodon cancellatum*	627	182	194	68	17	0
L. rufozonatus	ANSP 3477	Ningbo, Zhejiang, China	F	Holotype of *Coronella striata*	1015	226	193	70	17	0

Suppl. Table S1. (continued)

Species	Voucher number	Sex	SL	IL	PrO	PoO	Lor	Lor-E	AT	PT	BB	TB	Source
L. duytan	DTU 540	M	8/8	9/9	1/1	2/2	1/1	no/no	2/2	2/2	49	23	This study
L. duytan	CPNP NHQ.2017.18	F	8/8	9/9	1/1	2/2	1/1	yes/yes	2/2	2/2	44	24	This study
L. duytan	CPNP NHQ.225	F	8/8	9/9	1/1	2/2	1/1	yes/yes	2/2	2/2	48	24	This study
L. duytan	CPNP NHQ.240	F	8/8	9/9	1/1	2/2	1/1	yes/yes	2/2	2/2	46	21	This study
L. duytan	DTU 541	F	8/8	9/9	1/1	2/2	1/1	no/no	2/2	2/2	40	14	This study
L. duytan	DTU 542	F	8/8	9/9	1/1	2/2	1/1	yes/yes	2/2	2/2	49	N/a	This study
L. duytan	DTU 543	F	8/8	9/9	1/1	2/2	1/1	yes/yes	2/2	2/2	47	20	This study
L. duytan	DTU 544	F	8/8	9/9	1/1	2/2	1/1	no/no	2/2	2/2	44	15	This study
L. duytan	SIFASV 104 (release)	F	8/8	10/10	1/1	2/2	1/1	yes/yes	2/2	2/2	47	16+	This study
L. duytan	SIFASV 105 (release)	F	8/8	10/10	1/1	2/2	1/1	yes/yes	2/2	2/2	48	15	This study
L. duytan	SIFASV 106 (release)	F	8/8	10/10	1/1	2/2	1/1	yes/yes	2/2	2/2	40	16	This study
L. walli	KUZ 05	M	8/8	N/a	N/a	N/a	1/1	N/a	N/a	N/a	N/a	N/a	Takara (1962)
L. walli	KUZ 06	M	8/8	N/a	N/a	N/a	1/1	N/a	N/a	N/a	N/a	N/a	Takara (1962)
L. walli	KUZ 11	M	8/8	N/a	N/a	N/a	1/1	N/a	N/a	N/a	N/a	N/a	Takara (1962)
L. walli	KUZ 12	M	8/8	N/a	N/a	N/a	1/1	N/a	N/a	N/a	N/a	N/a	Takara (1962)
L. walli	KUZ 14	M	8/8	N/a	N/a	N/a	1/1	N/a	N/a	N/a	N/a	N/a	Takara (1962)
L. walli	KUZ 15	M	7/7	N/a	N/a	N/a	1/1	N/a	N/a	N/a	N/a	N/a	Takara (1962)
L. walli	KUZ 21	M	8/8	N/a	N/a	N/a	1/1	N/a	N/a	N/a	N/a	N/a	Takara (1962)
L. walli	KUZ 26	M	8/8	N/a	N/a	N/a	1/1	N/a	N/a	N/a	N/a	N/a	Takara (1962)
L. walli	KUZ 62233	M	8/8	10/10	1/1	2/2	1/1	no/no	2/2	3/3	30	16	This study
L. walli	KUZ 65597	M	8/8	10/10	1/1	2/2	1/1	no/no	2/2	3/3	34	16	This study
L. walli	KUZ 62999	M	8/8	10/10	1/1	2/2	1/1	no/no	2/2	3/3	27	16	This study
L. walli	TIU 12	M	8/8	10/10	N/a	N/a	1/1	N/a	N/a	N/a	29	15	Stejneger (1907)
L. walli	USNM 34006	M	8/8	10/10	1/1	2/2	1/1	no/no	2/2	3/3	29	16	Stejneger (1907); this study
L. walli	USNM 34007	M	8/8	10/10	1/1	2/2	1/1	no/no	2/2	3/3	25	18	Stejneger (1907); this study
L. walli	Sci. Coll. Kyoto g	M	8/8	N/a	N/a	N/a	1/1	N/a	2/2	3/3	30	16	Maki (1931)
L. walli	KUZ 22	SM	8/8	N/a	N/a	N/a	1/1	N/a	N/a	N/a	N/a	N/a	Takara (1962)
L. walli	KUZ 04	SM	8/8	N/a	N/a	N/a	1/1	N/a	N/a	N/a	N/a	N/a	Takara (1962)
L. walli	KUZ 10	SM	8/8	N/a	N/a	N/a	1/1	N/a	N/a	N/a	N/a	N/a	Takara (1962)
L. walli	KUZ 20	SM	8/8	N/a	N/a	N/a	1/1	N/a	N/a	N/a	N/a	N/a	Takara (1962)
L. walli	KUZ 23	SM	8/8	N/a	N/a	N/a	1/1	N/a	N/a	N/a	N/a	N/a	Takara (1962)
L. walli	Sci. Coll. Kyoto c	SM	8/8	N/a	N/a	N/a	1/1	N/a	2/2	3/3	27	17	Maki (1931)
L. walli	Sci. Coll. Kyoto h	SM	8/8	N/a	N/a	N/a	1/1	N/a	2/2	3/3	33	18	Maki (1931)
L. walli	Sci. Coll. Kyoto f	SM	8/8	N/a	N/a	N/a	1/1	N/a	2/2	3/3	24	15	Maki (1931)
L. walli	KUZ 01	F	8/8	N/a	N/a	N/a	1/1	N/a	N/a	N/a	N/a	N/a	Takara (1962)
L. walli	KUZ 13	F	8/8	N/a	N/a	N/a	1/1	N/a	N/a	N/a	N/a	N/a	Takara (1962)

L. walli	KUZ 17	F	9/9	N/a	N/a	N/a	1/1	N/a	N/a	N/a	N/a	N/a	Takara (1962)
L. walli	KUZ 24	F	8/8	N/a	N/a	N/a	1/1	N/a	N/a	N/a	32	19	Takara (1962)
L. walli	KUZ 25	F	8/8	N/a	N/a	N/a	1/1	N/a	N/a	N/a	N/a	N/a	Takara (1962)
L. walli	KUZ 28	F	8/8	N/a	N/a	N/a	1/1	N/a	N/a	N/a	N/a	N/a	Takara (1962)
L. walli	KUZ 30	F	8/8	N/a	N/a	N/a	1/1	N/a	N/a	N/a	N/a	N/a	Takara (1962)
L. walli	USNM 34008	F	8/8	N/a	N/a	N/a	1/1	N/a	N/a	N/a	26	18	Stejneger (1907)
L. walli	Sci. Coll. Kyoto a	F	8/8	N/a	N/a	N/a	1/1	N/a	2/2	3/3	28	19	Maki (1931)
L. walli	Sci. Coll. Kyoto b	F	8/8	N/a	N/a	N/a	1/1	N/a	2/2	3/3	27	9+	Maki (1931)
L. walli	Sci. Coll. Kyoto e	F	8/8	N/a	N/a	N/a	1/1	N/a	2/2	3/3	34	12+	Maki (1931)
L. walli	KUZ 02	SF	8/8	N/a	N/a	N/a	1/1	N/a	N/a	N/a	N/a	N/a	Takara (1962)
L. walli	KUZ 07	SF	8/8	N/a	N/a	N/a	1/1	N/a	N/a	N/a	N/a	N/a	Takara (1962)
L. walli	KUZ 08	SF	8/8	N/a	N/a	N/a	1/1	N/a	N/a	N/a	N/a	N/a	Takara (1962)
L. walli	KUZ 09	SF	8/8	N/a	N/a	N/a	1/1	N/a	N/a	N/a	N/a	N/a	Takara (1962)
L. walli	Sci. Coll. Kyoto d	SF	8/8	N/a	N/a	N/a	1/1	N/a	2/2	3/3	32	10	Maki (1931)
L. walli	KUZ 29	SF	8/8	N/a	N/a	N/a	1/1	N/a	N/a	N/a	N/a	N/a	Takara (1962)
L. rufozonatus	USNM 14614	M	8/8	10/10	1/1	2/2	1/1	yes/yes	2/2	3/3	60	22	This study
L. rufozonatus	USNM 29701	M	8/8	10/10	1/1	2/2	1/1	no/no	2/2	3/3	52	15	This study
L. rufozonatus	FMNH 7529	M	8/8	10/10	1/1	2/2	1/1	yes/yes	2/2	3/3	68	24+	This study
L. rufozonatus	FMNH 7530	M	8/8	11/10	1/2	2/2	1/1	yes/yes	2/2	3/3	78	28	This study
L. rufozonatus	NMBE 1016377	M	8/8	10/10	1/1	2/2	1/1	no/no	2/2	3/3	45	20	This study
L. rufozonatus	USNM 63415	M	8/8	10/11	1/1	2/2	1/1	no/yes	2/2	3/3	77	24	This study
L. rufozonatus	USNM 66458	M	8/8	10/10	1/1	2/2	1/1	no/no	2/2	3/3	53	19	This study
L. rufozonatus	USNM 67018	M	8/8	9/9	1/1	2/2	1/1	yes/yes	2/2	3/3	64	21	This study
L. rufozonatus	USNM 67019	M	8/8	11/10	1/1	2/2	1/1	yes/yes	2/2	3/3	70	25	This study
L. rufozonatus	KIZ 821007	M	8/8	10/10	1/1	2/2	1/1	N/a	2/2	3/3	66	23	Yang & Rao (2008)
L. rufozonatus	KIZ 821037	M	8/8	10/10	1/1	2/2	1/1	N/a	2/2	3/3	72	21	Yang & Rao (2008)
L. rufozonatus	KIZ 821038	M	8/8	10/10	1/1	2/2	1/1	N/a	2/2	3/3	70	19	Yang & Rao (2008)
L. rufozonatus	ZMB 27949	M	8/8	10/10	1/1	2/2	1/1	yes/yes	2/2	2/2	65	18+	This study
L. rufozonatus	NHMW 22771.4	M	8/8	10/10	1/1	2/2	1/1	no/no	2/2	3/3	50	24	This study
L. rufozonatus	NHMW 22771.5	M	8/8	10/10	1/1	2/2	1/1	no/no	2/2	3/3	54	26	This study
L. rufozonatus	NHMW 22771.7	M	8/8	10/10	1/1	2/2	1/1	no/no	2/2	3/3	50	24	This study
L. rufozonatus	NHMW 22771.9	M	8/8	10/10	2/2	2/2	1/1	no/no	2/2	3/3	43	13+	This study
L. rufozonatus	NHMW 22772.1	M	9/8	10/10	1/1	2/2	1/1	no/yes	2/2	3/3	42	12+	This study
L. rufozonatus	NHMW 22772.2	M	8/8	10/10	1/1	2/2	1/1	no/no	2/2	3/3	54	24	This study
L. rufozonatus	NHMW 22774.2	M	8/8	10/10	1/1	2/2	1/1	no/no	2/2	3/3	54	21	This study
L. rufozonatus	NHMW 22774.3	M	8/8	10/10	2/2	2/2	1/1	no/no	2/2	2/2	56	26	This study
L. rufozonatus	NHMW 22774.4	M	8/8	10/10	1/1	2/3	1/1	no/no	2/2	3/3	62	23	This study
L. rufozonatus	NHMW 22774.5	M	8/8	10/10	1/1	2/2	1/1	no/no	2/2	3/3	53	24	This study
L. rufozonatus	NHMW 22775.1	M	8/8	10/10	1/1	2/2	1/1	no/no	2/2	3/3	?	?	This study

L. rufozonatus	ZMB 27711	M	8/8	10/10	1/1	2/2	1/1	yes/yes	2/2	3/3	62		This study
L. rufozonatus	ZMB 65448	M	8/8	10/10	1/1	2/2	1/1	yes/yes	2/2	3/3	60		This study
L. rufozonatus	ZMB 65446	M	8/8	10/10	1/1	2/2	1/1	yes/yes	2/2	3/3	63		This study
L. rufozonatus	ZMB 18473	M	8/8	10/10	1/1	2/2	1/1	yes/yes	2/2	3/3	79		This study
L. rufozonatus	ZMB 19329	M	7/7	10/10	1/1	2/2	1/1	yes/yes	2/2	3/3	80		This study
L. rufozonatus	ZMB 24830B	M	8/8	10/9	1/1	2/2	1/1	yes/yes	2/2	3/3	84		This study
L. rufozonatus	Sci. Coll. Kyoto f	M	8/8	N/a	1/1	2/2	1/1	N/a	2/2	3/3	61	26	Maki (1931)
L. rufozonatus	Sci. Coll. Kyoto k	M	8/8	N/a	1/1	2/2	1/1	N/a	2/2	3/3	72	25	Maki (1931)
L. rufozonatus	MHMUK 1843.7.21.36	SM	8/8	10/10	1/1	1/2	1/1	yes/yes	2/2	3/3	60	20	This study
L. rufozonatus	NHMW 22771.1	SM	8/8	10/10	1/1	2/2	1/1	no/no	2/2	3/3	54	20	This study
L. rufozonatus	NHMW 22771.8	SM	9/8	10/10	1/1	2/2	1/1	no/no	2/2	3/3	54	30	This study
L. rufozonatus	NHMW 22773.2	SM	8/8	10/10	1/1	2/2	1/1	no/no	2/2	3/3	65	29	This study
L. rufozonatus	ZMB 52629	SM	8/8	10/10	1/1	2/2	1/1	yes/yes	2/2	3/3	63		This study
L. rufozonatus	Sci. Coll. Kyoto l	SM	8/8	N/a	1/1	2/2	1/1	N/a	2/2	3/3	73	26	Maki (1931)
L. rufozonatus	Sci. Coll. Kyoto m	SM	8/8	N/a	1/1	2/2	1/1	N/a	2/2	3/3	68	22	Maki (1931)
L. rufozonatus	ZMB 18474	F	8/8	10/10	1/1	2/2	1/1	yes/yes	2/2	3/3	61	18	This study
L. rufozonatus	KIZ 821021	F	8/8	10/10	1/1	2/2	1/1	N/a	2/2	3/3	50	13	Yang & Rao (2008)
L. rufozonatus	NHBE 1016379	F	8/8	10/10	1/1	1/2	1/1	no/no	2/2	3/3	54	17	This study
L. rufozonatus	RMNH 4591	F	8/8	11/11	0	2/2	1/1	yes/yes	2/2	3/3	65	10+	This study
L. rufozonatus	USNM 67016	F	8/8	10/10	1/1	2/2	1/1	yes/yes	2/2	3/3	65	19	This study
L. rufozonatus	NHMW 22765.1	F	8/8	10/10	1/1	2/2	1/1	yes/yes	2/2	3/3	66	20+	This study
L. rufozonatus	NHMW 22765.2	F	8/8	10/10	1/1	2/2	1/1	yes/yes	2/2	3/3	68	17+	This study
L. rufozonatus	SMF 18045	F	7/8	9/9	1/1	2/2	1/1	no/no	2/2	2/2	54	22	This study
L. rufozonatus	USNM 146714	F	8/8	10/10	1/1	2/2	1/1	no/no	2/1	3/3	64	27	This study
L. rufozonatus	USNM 146714	F	8/8	10/10	1/1	2/2	1/1	no/no	2/1	3/3	64	27	This study
L. rufozonatus	USNM 291654	F	8/8	10/10	1/1	2/2	1/1	no/no	2/1	3/3	50	21	This study
L. rufozonatus	USNM 291654	F	8/8	10/10	1/1	2/2	1/1	no/no	2/1	3/3	50	21	This study
L. rufozonatus	ZMB 65447	F	8/8	10/10	1/1	1/2	1/1	yes/yes	2/2	3/3	64		This study
L. rufozonatus	ZMB 9786 A	F	8/7	9/10	1/1	2/2	1/1	yes/yes	2/2	3/3	73		This study
L. rufozonatus	ZMB 19328	F	8/8	10/10	2/2	2/2	1/1	yes/yes	2/2	3/3	75		This study
L. rufozonatus	ZMB 24830A	F	8/8	10/9	1/1	2/2	1/1	yes/yes	2/2	3/3	79		This study
L. rufozonatus	ZMB 26606	F	8/8	10/10	1/1	2/2	1/1	yes/yes	2/2	3/3	75		This study
L. rufozonatus	Sci. Coll. Kyoto g	F	8/8	N/a	1/1	2/2	1/1	N/a	2/2	3/3	65	23	Maki (1931)
L. rufozonatus	Sci. Coll. Kyoto i	F	8/8	N/a	1/1	2/2	1/1	N/a	2/2	3/3	73	20	Maki (1931)
L. rufozonatus	Sci. Coll. Keijo	F	8/8	N/a	1/1	2/2	1/1	N/a	2/2	3/3	75	17	Maki (1931)
L. rufozonatus	SMF 55833	SF	7/8	10/10	1/1	2/2	1/1	yes/yes	2/2	3/3	66	22	This study
L. rufozonatus	NHMW 22771.2	SF	8/8	10/10	1/1	2/2	1/1	no/no	2/2	3/3	50	25	This study
L. rufozonatus	NHMW 22771.3	SF	8/8	10/10	2/2	2/2	1/1	no/no	2/2	3/3	52	21+	This study
L. rufozonatus	NHMW 22772.3	SF	8/8	10/10	1/1	2/2	1/1	no/no	2/2	2/2	57	22	This study

L. rufozonatus	NHMW 22772.4	SF	8/8	11/11	1/1	2/2	1/1	no/no	2/2	3/3	46	22	This study
L. rufozonatus	NHMW 22774,1	SF	8/8	10/11	1/1	2/2	1/1	no/no	2/2	2/3	61	27	This study
L. rufozonatus	ZMB 52630	SF	8/8	9/8	1/1	1/2	1/1	yes/yes	2/2	2/3	64		This study
L. rufozonatus	ZMB 52631	SF	8/8	10/9	1/1	1/1	1/1	yes/yes	2/2	3/3	64		This study
L. rufozonatus	ZMB 65449	SF	8/8	9/9	1/1	2/2	1/1	yes/yes	2/2	3/3	61		This study
L. rufozonatus	ZMB 65450	SF	8/8	10/10	1/1	1/2	1/1	yes/yes	2/2	3/3	68		This study
L. rufozonatus	ZMB 65451	SF	8/8	8/8	1/1	2/2	1/1	yes/yes	2/2	3/3	69		This study
L. rufozonatus	ZMB 9786 B	SF	8/8	9/9	1/1	2/2	1/1	yes/yes	2/2	2/3	77		This study
L. rufozonatus	ZMB 24200	SF	8/8	10/9	1/1	2/2	1/1	yes/yes	2/2	3/3	63		This study
L. rufozonatus	Sci. Coll. Kyoto h	SF	8/8	N/a	1/1	2/2	1/1	N/a	2/2	3/3	61	26	Maki (1931)
L. rufozonatus	Sci. Coll. Kyoto j	SF	8/8	N/a	1/1	2/2	1/1	N/a	2/2	3/3	74	22	Maki (1931)
L. rufozonatus	Unknown	Unknown	8/8	N/a	N/a	N/a	N/a	N/a	N/a	N/a	70		Duméril et al. (1854)
L. rufozonatus	ANSP 3477	F	8/8	10/10	1/1	2/2	1/1	yes/yes	2/2	3/3	47	13	Hallowell (1856)

Suppl. Table S2. Summary statistics of the principal components analysis (PCA), showing the highest loadings of each morphological character examined and the proportion of variance of each principal component.

Factor	PC1	PC2
**Eigenvalue**	1.7704	1.2506
**Cumulative Eigenvalue**	1.7704	3.0210
**% Total Variance**	44.2595	31.2647
**Cumulative % Total Variance**	44.2595	75.5241
**VEN** (**Loading factor**)	0.4804	0.1998
**SC** (**Loading factor**)	0.2983	0.7937
**BB** (**Loading factor**)	0.5527	-0.5740
**TB** (**Loading factor**)	0.6122	-0.0253

Suppl. Table S3. DNA sequences, voucher specimens, GenBank accession numbers of *Lycodon* and outgroup taxa used in this study

Species	Specimen ID	Location	Cyt b	Reference
Lycodon davisonii	FMNH 255034	Boualapha, Khammouan, Laos	KX660473	Figueroa et al. (2016)
Lycodon nympha	RAP 0536	Kandalam, Matale, Sri Lanka	KC347476	Pyron et al. (2013)
Lycodon subannulatus	LSUHC 5576	Sibu, Johor, Malaysia	KX660499	Figueroa et al. (2016)
Lycodon tristrigatus	FMNH 269033	Bintulu, Sarawak, Malaysia	KX660474	Figueroa et al. (2016)
Lycodon alcalai	KU 327847	Bataan, Batanes, Philippines	KC010344	Siler et al. (2013)
Lycodon anakradaya	SIEZC 20247	Song Giang, Khanh Hoa, Vietnam	OM674283	Nguyen et al. (2022)
Lycodon anakradaya	SIEZC 20248	Song Giang, Khanh Hoa, Vietnam	OM674284	Nguyen et al. (2022)
Lycodon bibonius	KU 304589	Cagayan, Philippines	KC010351	Siler et al. (2013)
Lycodon chrysoprateros	KU 307720	Dalupiri, Cagayan, Philippines	KC010360	Siler et al. (2013)
Lycodon dumerilli	KU 305168	Dinagat, Philippines	KC010362	Siler et al. (2013)
Lycodon muelleri	KU 323384	Luzon, Aurora, Philippines	KC010374	Siler et al. (2013)
Lycodon albofuscus	USMHC 1457	Penang, Malaysia	KX822584	Wostl et al. (2017)
Lycodon ruhstrati abditus	GP 2049	Guangdong, China	KC733200	Guo et al. (2013)
Lycodon cf. *aulicus*	-	Snake Transit, Jabalpur, Madhya Pradesh, India	HQ735416	Dubey et al. (2012)
Lycodon banksi	VNUF R.2015.20	Phou Hin Poun, Khammouane, Laos	MH669272	Luu et al. (2018)
Lycodon butleri	LSUHC 9136	Bukit Larut, Perak, Malaysia	KC010353	Grismer et al. (2014)
Lycodon capucinus	LSUHC 9277	Nam Du, Kien Giang, Vietnam	KC010356	Siler et al. (2013)
Lycodon carinatus	RAP 0447	Kanneliya, Galler, Sri Lanka	KC347486	Pyron et al. (2013)
Lycodon cathaya	SYS r001542=CHS770	Huaping, Longsheng, Guangxi, China	MT602075	Li et al. (2020)
Lycodon cavernicolus	LSUHC 9985	Gua Wang Burma, Perlis, Malaysia	KJ607889	Grismer et al. (2014)
Lycodon chapaensis	KIZ 034331	Xichou, Wenshan, Yunnan, China	MW353739	Wang et al. (2021)
Lycodon deccanensis	BNHS 3610	Devarayanadurga, Tumkur, Karnataka, India	MW006486	Kalki et al. (2020)
Lycodon effraenis	LSUHC 9670	Kedah, Malaysia	KC010376	Siler et al. (2013)
Lycodon fasciatus	CAS 234875	Midat, Chin, Myanmar	KC010365	Siler et al. (2013)
Lycodon latifasciatus	KIZ YPX46121	Medog, Tibet, China	MW199788	Che et al. (2020)
Lycodon flavicolis	-	Devarayanadurga, Karnataka, India	MW006488	Kalki et al. (2020)
Lycodon flavozonatus	GP 2279	Guangdong, China	KC733210	Guo et al. (2013)
Lycodon futsingensis	GP 2226	Guangdong, China	KC733207	Guo et al. (2013)
Lycodon gongshan	KIZ 035112	Dulongjiang, Nujiang, Yunnan, China	MW353748	Wang et al. (2021)
Lycodon jara	CAS 235387	Kachin, Myanmar	KC010367	Siler et al. (2013)
Lycodon laoensis	FMNH 258659	Salavan, Laos	KC010368	Siler et al. (2013)
Lycodon liuchengchaoi	GP 2094	Nanling, Guangdong, China	KC733201	Guo et al. (2013)
Lycodon bicolor	ADR 197	Mussoorie, Uttarakhand, India	MW862977	Nawani et al. (2021)
Lycodon neomaculatus	SYS r001943	Shimentai NR, Yingde, Guangdong, China	MT625859	Wang et al. (2020)
Lycodon meridionalis	VNUF R.2017.54	Cuc Phuong NP, Ninh Binh, Vietnam	MH669268	Luu et al. (2018)
Lycodon multizonatus	CIB 103	Luding, Sichuan, China	KF732926	Lei et al. (2014)

Lycodon obvelatus	KIZ 040146	Panzhihua, Sichuan, China	MW353745	Wang et al. (2021)
Lycodon pictus	IEBR 4166	Trung Khanh, Cao Bang, Vietnam	MT845093	Janssen et al. (2019)
Lycodon rosozonatus	SYS r001617=CHS794	Jianfengling, Hainan, China	MK201531	Li et al. (2020)
Lycodon rufozonatus	CIB101=DL12611	China	KF732924	Lei et al. (2014)
Lycodon rufozonatus	SYS r001770	Mt. Tiantai, Zhejiang, China	MT625858	Wang et al. (2020)
Lycodon rufozonatus	SYS r002061	Yangjifeng NNR, Guixi, Jiangxi, China	MT625860	Wang et al. (2020)
Lycodon rufozonatus	GP 133	Sichuan, China	KC733194	Guo et al. (2013)
Lycodon rufozonatus	CIB 098274	China	JF827672	Guo et al. (2013)
Lycodon rufozonatus	SYS r000909=CHS601	Huangshan, Anhui, China	MK201427	Li et al. (2020)
Lycodon rufozonatus	SYS r001365=CHS710	Yingpanxu, Hunan, China	MK201482	Li et al. (2020)
Lycodon rufozonatus	NIBRRP 117	Jucheon, Jeolla, Korea	JQ798788	Jeong et al. (2013)
Lycodon rufozonatus	NIBRRP 358	Bongnae-myeon, Jeolla Nam, Korea	JQ798786	Jeong et al. (2013)
Lycodon rufozonatus	NIBRRP 254	Uiseong, Gyeongsangbuk, Korea	JQ798787	Jeong et al. (2013)
Lycodon rufozonatus	HS2010037=CHS163	Xi'an, China	MK201306	Li et al. (2020)
Lycodon rufozonatus	GP 625	Liaoning, China	KC733196	Guo et al. (2013)
Lycodon rufozonatus	MMS11951	Jeju, South Korea	OQ200119	Shin et al. (2024)
Lycodon rufozonatus	21LRCC001	Chuncheon, South Korea	OQ200120	Shin et al. (2024)
Lycodon rufozonatus	21LRCC002	Chuncheon, South Korea	OQ200121	Shin et al. (2024)
Lycodon rufozonatus	21LYCC009a	Chuncheon, South Korea	OQ200122	Shin et al. (2024)
Lycodon rufozonatus	SYSr000909	Huangshan, Anhui, China	MK201427	Li et al. (2020)
Lycodon rufozonatus	CHS164	Huangshan, Anhui, China	MK201307	Li et al. (2020)
Lycodon rufozonatus	MANU 2455982	China	KF148622	Li & Xu unpublished
Lycodon rufozonatus	LSUMZ 44977	China	AF471063	Lawson et al. (2005)
Lycodon duytan **sp. nov.**	DTU 542	Pu Mat NP, Nghe An, Vietnam	PQ863685	this study
Lycodon duytan **sp. nov.**	DTU 543	Cuc Phuong NP, Ninh Binh, Vietnam	PQ863686	this study
Lycodon sealei	KU 327571	Palawan, Palawan, Philippines	KC010384	Siler et al. (2013)
Lycodon semicarinatus	KUZ 28044	Japan	LC640371	Kambayashi et al. (2022)
Lycodon septentrionalis	KIZ YPX46117	Pale, Sagaing, Myanmar	MW199801	Che et al. (2020)
Lycodon serratus	KIZ 038335	Deqin, Yunnan, China	MW353746	Wang et al. (2021)
Lycodon sidiki	MZB 5980	Ache, Sumatra, Indonesia	KX822583	Wostl et al. (2017)
Lycodon stormi	JAM 7487	Air Terjun Moramo, Sulawesi, Indonesia	KC010380	Siler et al. (2013)
Lycodon striatus	-	Savandurga, Karnataka, India	MW006489	Kalki et al. (2020)
Lycodon subcinctus	LSUHC 5016	Pahang, Malaysia	KC010382	Siler et al. (2013)
Lycodon cf. *tessellatus*	KU 305141	Semirara, Antique, Philippines	KC010348	Siler et al. (2013)
Lycodon truongi	SIEZC 20249	Song Giang, Khanh Hoa, Vietnam	OM674282	Nguyen et al. (2022)
Lycodon yunnanensis	GP3288	Jinping, Yunnan, China	KP901020	Guo et al. (2015)
Lycodon zawi	CAS 239944	Kaaukpyu, Rakhine, Myanmar	KC010386	Siler et al. (2013)
Lycodon zayuensis	YBU 20694	Chayu, Tibet, China	OP434398	Luy et al. (2022)
Oligodon maculatus	KU 321699	Mindanao, Philippines	KC010387	Lawson et al. (2005)

Supplementary references for Suppl. Table S3

Che J, Jiang K, Yan F, Zhang YP (2020) Amphibians and reptiles in Tibet: diversity and evolution. Science Press, Beijing, China. [In Chinese].

Dubey B, Meganathan PR, Vidal N, Haque I (2012) Molecular evidence for the nonmonophyly of the Asian natricid genus *Xenochrophis* (Serpentes, Colubroidea) as inferred from mitochondrial and nuclear genes. Journal of Herpetology 46(2): 263–268. https://doi.org/10.1670/10-116.

Figueroa A, McKelvy AD, Grismer LL, Bell CD, Lailvaux SP (2016) A species-level phylogeny of extant snakes with description of a new Colubrid subfamily and genus. PLoS ONE 11(9): e0161070. https://doi.org/10.1371/journal.pone.0161070.

Grismer LL, Quah ESH, Anuar MSS, Muin MA, Wood PL, Nor SAM (2014) A diminutive new species of cave-dwelling Wolf Snake (Colubridae: *Lycodon* Boie, 1826) from Peninsular Malaysia. Zootaxa 3815(1): 51–67. https://doi.org/10.11646/zootaxa.3815.1.3.

Guo P, Zhang L, Liu Q, Li C, Pyron RA, Jiang K, Burbrink FT (2013) *Lycodon* and *Dinodon*: one genus or two? Evidence from molecular phylogenetics and morphological comparisons. Molecular Phylogenetics and Evolution 68(1): 144–149. https://doi.org/10.1016/j.ympev.2013.03.008.

Janssen HY, Pham CT, Ngo HT, Le MD, Nguyen TQ, Ziegler T (2019) A new species of *Lycodon* Boie, 1826 (Serpentes, Colubridae) from northern Vietnam. ZooKeys 875: 1–29. https://doi.org/10.3897/zookeys.875.35933

Jeong TJ, Jun J, Han S, Kim HT, Oh K, Kwak M (2013) DNA barcode reference data for the Korean herpetofauna and their applications. Molecular Ecology Resources 13(6): 1019–1032. https://doi.org/10.1111/1755-0998.12055

Kalki Y, Gowda S, Agnivamshi M, Singh K, Patel H, Mirza ZA (2020) On the taxonomy and systematics of the recently described *Lycodon deccanensis* Ganesh, Deuti, Punith, Achyuthan, Mallik, Adhikari, Vogel, 2020 (Serpentes, Colubridae) from India. Evolutionary Systematics 4(2): 109–118. https://doi.org/10.3897/evolsyst.4.60570

Kambayashi C, Kakehashi R, Sato Y, Mizuno H, Tanabe H, Rakotoarison A, Kunzel S, Furuno N, Ohshima K, Kumazawa Y, Nagy ZT, Mori A, Allison A, Donnellan SC, Ota H, Hoso M, Yanagida T, Sato H, Vences M, Kurabayashi A (2022) Geography-dependent horizontal gene transfer from vertebrate predators to their prey. Molecular Biology and Evolution 39(4): msac052. https://doi.org/10.1093/molbev/msac052.

Lawson RB, Slowinski JB, Crother BI, Burbrink FT (2005) Phylogeny of the Colubroidea (Serpentes): New evidence from mitochondrial and nuclear genes. Molecular Phylogenetics and Evolution 37(2): 581–601. https://doi.org/10.1016/j.ympev.2005.07.016

Lei J, Sun XY, Jiang K, Vogel G, Booth DT, Ding L (2014) Multilocus phylogeny of *Lycodon* and the taxonomic revision of *Oligodon multizonatum*. Asian Herpetological Research 5(1): 26–37. https://doi.org/10.3724/SP.J.1245.2014.00026

Li JN, Liang D, Wang YY, Guo P, Huang S, Zhang P (2020) A large scale systematic framework of Chinese snakes based on a unified multilocus marker system. *Molecular* Phylogenetics and Evolution 148: 106807. https://doi.org/10.1016/j.ympev.2020.106807

Luu VQ, Bonkowski M, Nguyen TQ, Le MD, Calame T, Ziegler T (2018) A new species of *Lycodon* Boie, 1826 (Serpentes: Colubridae) from central Laos. Revue Suisse de Zoologie 152(2): 263–276. https://doi.org/10.5281/zenodo.1414221

Lyu B, Li Q, Li KE, Li L, Shu FU, Wu Y, Guo P (2022) Expanded morphological description of the recently described *Lycodon zayuensis* (Serpentes: Colubridae). Zootaxa 5213(2): 159–168. https://doi.org/10.11646/zootaxa.5213.2.4.

Nawani S, Deepak V, Gautam KB, Gupta SK, Boruah B, Das A (2021) Systematic status of the rare Himalayan wolf snake *Lycodon mackinnoni* Wall, 1906 (Serpentes: Colubridae). Zootaxa 4966(3): 305–320. https://doi.org/10.11646/zootaxa.4966.3.3

Pyron RA, Kandambi HKD, Hendry CR, Pushpamal V, Burbrink FT, Somaweera R (2013) Genus-level phylogeny of snakes reveals the origins of species richness in Sri Lanka. Molecular Phylogenetics and Evolution 66: 969–978. https://doi.org/10.1016/j.ympev.2012.12.004

Siler CD, Oliveros CH, Santanen A, Brown RM (2013) Multilocus phylogeny reveals unexpected diversification patterns in Asian Wolf Snakes (genus *Lycodon*). Zoologica Scripta 42: 262–277. https://doi.org/10.1111/zsc.12007

Wang K, Yu ZB, Vogel G, Che J (2021) Contribution to the taxonomy of the genus *Lycodon* H. Boie in Fitzinger, 1827 (Reptilia: Squamata: Colubridae) in China, with description of two new species and resurrection and elevation of *Dinodon septentrionale chapaense* Angel, Bourret, 1933. Zoological Research 42(1): 62–86. https://doi.org/10.24272/j.issn.2095-8137.2020.286

Wostl E, Hamidy A, Kurniawan N, Smith EN (2017) A new species of wolf snake of the genus *Lycodon* H. Boie in Fitzinger (Squamata: Colubridae) from the Aceh Province of northern Sumatra, Indonesia. Zootaxa 4276: 530–553. https://doi.org/10.11646/zootaxa.4276.4.6

Suppl. Table S4. The species delimitation results of Bayesian PTP (bPTP) and ASAP with Jukes-Cantor distance (JC69) and Kimura (K80) ts/tv 2.0.

**bPTP result**
Max likilhood partition
Species 1 (support = 1.000)
KC010380_1
Species 2 (support = 1.000)
KC010376_1
Species 3 (support = 1.000)
KX822584_1
Species 4 (support = 1.000)
KC010368_1
Species 5 (support = 1.000)
KC347486_1
Species 6 (support = 1.000)
KC347476_1
Species 7 (support = 1.000)
KX660473_1
Species 8 (support = 1.000)
KC010362_1
Species 9 (support = 0.999)
MW199788_1
Species 10 (support = 1.000)
KC010367_1
Species 11 (support = 1.000)
MW862977_1
Species 12 (support = 1.000)
LC640371_1
Species 13 (support = 0.999)
MW006489_1
Species 14 (support = 0.999)
MW006486_1
Species 15 (support = 0.999)
MW006488_1
Species 16 (support = 0.996)
KC733200_1
Species 17 (support = 0.989)

MH669272_1
Species 18 (support = 0.987)
MW353745_1
Species 19 (support = 0.987)
KP901020_1
Species 20 (support = 0.996)
KC010365_1
Species 21 (support = 0.996)
KX822583_1
Species 22 (support = 0.995)
KX660474_1
Species 23 (support = 0.995)
KX660499_1
Species 24 (support = 0.992)
HQ735416_1
Species 25 (support = 0.992)
KC010386_1
Species 26 (support = 0.997)
KC010374_1
Species 27 (support = 0.993)
MW353748_1
Species 28 (support = 0.975)
MT602075_1
Species 29 (support = 0.940)
MW353739_1
Species 30 (support = 0.908)
MW199801_1
Species 31 (support = 0.987)
KC010353_1
Species 32 (support = 0.987)
KJ607889_1
Species 33 (support = 0.987)
KC010382_1
Species 34 (support = 0.971)
KC010384_1
Species 35 (support = 0.971)
MT625859_1
Species 36 (support = 0.874)
OP434398_1

Species 37 (support = 0.987)
MT845093_1
Species 38 (support = 0.874)
KC733207_1
Species 39 (support = 0.874)
OM674282_1
Species 40 (support = 0.832)
KC733201_1
Species 41 (support = 0.694)
KC010348_1
Species 42 (support = 0.694)
KC010356_1
Species 43 (support = 0.565)
KF732926_1
Species 44 (support = 0.565)
MW353746_1
Species 45 (support = 0.383)
OM674283_1,OM674284_1
Species 46 (support = 0.449)

KC733194_1,JQ798788_1,JQ798786_1,KC733196_1,OQ200120_1,OQ200122_1,OQ200121_1,JQ798787_1,MK201306_1,MK201427_1,AF471063_1,KF148622_1,KF732924_1,MT625858_1,OQ200119_1,MK201307_1,MK201482_1,MT625860_1,JF827672_1
Species 47 (support = 0.391)
MK201531_1
Species 48 (support = 0.373)
XXXXXXX2_1,XXXXXXX1_1
Species 49 (support = 0.566)
KC010351_1
Species 50 (support = 0.533)
KC010360_1,KC010344_1
Species 51 (support = 0.337)
KC733210_1
Species 52 (support = 0.337)
MH669268_1

Suppl. Table S4 (continued)

ASAP-JC result

Partition 6
Asap-Score: 11.000000
Proba: 8.622754e-01
Nb subsets with recursion:54 (without recursion: 53)

Subset[1] n: 1 ;id: HQ735416.1 aulicus No number Snake Transit Jabalpur Madhya Pradesh India
Subset[2] n: 1 ;id: KC010356.1 capucinus sp2 LSUHC 9277 Nam Du Kien Giang Vietnam
Subset[3] n: 1 ;id: KC010348.1 tessellatus cf KU 305141 Semirara Antique Philippines alicus
Subset[4] n: 1 ;id: KC010353.1 butleri LSUHC 9136 Bukit Larut Perak Malaysia
Subset[5] n: 1 ;id: KJ607889.1 cavernicolus LSUHC 9985 Gua Wang Burma Perlis Malaysia
Subset[6] n: 1 ;id: KX660473.1 davisonii cf FMNH 255034 Boualapha Khammouan
Subset[7] n: 1 ;id: KC347476.1 nympha RAP 0536 Kandalam Matale Sri Lanka
Subset[8] n: 1 ;id: KX660499.1 subannulatus LSUHC 5576 Sibu Johor Malaysia
Subset[9] n: 1 ;id: KX660474.1 tristrigatus FMNH 269033 Bintulu Sarawak Malaysia
Subset[10] n: 1 ;id: KC010376.1 effraenis LSUHC 9670 Kedah Malaysia
Subset[11] n: 1 ;id: MW199788.1 latifasciatus cf KIZ YPX46121 Medog Tibet China
Subset[12] n: 1 ;id: KC010365.1 fasciatus CAS 234875 Midat Chin Myanmar
Subset[13] n: 1 ;id: MW353748.1 gongshan KIZ 35112 Dulongjiang Nujiang Yunnan China
Subset[14] n: 1 ;id: KC733201.1 liuchengchaoi GP 2094 Nanling Guangdong China
Subset[15] n: 1 ;id: KF732926.1 multizonatus CIB 103 Luding Sichuan China
Subset[16] n: 1 ;id: MW353745.1 obvelatus KIZ 040146 Panzhihua Sichuan China
Subset[17] n: 1 ;id: MW353746.1 serratus KIZ 038335 Deqin Yunnan China
Subset[18] n: 1 ;id: KX822583.1 sidiki MZB 5980 Ache Sumatra Indonesia
Subset[19] n: 1 ;id: KP901020.1 yunnanensis GP 3288 Jinping Yunnan China
Subset[20] n: 1 ;id: KC733210.1 flavozonatus GP 2279 Guangdong China
Subset[21] n: 1 ;id: MH669268.1 meridionalis VNUF R.2017.54 Cuc Phuong Ninh Binh Vietnam
Subset[22] n: 1 ;id: OP434398.1 zayuensis YBU 20694 Chayu Tibet China
Subset[23] n: 1 ;id: MT602075.1 cathaya SYS r001542 Huaping Longsheng Guangxi China
Subset[24] n: 1 ;id: KC733207.1 futsingensis GP 2226 Guangdong China
Subset[25] n: 1 ;id: MT845093.1 pictus IEBR 4166 Trung Khanh Cao Bang Vietnam
Subset[26] n: 2 ;id: KC010344.1 alcalai KU 327847 Bataan Batanes Philippines KC010360.1 chrysoprateros KU 307720 Dalupiri Cagayan Philippines
Subset[27] n: 1 ;id: KC010351.1 bibonius KU 304589 Cagayan Philippines
Subset[28] n: 1 ;id: KC010362.1 dumerilii KU 305168 Dinagat Philippines
Subset[29] n: 1 ;id: KC010374.1 muelleri KU 323384 Luzon Aurora Philippines
Subset[30] n: 1 ;id: KX822584.1 albofuscus USMHC 1457 Penang Malaysia

Subset[31] n: 1 ;id: MH669272.1 banksi VNUF R.2015.20 Phou Hin Poun Khammouane Laos
Subset[32] n: 1 ;id: KC347486.1 carinatus RAP 0447 Kanneliya Galler Sri Lanka
Subset[33] n: 1 ;id: KC010367.1 jara CAS 235387 Kachin Myanmar
Subset[34] n: 1 ;id: KC010368.1 laoensis FMNH 258659 Salavan Laos
Subset[35] n: 1 ;id: LC640371.1 semicarinatus KU Z 28044 Japan
Subset[36] n: 1 ;id: KC010386.1 zawi CAS 239944 Kaaukpyu Rakhine Myanmar
Subset[37] n: 2 ;id: OM674284.1 anakradaya SIEZC 20248 Khanh Hoa anakradaya OM674283.1 anakradaya SIEZC 20247 Khanh Hoa
Subset[38] n: 1 ;id: KC733200.1 ruhstrati GP 2049 Guangdong China
Subset[39] n: 1 ;id: MW353739.1 chapaensis KIZ 034331 Xichou Wenshan Yunnan China
Subset[40] n: 1 ;id: MW199801.1 septentrionalis KIZ YPX46117 Pale Sagaing Myanmar
Subset[41] n: 1 ;id: OM674282.1 truongi SIEZC 20249 Song Giang Khanh Hoa Vietnam
Subset[42] n: 1 ;id: KC010380.1 stormi JAM 7487 Air Terjun Moramo Sulawesi Indonesia
Subset[43] n: 1 ;id: MW862977.1 bicolor ADR197 Mussoorie Uttarakhand India mackinnoni
Subset[44] n: 1 ;id: MW006486.1 deccanensis BNHS 3610 Devarayanadurga Tumkur Karnataka India
Subset[45] n: 1 ;id: MW006488.1 flavicollis No number Devarayanadurga Karnataka India
Subset[46] n: 1 ;id: MW006489.1 striatus Savandurga Karnataka India
Subset[47] n: 1 ;id: MT625859.1 neomaculatus SYS r001943 Shimentai National NR Yingde Guangdong China
Subset[48] n: 1 ;id: KC010384.1 sealei KU 327571 Palawan Palawan Philippines
Subset[49] n: 1 ;id: KC010382.1 subcinctus LSUHC 5016 Pahang Malaysia
Subset[50] n: 1 ;id: KC010387.1 Oligodon maculatus KU 321699
Subset[51] n: 1 ;id: MK201531.1 rosozonatus CHS 794 Jianfengling Hainan China
Subset[52] n: 2 ;id: XXXXXXX1.1 duytan DTU 542 Pu Mat Nghe An XXXXXXX2.1 duytan DTU 543 Cuc Phuong Ninh Binh
Subset[53] n: 10 ;id: JF827672.1 rufozonatus CIB 098274 China KC733194.1 rufozonatus GP 133 Sichuan China JQ798786.1 rufozonatus NIBRRP 358 Bongnae-myeon Jeolla Nam Korea JQ798787.1 rufozonatus NIBRRP 100254 Uiseong Gyeongsangbuk Korea KC733196.1 rufozonatus GP 625 Liaoning China OQ200120.1 rufozonatus 21LRCC001 OQ200121.1 rufozonatus 21LRCC002 OQ200122.1 rufozonatus 21LYCC009a JQ798788.1 rufozonatus NIBRRP 117 Jucheon Jeolla Korea MK201306.1 rufozonatus CHS 163 Xi an China
Subset[54] n: 9 ;id: KF732924.1 rufozonatus CIB 101 China MT625858.1 rufozonatus SYS r001770 Tiantai Zhejiang China AF471063.1 Lycodon rufozonatus OQ200119.1 rufozonatus MMS11951 KF148622.1 rufozonatus MK201427.1 rufozonatus CHS601 c MK201307.1 Lycodon rufozonatus voucher CHS164 c MK201482.1 rufozonatus CHS 710 Yingpanxu Hunan China MT625860.1 rufozonatus SYS r002061 Yangjifeng National NR Guixi Jiangxi

Suppl. Table S4 (continued)

ASAP-K80 result

Partition 7
Asap-Score: 11.500000
Proba: 8.403194e-01
Nb subsets with recursion:54 (without recursion: 53)

Subset[1] n: 1 ;id: HQ735416.1 aulicus No number Snake Transit Jabalpur Madhya Pradesh India
Subset[2] n: 1 ;id: KC010356.1 capucinus sp2 LSUHC 9277 Nam Du Kien Giang Vietnam
Subset[3] n: 1 ;id: KC010348.1 tessellatus cf KU 305141 Semirara Antique Philippines alicus
Subset[4] n: 1 ;id: KC010353.1 butleri LSUHC 9136 Bukit Larut Perak Malaysia
Subset[5] n: 1 ;id: KJ607889.1 cavernicolus LSUHC 9985 Gua Wang Burma Perlis Malaysia
Subset[6] n: 1 ;id: KX660473.1 davisonii cf FMNH 255034 Boualapha Khammouan
Subset[7] n: 1 ;id: KC347476.1 nympha RAP 0536 Kandalam Matale Sri Lanka
Subset[8] n: 1 ;id: KX660499.1 subannulatus LSUHC 5576 Sibu Johor Malaysia
Subset[9] n: 1 ;id: KX660474.1 tristrigatus FMNH 269033 Bintulu Sarawak Malaysia
Subset[10] n: 1 ;id: KC010376.1 effraenis LSUHC 9670 Kedah Malaysia
Subset[11] n: 1 ;id: MW199788.1 latifasciatus cf KIZ YPX46121 Medog Tibet China
Subset[12] n: 1 ;id: KC010365.1 fasciatus CAS 234875 Midat Chin Myanmar
Subset[13] n: 1 ;id: MW353748.1 gongshan KIZ 35112 Dulongjiang Nujiang Yunnan China
Subset[14] n: 1 ;id: KC733201.1 liuchengchaoi GP 2094 Nanling Guangdong China
Subset[15] n: 1 ;id: KF732926.1 multizonatus CIB 103 Luding Sichuan China
Subset[16] n: 1 ;id: MW353745.1 obvelatus KIZ 040146 Panzhihua Sichuan China
Subset[17] n: 1 ;id: MW353746.1 serratus KIZ 038335 Deqin Yunnan China
Subset[18] n: 1 ;id: KX822583.1 sidiki MZB 5980 Ache Sumatra Indonesia
Subset[19] n: 1 ;id: KP901020.1 yunnanensis GP 3288 Jinping Yunnan China
Subset[20] n: 1 ;id: KC733210.1 flavozonatus GP 2279 Guangdong China
Subset[21] n: 1 ;id: MH669268.1 meridionalis VNUF R.2017.54 Cuc Phuong Ninh Binh Vietnam
Subset[22] n: 1 ;id: OP434398.1 zayuensis YBU 20694 Chayu Tibet China
Subset[23] n: 1 ;id: MT602075.1 cathaya SYS r001542 Huaping Longsheng Guangxi China
Subset[24] n: 1 ;id: KC733207.1 futsingensis GP 2226 Guangdong China
Subset[25] n: 1 ;id: MT845093.1 pictus IEBR 4166 Trung Khanh Cao Bang Vietnam
Subset[26] n: 2 ;id: KC010344.1 alcalai KU 327847 Bataan Batanes Philippines KC010360.1 chrysoprateros KU 307720 Dalupiri Cagayan Philippines
Subset[27] n: 1 ;id: KC010351.1 bibonius KU 304589 Cagayan Philippines
Subset[28] n: 1 ;id: KC010362.1 dumerilii KU 305168 Dinagat Philippines
Subset[29] n: 1 ;id: KC010374.1 muelleri KU 323384 Luzon Aurora Philippines
Subset[30] n: 1 ;id: KX822584.1 albofuscus USMHC 1457 Penang Malaysia

Subset[31] n: 1 ;id: MH669272.1 banksi VNUF R.2015.20 Phou Hin Poun Khammouane Laos
Subset[32] n: 1 ;id: KC347486.1 carinatus RAP 0447 Kanneliya Galler Sri Lanka
Subset[33] n: 1 ;id: KC010367.1 jara CAS 235387 Kachin Myanmar
Subset[34] n: 1 ;id: KC010368.1 laoensis FMNH 258659 Salavan Laos
Subset[35] n: 1 ;id: LC640371.1 semicarinatus KU Z 28044 Japan
Subset[36] n: 1 ;id: KC010386.1 zawi CAS 239944 Kaaukpyu Rakhine Myanmar
Subset[37] n: 2 ;id: OM674284.1 anakradaya SIEZC 20248 Khanh Hoa anakradaya OM674283.1 anakradaya SIEZC 20247 Khanh Hoa
Subset[38] n: 1 ;id: KC733200.1 ruhstrati GP 2049 Guangdong China
Subset[39] n: 1 ;id: MW353739.1 chapaensis KIZ 034331 Xichou Wenshan Yunnan China
Subset[40] n: 1 ;id: MW199801.1 septentrionalis KIZ YPX46117 Pale Sagaing Myanmar
Subset[41] n: 1 ;id: OM674282.1 truongi SIEZC 20249 Song Giang Khanh Hoa Vietnam
Subset[42] n: 1 ;id: KC010380.1 stormi JAM 7487 Air Terjun Moramo Sulawesi Indonesia
Subset[43] n: 1 ;id: MW862977.1 bicolor ADR197 Mussoorie Uttarakhand India mackinnoni
Subset[44] n: 1 ;id: MW006486.1 deccanensis BNHS 3610 Devarayanadurga Tumkur Karnataka India
Subset[45] n: 1 ;id: MW006488.1 flavicollis No number Devarayanadurga Karnataka India
Subset[46] n: 1 ;id: MW006489.1 striatus Savandurga Karnataka India
Subset[47] n: 1 ;id: MT625859.1 neomaculatus SYS r001943 Shimentai National NR Yingde Guangdong China
Subset[48] n: 1 ;id: KC010384.1 sealei KU 327571 Palawan Palawan Philippines
Subset[49] n: 1 ;id: KC010382.1 subcinctus LSUHC 5016 Pahang Malaysia
Subset[50] n: 1 ;id: KC010387.1 Oligodon maculatus KU 321699
Subset[51] n: 1 ;id: MK201531.1 rosozonatus CHS 794 Jianfengling Hainan China
Subset[52] n: 2 ;id: XXXXXXX1.1 duytan DTU 542 Pu Mat Nghe An XXXXXXX2.1 duytan DTU 543 Cuc Phuong Ninh Binh
Subset[53] n: 10 ;id: JF827672.1 rufozonatus CIB 098274 China KC733194.1 rufozonatus GP 133 Sichuan China JQ798786.1 rufozonatus NIBRRP 358 Bongnae-myeon Jeolla Nam Korea JQ798787.1 rufozonatus NIBRRP 100254 Uiseong Gyeongsangbuk Korea KC733196.1 rufozonatus GP 625 Liaoning China OQ200120.1 rufozonatus 21LRCC001 OQ200121.1 rufozonatus 21LRCC002 OQ200122.1 rufozonatus 21LYCC009a JQ798788.1 rufozonatus NIBRRP 117 Jucheon Jeolla Korea MK201306.1 rufozonatus CHS 163 Xi an China
Subset[54] n: 9 ;id: KF732924.1 rufozonatus CIB 101 China MT625858.1 rufozonatus SYS r001770 Tiantai Zhejiang China AF471063.1 Lycodon rufozonatus OQ200119.1 rufozonatus MMS11951 KF148622.1 rufozonatus MK201427.1 rufozonatus CHS601 c MK201307.1 Lycodon rufozonatus voucher CHS164 c MK201482.1 rufozonatus CHS 710 Yingpanxu Hunan China MT625860.1 rufozonatus SYS r002061 Yangjifeng National NR Guixi Jiangxi

Suppl. Table S5. Acronyms of museums and other natural history collections mentioned in this study.

AMNH: American Museum of Natural History, New York, USA
BNHS: Bombay Natural History Society, Mumbai, India
CAS: California Academy of Sciences Museum, California, USA
CIB: Chengdu Institute of Biology, Chengdu, China
CPNP: Museum of the Cuc Phuong National Park, Ninh Binh, Vietnam
CWNU: Amphibians and Reptiles Museum, College of Life Sciences, West China Normal University, Nanchong, Sichuan, China
DL: Ding Lee's private collection, Chengdu, China
DTU: Duy Tan University, Da Nang, Vietnam
FMNH: Field Museum of Natural History, Chicago, USA
GP: Guo Peng private collection, Yibin University, Yibin, China
IEBR: Institute of Ecology and Biological Resources, Hanoi, Vietnam
KIZ: Museum of the Kunming Institute of Zoology, Yunnan, China
KU: Museum of Natural History, University of Kansas, Lawrence, Kansas, USA
KUZ: Ryukyus University, Okinawa, Japan
LSUHC: La Sierra University Herpetological Collections, Riverside, CA, USA
MNHN: Muséum national d'Histoire naturelle, Paris, France
MVZ: Museum of Vertebrate Zoology, University of California at Berkeley, Berkeley, California, USA
MZB: Laboratory of Herpetology at the Museum Zoologicum Bogoriense, Indonesian Institute of Sciences, Indonesia
MZMU: Departmental Museum of Zoology, Mizoram University, Mizoram, India
NHMUK: The Natural History Museum, London, UK
NHMW: Naturhistorisches Museum Wien, Vienna, Austria
NMBE: Naturhistorisches Museum Bern, Bern, Switzerland
RAP: R. Alexander Pyron private collection, The George Washington University, Washington, USA
RMNH: Naturalis-Nationaal Natuurhistorisch Museum [formerly Rijksmuseum van Natuurlijke Historie], Leiden, Netherlands
ROM: Royal Ontario Museum, Toronto, Ontario, Canada
SMF: Naturmuseum Senckenberg, Frankfurt am Main, Germany; SYS: Sun Yat-sen University, Guangzhou, Guangdong, China
TIU: Science College Museum, Tokyo Imperial University, Tokyo, Japan
USNM: National Museum of Natural History [formerly United States National Museum], Smithsonian Institution, Washington, USA
VNMN: Vietnam National Museum of Nature, Hanoi, Vietnam
VNUF: Vietnam National University of Forestry , Hanoi, Vietnam
WII-ADR: Abhijit Das Reptile collection in Wildlife Institute of India, Dehradun, India
ZFMK: Zoologisches Forschungsmuseum Alexander Koenig, Bonn, Germany
ZMB: Zoologisches Museum für Naturkunde der Humboldt-Universität zu Berlin, Berlin, Germany

Suppl. Table S6. Uncorrected ("p") distance matrix showing percentage pairwise genetic divergence (cytochrome *b*) between *Lycodon duytan* **sp. nov.** and closely related species.

Species	1	2	3	4	5	6	7	8	9	10	11	12	13	14
1. *Lycodon duytan* **sp. nov.**	-													
2. *Lycodon rosozonatus*	3.17–3.39	-												
3. *Lycodon rufozonatus*	3.17–4.74	3.17–4.29	-											
4. *Lycodon anakradaya*	6.10–6.34	5.88	4.73–6.34	-										
5. *Lycodon meridionalis*	9.68–9.93	10.47	9.17–10.47	10.47	-									
6. *Lycodon flavozonatus*	8.99–9.24	9.77	7.99–9.24	9.98	2.72	-								
7. *Lycodon futsingensis*	10.76–11.03	10.53	9.99–11.03	10.26	8.78	8.78	-							
8. *Lycodon truongi*	10.48–10.74	10.50	9.97–11.30	11.50	7.52	6.34	6.34	-						
9. *Lycodon zayuensis*	10.78–11.05	10.54	9.74–11.08	11.07	9.97	7.31	9.48	9.97	-					
10. *Lycodon cathaya*	11.79–12.06	11.82	10.47–11.56	12.90	9.46	9.46	9.44	9.72	10.80	-				
11. *Lycodon semicarinatus*	11.00–11.27	10.24	9.45–11.83	11.55	10.42	10.45	12.82	12.25	9.97	10.75	-			
12. *Lycodon septentrionalis*	11.00–11.27	11.83	10.48–11.84	10.76	9.47	8.24	8.24	7.25	10.54	9.72	12.10	-		
13. *Lycodon chapaensis*	11.66–11.93	11.42	10.32–10.42	11.08	7.28	7.02	7.02	6.53	11.60	8.71	12.03	6.67	-	
14. *Lycodon banksi*	12.97–13.25	12.18	12.15–13.29	11.63	9.74	8.71	8.71	9.95	10.04	10.77	11.30	10.78	8.48	-

Suppl. Table S7. List of localities of the *Lycodon duytan* **sp. nov.**, *L. walli* **stat. nov.**, *L rufozonatus*, and *L. rosozonatus* appearing on Figure 1. Symbols: (1) = Number on the map; (2) = Verified by morphology data (yes/no); (3) = Verified by molecular data (yes/no).

(1)	(2)	(3)	Location	Sources
Lycodon duytan **sp. nov.**				
1	yes	yes	Pu Mat NP, Nghe An, Vietnam (type locality)	This study
2	yes	no	Vu Quang NP, Ha Tinh, Vietnam	This study
3	yes	no	Pu Hoat NR, Nghe An, Vietnam	This study
4	yes	no	Nam Dong NR, Thanh Hoa, Vietnam	This study
5	yes	yes	Cuc Phuong NP, Ninh Binh, Vietnam	This study
Lycodon walli **stat. nov.**				
1	yes	no	Ishigaki Is, Yaeyama, Ryukyu, Japan (type locality)	Goris & Maeda (2004); This study
2	yes	no	Iriomote Is, Yaeyama, Ryukyu, Japan	Goris & Maeda (2004); This study
3	yes	no	Miyako Is, Yaeyama, Ryukyu, Japan	Goris & Maeda (2004); This study
Lycodon rufozonatus				
1	yes	yes	Zhoushan, Zhejiang, China (type locality of *Lycodon rufo-zonatus*)	Zhao et al. (1998); Wu et al. (2023); This study
2	yes	yes	Ningbo, Zhejiang, China (type locality of *Coronella striata*)	Zhao et al. (1998); Wu et al. (2023); This study
3	yes	yes	Mt. Tiantai, Taizhou, Zhejiang, China	Wu et al. (2023); https:/www.inaturalist.org/observations/167309982
4	yes	yes	Hangtou, Zhejiang, China	Wu et al. (2023); This study
5	yes	yes	Longyou, Zhejiang, China	Zhao et al. (1998); Wu et al. (2023)
6	yes	yes	Longquan, Zhejiang, China	Zhao et al. (1998); Wu et al. (2023)
7	yes	yes	Jingning, Zhejiang, China	Zhao et al. (1998); Wu et al. (2023)
8	yes	yes	Wencheng, Zhejiang, China	Zhao et al. (1998); Wu et al. (2023)
9	yes	no	Shanghai, China	This study; https:/www.inaturalist.org/observations/216420704
10	yes	no	Yangcheng, Jiangsu, China	Zhao et al. (1998); https:/www.inaturalist.org/observations/198972014
11	yes	no	Changzhou, Jiangsu, China	Zhao et al. (1998); https:/www.inaturalist.org/observations/183333339
12	yes	no	Nanjing, Jiangsu, China	Zhao et al. (1998); https:/www.inaturalist.org/observations/237799791
13	yes	no	Yangcheng, Jiangsu, China	Zhao et al. (1998); https:/www.inaturalist.org/observations/122973735
14	yes	no	Suqian, Jiangsu, China	Zhao et al. (1998); https:/www.inaturalist.org/observations/123864644
15	yes	no	Lianyungang, Jiangsu, China	Zhao et al. (1998); https:/www.inaturalist.org/observations/85484455
16	yes	no	Xuzhou, Jiangsu, China	Zhao et al. (1998); https:/www.inaturalist.org/observations/189152819
17	yes	no	Linyi, Shangdong, China	Zhao et al. (1998); https:/www.inaturalist.org/observations/198627155
18	yes	no	Qingdao, Shandong, China	Zhao et al. (1998); This study; https:/www.inaturalist.org/observations/131055650
19	yes	no	Weihai, Shandong, China	Zhao et al. (1998); https:/www.inaturalist.org/observations/199242549
20	yes	no	Weifang, Shandong, China	Zhao et al. (1998); https:/www.inaturalist.org/observations/180653469

21	yes	no	Jinan, Shandong, China	Zhao et al. (1998); https:/www.inaturalist.org/observations/203152172
22	yes	no	Liaocheng, Shandong, China	Zhao et al. (1998); https:/www.inaturalist.org/observations/216136145
23	yes	no	Baoding, Hebei, China	Zhao et al. (1998); https:/www.inaturalist.org/observations/235874934
24	yes	no	Tangshan, Hebei, China	Zhao et al. (1998); https:/www.inaturalist.org/observations/135089988
25	yes	no	Beijing, Beijing, China	Zhao et al. (1998); https:/www.inaturalist.org/observations/172449893
26	yes	no	Beijing, Beijing, China	Zhao et al. (1998); https:/www.inaturalist.org/observations/221967722
27	yes	no	Dalian, Liaoning, China	Zhao et al. (1998); https:/www.inaturalist.org/observations/181001911
28	yes	no	Tonghua, Jilin, China	Zhao et al. (1998); https:/www.inaturalist.org/observations/97630235
29	yes	no	Jilin, Jilin, China	Zhao et al. (1998); https:/www.inaturalist.org/observations/222659300
30	yes	no	Yuncheng, Shanxi, China	Zhao et al. (1998); https:/www.inaturalist.org/observations/96886296
31	yes	no	Yulin, Shaaaxi, China	Zhao et al. (1998); https:/www.inaturalist.org/observations/26050297
32	yes	yes	Xian Shaanxi, China	Zhao et al. (1998); Wu et al. (2023)
33	yes	yes	Baoji, Shaanxi, China	Zhao et al. (1998); Li et al. (2020); This study
34	yes	yes	Taibai, Shaanxi, China	Zhao et al. (1998); Wu et al. (2023)
35	yes	yes	Luanchuan, Henan, China	Zhao et al. (1998); Wu et al. (2023)
36	yes	yes	Xixia, Henan, China	Zhao et al. (1998); Wu et al. (2023)
37	yes	yes	Baiton, Henan, China	Zhao et al. (1998); Wu et al. (2023)
38	yes	yes	Tongbai, Henan, China	Zhao et al. (1998); Wu et al. (2023)
39	yes	yes	Xin, Henan, China	Zhao et al. (1998); Wu et al. (2023)
40	yes	yes	Bozhou, Anhui, China	Zhao et al. (1998); Wu et al. (2023)
41	yes	no	Chozhou, Anhui, China	Zhao et al. (1998); https:/www.inaturalist.org/observations/168026238
42	yes	yes	Huoshan Anhui, China	Zhao et al. (1998); Wu et al. (2023)
43	yes	no	Xuancheng, Anhui, China	Zhao et al. (1998); https:/www.inaturalist.org/observations/208070855
44	yes	yes	Huangshan, Anhui, China	Zhao et al. (1998); Li et al. (2020); Wu et al. (2023)
45	yes	yes	Xiuning, Anhui, China	Zhao et al. (1998); Wu et al. (2023)
46	yes	yes	Lu Mt., Jiujiang, Jiangxi, China	Zhao et al. (1998); Wu et al. (2023)
47	yes	no	Yichun, Jiangxi, China	Zhao et al. (1998); https:/www.inaturalist.org/observations/171516426
48	yes	yes	Guxi, Jiangxi, China	Zhao et al. (1998); Wu et al. (2023)
49	yes	no	Pingxiang, Jiangxi, China	Zhao et al. (1998); https:/www.inaturalist.org/observations/185592390
50	yes	no	Jian, Jiangxi, China	Zhao et al. (1998); https:/www.inaturalist.org/observations/239595922
51	yes	no	Ganzhou, Jiangxi, China	Zhao et al. (1998); https:/www.inaturalist.org/observations/122953377
52	yes	yes	Wuyi Mt, Fujian, China	Zhao et al. (1998); Wu et al. (2023)
53	yes	no	Fuzhou, Fujian, China	Zhao et al. (1998); https:/www.inaturalist.org/observations/169163920
54	yes	no	Sanming, Fujian, China	Zhao et al. (1998); https:/www.inaturalist.org/observations/113428857
55	yes	no	Longyan, Fujian, China	Zhao et al. (1998); https:/www.inaturalist.org/observations/126018758
56	yes	no	Chebaling, Guangdong, China	Zhao et al. (1998); This study
57	yes	no	Gungzhou, Guangdong, China	Zhao et al. (1998); https:/www.inaturalist.org/observations/66644105
58	yes	no	Shanzhen, Guangdong, China	Zhao et al. (1998); https:/www.inaturalist.org/observations/190008457
59	yes	yes	Shiyan, Hubei, China	Zhao et al. (1998); Wu et al. (2023)

60	yes	yes	Yichang, Hubei, China	Zhao et al. (1998); Wu et al. (2023)
61	yes	yes	Dangyang, Hubei, China	Zhao et al. (1998); Wu et al. (2023)
62	yes	yes	Guangyuan, Sichuan, China	Zhao et al. (1998); Wu et al. (2023)
63	yes	yes	Yingjing, Sichuan, China	Zhao et al. (1998); Wu et al. (2023)
64	yes	yes	Shimian, Sichuan, China	Zhao et al. (1998); Wu et al. (2023)
65	yes	no	Panzhuhua, Sichuan, China	Zhao et al. (1998); https:/www.inaturalist.org/observations/189134620
66	yes	yes	Jinyang, Sichuan, China	Zhao et al. (1998); Wu et al. (2023)
67	yes	yes	Beibei, Chongqing, China	Zhao et al. (1998); Wu et al. (2023)
68	yes	yes	Qianjiang, Chongqing, China	Zhao et al. (1998); Wu et al. (2023)
69	yes	yes	Youyang, Chongqing, China	Zhao et al. (1998); Wu et al. (2023)
70	yes	no	Bamian, Hunan, China	Zhao et al. (1998); This study
71	yes	yes	Taoyuan, Hunan, China	Zhao et al. (1998); Wu et al. (2023)
72	yes	no	Changsha, Hunan, China (type locality of *Dinodon rufozonatum williamsi*)	Zhao et al. (1998); https:/www.inaturalist.org/observations/147315243
73	yes	yes	Guidong, Hunan, China	Zhao et al. (1998); Li et al. (2020); Wu et al. (2023)
74	yes	yes	Dongbei, Hanshou, Hunan, China	Zhao et al. (1998); Wu et al. (2023)
75	yes	yes	Suiyang, Guizhou, China	Zhao et al. (1998); Wu et al. (2023)
76	yes	no	Tongren, Guizhou, China	Zhao et al. (1998); https:/www.inaturalist.org/observations/229064053
77	yes	no	Qiandongnan, Guizhou, China	Zhao et al. (1998); https:/www.inaturalist.org/observations/177989781
78	yes	yes	Xingan Guangxi, China	Zhao et al. (1998); Wu et al. (2023)
79	yes	yes	Yulin, Guangxi, China	Zhao et al. (1998); Wu et al. (2023)
80	yes	no	Zhaotong, Yunnan, China	Zhao et al. (1998); This study
81	yes	no	Dali, Yunnan, China (type locality of *Dinodon rufozonatum yunnanense*)	Zhao et al. (1998); This study
82	yes	no	Chuxiong Yi, Yunnan, China	Zhao et al. (1998); https:/www.inaturalist.org/observations/96198966
83	yes	no	Kunming, Yunnan, China	Zhao et al. (1998); https:/www.inaturalist.org/observations/221051612
84	yes	no	Wenshan, Yunnan, China	Zhao et al. (1998); https:/www.inaturalist.org/observations/178247864
85	yes	no	Keelung, Taiwan	This study; https:/www.inaturalist.org/observations/151915587
86	yes	no	Hualien, Taiwan	This study; https:/www.inaturalist.org/observations/144703213
87	yes	no	Nantou, Taiwan	This study; https:/www.inaturalist.org/observations/170248159
88	yes	no	Pingtung, Taiwan	This study; https:/www.inaturalist.org/observations/192336945
89	yes	no	Kaohsiung, Taiwan	This study; https:/www.inaturalist.org/observations/137226084
90	yes	no	Tainan, Taiwan	This study; https:/www.inaturalist.org/observations/169878113
91	yes	no	Penghu, Taiwan	This study; https:/www.inaturalist.org/observations/136797794
92	yes	no	Nezhino, Chedrnigovla, Russia	Maslov & Kotlobay (1998); Li et al. (2017)
93	yes	no	Incheon, South Korea	Shin et al. (2024); https:/www.inaturalist.org/observations/237415577
94	yes	no	Gangwon, South Korea	Shin et al. (2024); https:/www.inaturalist.org/observations/59775797
95	yes	no	Uiseong, Gyeongsangbuk, South Korea	Shin et al. (2024); https:/www.inaturalist.org/observations/59775797
96	yes	no	Busan, South Korea	Shin et al. (2024); https:/www.inaturalist.org/observations/28665897

97	yes	no	Bongnae-myeon, Jeolla Nam, South Korea	Shin et al. (2024); https:/www.inaturalist.org/observations/124527203
98	yes	no	Seogwipo, Jeju, South Korea	Shin et al. (2024); https:/www.inaturalist.org/observations/88911435
99	yes	no	Tsushima, Nagasaki, Japan	Goris & Maeda (2004); https:/www.inaturalist.org/observations/167979676
100	yes	no	Tay Yen Tu NR, Bac Giang, Vietnam	https:/www.inaturalist.org/observations/178507844
101	yes	no	Na Hang NR, Tuyen Quang, Vietnam	Orlov & Ryabov (2004); ROM 30814
102	yes	no	Tam Dao NP, Vinh Phuc, Vietnam	Orlov & Ryabov (2004); ROM 34615
Lycodon rosozonatus				
1	yes	no	Diaoluo Mt, Dali, Hainan, China (type locality)	Nguyen & Vogel (2025)
2	yes	no	Wuzhi Mt., Qiongzhong, Hainan, China	Nguyen & Vogel (2025)
3	yes	no	Baoting, Hainan, China	Nguyen & Vogel (2025)
4	yes	yes	Jianfengling NFP, Hainan, China	Nguyen & Vogel (2025)
5	yes	no	Changjiang Li, Hainan, China	Nguyen & Vogel (2025)
6	yes	no	Haikou, Hainan, China	Nguyen & Vogel (2025)
